# Interaction between post-tumor inflammation and vascular smooth muscle cell dysfunction in sepsis-induced cardiomyopathy

**DOI:** 10.3389/fimmu.2025.1560717

**Published:** 2025-04-10

**Authors:** Rui Liu, Lina Jia, Lin Yu, Detian Lai, Qingzhu Li, Bingyu Zhang, Enwei Guo, Kailiang Xu, Qiancheng Luo

**Affiliations:** ^1^ Department of Critical Care Medicine, Shanghai Pudong New Area Gongli Hospital, Shanghai, China; ^2^ Hebei Medical University, Shijiazhuang, China

**Keywords:** sepsis induced cardiomyopathy, DVL1, intratumor heterogeneity, oxidative stress, drug therapy, immunotherapy resistance, molecular docking, exercise training

## Abstract

**Background:**

Sepsis-induced cardiomyopathy (SIC) presents a critical complication in cancer patients, contributing notably to heart failure and elevated mortality rates. While its clinical relevance is well-documented, the intricate molecular mechanisms that link sepsis, tumor-driven inflammation, and cardiac dysfunction remain inadequately explored. This study aims to elucidate the interaction between post-tumor inflammation, intratumor heterogeneity, and the dysfunction of VSMC in SIC, as well as to evaluate the therapeutic potential of exercise training and specific pharmacological interventions.

**Methods:**

Transcriptomic data from NCBI and GEO databases were analyzed to identify differentially expressed genes (DEGs) associated with SIC. Weighted gene co-expression network analysis (WGCNA), gene ontology (GO), and KEGG pathway enrichment analyses were utilized to elucidate the biological significance of these genes. Molecular docking and dynamics simulations were used to investigate drug-target interactions, and immune infiltration and gene mutation analyses were carried out by means of platforms like TIMER 2.0 and DepMap to comprehend the influence of DVL1 on immune responsiveness.

**Results:**

Through the utilization of the datasets, we discovered the core gene DVL1 that exhibited remarkable up-regulated expression both in SIC and in diverse kinds of cancers, which were associated with poor prognosis and inflammatory responses. Molecular docking revealed that Digoxin could bind to DVL1 and reduce oxidative stress in SIC. The DVL1 gene module related to SIC was identified by means of WGCNA, and the immune infiltration analysis demonstrated the distinctive immune cell patterns associated with DVL1 expression and the impact of DVL1 on immunotherapeutic resistance.

**Conclusions:**

DVL1 is a core regulator of SIC and other cancers and, therefore, can serve as a therapeutic target. The present study suggests that targeted pharmacological therapies to enhance response to exercise regimens may be a novel therapeutic tool to reduce the inflammatory response during sepsis, particularly in cancer patients. The identified drugs, Digoxin, require further *in vivo* and clinical studies to confirm their effects on SIC and their potential efforts to improve outcomes in immunotherapy-resistant cancer patients.

## 1 Background

Sepsis-induced cardiomyopathy (SIC) is a common serious complication in critically ill cancer patients (1, 2). This condition leads to cardiac dysfunction, which is strongly associated with multiple organ failure, thereby increasing the risk of death (1, 2). Epidemiological studies have shown that SIC has a high incidence in critically ill patients, especially in cancer patients with accompanying sepsis, where its mortal (3, 4). This may be closely related to factors such as cancer-related chronic inflammation and immune dysfunction (5, 6). In recent years, an increasing number of studies have focused on the mechanisms of SIC in cancer patients, finding that the tumor microenvironment (TME) may interact with the immune imbalance related to sepsis, thus aggravating the development of SIC (7, 8). In addition, immunotherapy, chemotherapy, and targeted therapy may have an impact on the cardiovascular system and further increase the susceptibility to SIC in cancer patients (9, 10). In recent years, with technological advances, through RNA sequencing and spatial transcriptomics, scientists have revealed the functions and interactions of immune cells in the tumor microenvironment (11–13). Therefore, the systematic investigation of the molecular mechanisms of SIC and the exploration of potential therapeutic strategies may have important clinical implications for improving the prognosis of cancer patients (14). The pathomechanisms of SIC involve systemic inflammation, oxidative stress, mitochondrial dysfunction, as well as immune dysregulation (15, 16). In sepsis, a large number of proinflammatory cytokines (TNF- α, IL-6, IL-1 β) are released, triggering a cascade of inflammatory responses, leading to cardiomyocyte damage, mitochondrial collapse, deregulation of calcium homeostasis and, ultimately,myocardial contractile dysfunction (4, 17). In addition, oxidative stress and overproduction of ROS not only exacerbate cellular damage but may also further worsen the progression of SIC by inducing the loss of mitochondrial membrane potential and abnormal energy metabolism (18, 19). Increasing awareness of the role of cell death and metabolic regulation in disease progression is providing new targets and strategies for developing drugs (20–22). In cancer patients, the occurrence of SIC is also significantly affected by the tumor microenvironment. Macrophage polarization is closely related to changes in the immune microenvironment and crosstalk between immune cells (23, 24). Immunosuppressive cytokines secreted by tumors, such as TGF-β and IL-10, weaken the body’s ability to resist infection and inhibit the normal regulation of inflammatory response, leading to more severe sepsis-related myocardial injury (25, 26). At the same time, patients resistant to immunotherapy may exhibit more severe sepsis-associated cardiac damage, as TME-driven immune escape mechanisms may further contribute to inflammatory imbalance and immune hyperactivation in a septic setting (27, 28). Although the molecular mechanisms of SIC have been well studied in typical sepsis patients, the specific characteristics of SIC, immune-metabolic interactions, and their responses to existing treatment options in cancer patients are still underexplored (29, 30). Cancer-induced chronic inflammation and immunosuppression may exacerbate the development and progression of SIC, highlighting the importance of studying the role of tumor-associated immune regulation in the progression of sepsis-associated cardiomyopathy (31). In particular, considering the complexity of the cancer microenvironment, which includes different genetic, cellular, and tissue characteristics, leading to different therapeutic responses (32, 33).

The pathogenesis of SIC is closely related to the systemic inflammatory response, excessive cytokine release, and oxidative stress (34, 35). Hyperactivation of the immune system during sepsis leads to the massive release of pro-inflammatory cytokines such as tumor necrosis factor- α (TNF- α), interleukin-6 (IL-6), and interleukin-1 β (IL-1 β) (36, 37). These inflammatory mediators disrupt cardiac function, induce mitochondrial damage, dysregulation of calcium homeostasis, and promote cardiomyocyte apoptosis (38, 39). This inflammatory cascade is more complex in cancer patients, further exacerbated by tumor-induced immunosuppression. Tumor cells can secrete immunosuppressive cytokines such as transforming growth factor- β (TGF- β) and interleukin-10 (IL-10), which can inhibit the activation of cytotoxic immune cells and promote the formation of an immunotolerant tumor microenvironment (40, 41). Moreover, oxidative stress is also a key factor in the development of SIC. Reactive oxygen species (ROS) accumulation causes cell damage and apoptosis, which further deteriorates cardiac function and intensifies cardiac dysfunction (42, 43). Bioinformatics technologies have played a key role in the study of gene expression and regulatory mechanisms, providing an essential basis for understanding biological processes (44, 45).

The high incidence rate and the high intratumoral heterogeneity of tumors show the high pathological characteristics of SIC in cancer patients, as well as the high variability of treatment (46, 47). This heterogeneity is implicated by genetic variation and phenotypic heterogeneity, directly modulating the effectiveness of various treatment modalities, including immunotherapy. This variation presents a barrier to consistent clinical results (2, 48). Hence, it is crucial to understand the interaction between systemic inflammation, genetic variation, and the tumor microenvironment for the development of personalized treatment strategies for SIC in cancer patients (49, 50). While SIC is commonly associated with acute cardiac insufficiency and with symptoms such as hypotension and arrhythmia, clinically, it is associated with a decreased survival rate (1, 2). Continuous deterioration in cardiac function is associated with a marked reduction in quality of life. Still, it may also enhance the onset of complications, including chronic heart failure (CHF) and systemic multiorgan dysfunction (SOD) (51, 52). Thus, an in-depth understanding of the underlying pathogenic mechanisms of SIC will contribute to the exploration of therapeutic strategies with higher targeting and clinical applicability (53, 54). Individualized precision medicine intervention strategy combining several factors could be more beneficial to improve the therapeutic outcome of SIC (55). Despite extensive research on the inflammatory response and cellular damage mechanisms of SIC, there remain significant gaps in understanding the role of specific cell types, such as VSMCs, in sepsis-associated cardiac dysfunction (56). Most of the existing studies have focused on the effects of cytokine release on cardiomyocytes and ignored the role of VSMCs as an essential component of the cardiovascular system in the development of SIC (57, 58). VSMCs are mainly responsible for maintaining vascular stability and regulating vascular tone, enabling blood vessels to adapt to dynamic changes in blood pressure and blood flow (59, 60).VSMCs can transition from a contractile to a synthetic form in a sepsis-induced inflammatory setting, displaying both pro-inflammatory and pro-oxidative traits (61, 62). This pathological remodeling not only exacerbates the vascular dysfunction but also may further drive the progression of SIC by worsening the myocardial microcirculation and exacerbating cardiac inflammation (48, 63). Single-cell multi-omics analysis can analyze the complex physiological processes at the single-cell level, facilitate a deep understanding of the transplant immune mechanism, and provide support for the optimization of treatment options (64, 65). Therefore, studying the mechanism of VSMCs in sepsis-related cardiac dysfunction will not only contribute to a deep understanding of the pathogenesis of SIC but may also provide new potential therapeutic targets to lay the foundation for precise intervention of SIC.

In this study, the DVL1 protein has become a key point. DVL1 is a core regulator of the Wnt/β -catenin signaling pathway and is capable of regulating cell proliferation, differentiation, and apoptosis (48, 63). In various cancers, abnormal DVL1 expression is associated with poor prognosis, indicating its relevance in tumor biology (66). Advances in big data technologies and bioinformatics tools have driven the identification and validation of disease markers, especially in the areas of immune microenvironment, cellular signaling, and metabolic regulation (67, 68). In a septic setting, abnormal activation of DVL1 may affect macrophage polarization and disrupt the balance between proinflammatory M1 and immunosuppressive M2 macrophages, thereby exacerbating the systemic inflammatory response and inhibiting immune recovery, accelerating SIC progression (58). In cancer metabolism, DVL1 may regulate glycolysis and mitochondrial bioenergetic metabolism through the Wnt signaling pathway (58, 69). It is shown that DVL1 overexpression may enhance the metabolic plasticity of tumor-associated immune cells and cardiomyocytes, leading to abnormal glucose utilization and impaired oxidative phosphorylation, thus aggravating the myocardial energy crisis in SIC (31, 70). Moreover, DVL1 may affect cardiac dysfunction through oxidative stress associated with mitochondrial signaling (71, 72). Studies have shown that overexpression of DVL1 can increase reactive oxygen species (ROS) generation, directly disrupt cardiomyocytes, and perturb the mitochondrial membrane potential (73, 74). Wnt signaling can also affect mitochondrial biosynthesis by interacting with PGC-1 α, exacerbating metabolic and function decline in SIC (75, 76). Although DVL1 is recognized as a key factor in gastrointestinal cancer and SIC, its specific molecular roles and pathways in sepsis, cancer metabolism,and cardiac dysfunction have not been fully explored (70, 77). Further exploration of the mechanism by which DVL1 regulates SIC could provide new ideas for the treatment of SIC (69, 78).

This research seeks to examine how post-tumor inflammation interacts with VSMC dysfunction, aiming to bridge a significant gap in the understanding of SIC mechanisms. Subsequently, determine potential therapeutic targets to alleviate the treatment burden of SIC in individuals with cancer (56, 79). Paying particular attention to the DVL1 expression pattern in gastrointestinal cancers and evaluating the potential utility of FDA approved drugs in the treatment of SIC(37,38). This study also combines bibliometric analysis to judge the application trend of computer-assisted drug design in SIC-targeted therapy, and to provide a theoretical basis for the development of new therapeutic strategies in the future (80, 81). The application of network pharmacology and experimental validation methods in drug research provides new approaches and strategies for drug research and development, such as studying the mechanism of action and efficacy of a drug in the treatment of new diseases (82, 83).

New technologies and molecular research methods have played an essential role in disease research and treatment (84, 85). This study adopted a multi-level integration strategy to integrate transcriptomic data analysis (86). Through the deep mining of a large number of transcriptomic data, key genes and signaling pathways closely related to various physiological and pathological processes can be screened out, and potential targets for drug development can be identified (87, 88). Meanwhile, the key genes and signaling pathways associated with SIC were systematically analyzed (89, 90). The study of the regulatory mechanisms of multiple biological processes provides a basis for the optimization of intervention strategies (91–93). In recent years, precision-targeted intervention strategies targeting specific proteins or gene pathways have made breakthroughs in improving treatment specificity or clinical efficacy. The combination of transcriptomics with proteomics reveals key regulatory mechanisms of transcription factor networks and protein modification in disease (94, 95). This experimental study provides a successful experience for the individualized treatment of SIC (96, 97). This study further revealed the regulatory mechanism of VSMC dysfunction in SIC by post-tumor inflammation, focusing on immune cell infiltration, genetic heterogeneity, and its association with cardiovascular injury and assessing the potential of pharmacological intervention to alleviate pathological effects (98, 99). These findings enhance the comprehension of SIC’s pathogenic mechanisms and support the creation of personalized treatment approaches (43, 48). By integrating bioinformatics, transcriptomics, and pharmacological techniques, we will study the specific role of A fresh perspective on precision treatment for SIC patients provided by DVL1 in SIC (100, 101).

## 2 Materials and methods

### 2.1 Analysis of differential gene expression in sepsis-related cardiomyopathy

Transcriptomic datasets concerning sepsis-related cardiomyopathy were sourced from the NCBI and GEO databases (http://www.ncbi.nlm.nih.gov/geo/) (102, 103). For this study, two specific datasets were chosen: GSE172270, containing 20 peripheral blood samples from healthy individuals and 47 from patients with acute myocardial infarction (AMI), and GSE57065, which includes 25 samples from healthy controls alongside 28 from individuals diagnosed with sepsis (103, 104). Differential gene expression analysis was conducted using the limma package, applying a threshold of an adjusted P-value < 0.05 and |log2 fold change (log_2_FC)| > 1.00 to identify differentially expressed genes (DEGs). Volcano plots were employed to visualize the DEGs. To pinpoint common genes linked to sepsis-induced cardiomyopathy, Venn diagrams were used for comparative analysis. Subsequently, Gene Set Enrichment Analysis (GSEA) was performed to elucidate the functional roles of gene sets implicated in sepsis-related cardiomyopathy.

### 2.2 Development of a weighted gene co-expression network

To investigate gene expression patterns associated with sepsis-induced cardiomyopathy, genes exhibiting variance levels above the upper quartile were initially selected (90, 105). These selected genes were subsequently analyzed using the “WGCNA” package within R software to establish a weighted gene co-expression network (WGCNA) specific to sepsis-induced cardiomyopathy (55). The optimal soft-thresholding power (β) was determined by clustering the samples and using a scale-free network model to establish the association network by calculating the gene connection adjacency matrix. The topological overlap matrix (TOM) was used to measure gene similarity and create a hierarchical clustering tree. Dynamic tree-cutting methods were then employed to identify and refine gene modules from a constructed gene dendrogram. After the modules were established, the module eigengenes (MEs) for each cluster were calculated, followed by correlation with clinical characteristics of the AMI patients. To calculate the correlation between MEs and clinical traits, Pearson correlation was computed to find a module associated most closely with AMI, which was termed the key hub module. Further analyses were performed on this module, including validation of differentially expressed genes and functional enrichment. WGCNA was performed to screen hub genes, which were then overlapped with differentially expressed genes in sepsis-induced cardiomyopathy. This resulted in the identification of core genes closely associated with sepsis-induced cardiomyopathy. Using the clusterProfiler gene ontology (GO), common target genes for sepsis-induced cardiomyopathy were examined. R package in R and Perl. To elucidate the biological functions of these targets, this analysis involved the main GO categories, namely Cellular Component (CC), Molecular Function (MF), and Biological Process (BP). KEGG pathway enrichment analysis was also conducted using the clusterProfilerKEGG. R package, and pathway visualization performed using the path view package. The enrichment factor was used to assess the relevance of core pathway enrichments, revealing biological functions and signaling pathways that are involved in the pathophysiology of sepsis-induced cardiomyopathy.

### 2.3 Screening of FDA-approved drug library and molecular docking analysis

A library of 2,568 small molecules, all approved by the FDA (Food and Drug Administration), was selected for screening (106, 107). The molecular structures of these compounds were retrieved in SDF format from the DrugBank database (https://go.drugbank.com/) (108, 109). These molecules were imported into Chem3D software, where the structural optimization and energy minimization were performed using the MMFF94 force field (Halgren, 1999) within the Calculation module, and the optimized structures were saved in mol2 format. Core protein domains in pdb format were obtained from the PDB database (http://www.rcsb.org/), and preliminary processing, including solvent removal, was performed using PyMol software. Further preparations, including the addition of hydrogen atoms and assignment of charges, were executed using AutoDockTools, with both the protein targets and small molecules saved in pdbqt format. Grid parameters, including positions and dimensions, were defined, and the molecular docking between the ligands and target proteins was performed using Autodock-Vina. The results were analyzed using clustering heatmaps generated in R software, and PyMol was used for visualizing the docking interactions, yielding detailed molecular docking model diagrams.

### 2.4 Molecular dynamics simulation

Molecular dynamics (MD) simulations were performed using Gromacs version 2019.6 (110, 111). The optimal protein-ligand docking model, as determined from docking outcomes, was selected as the starting conformation for the simulation, with GAPDH used as a positive control (112, 113). The protein was modeled using the amber14sb force field, whereas the small molecule was represented with the Gaff2 force field. Using the TIP3P water model, the complex system was solvated, and a water box was formed with sodium ions to neutralize its charge. The Verlet and cg algorithms were used for elastic simulations, with Particle-Mesh Ewald (PME) handling electrostatic interactions. The system was subjected to energy minimization through the steepest descent method with a set step limit. The cutoff distances for Coulomb and van der Waals forces were set at 1.4 nm. Equilibration was achieved through both the constant volume (NVT) and constant pressure (NPT) ensembles, followed by a 100 ns MD simulation under standard temperature and pressure conditions. During the MD run, the LINCS algorithm was used to constrain hydrogen bonds with a two fs integration time step. PME calculations utilized a cutoff distance of 1.2 nm, while a 10 Å cutoff was set for non-bonded interactions. Temperature was kept at 300 K using the V-rescale thermostat, and pressure was stabilized at 1 bar with the Berendsen barostat. A 30 ps equilibration period was conducted under both NVT and NPT conditions at 300 K, preceding the 100 ns MD simulation of the protein-ligand complex. Local conformational shifts during the simulation were assessed using the root mean square fluctuation (RMSF) with a threshold of 0.2. The radius of gyration (Rg) was used to evaluate the structural compactness of the system, while RMSF offered insights into specific site fluctuations throughout the simulation.

### 2.5 Expression landscape analysis of DVL1 in gastrointestinal tumors

Recognizing the close association between gastrointestinal tumors and sepsis, this study performed a comprehensive analysis of DVL1 expression in various gastrointestinal cancers (COAD, ESCA, READ, and STAD) by comparing its expression in tumor and adjacent normal tissues to elucidate its role in tumor development (114, 115). Data from the TCGA and GTEx databases were integrated to investigate disparities in DVL1 expression between healthy individuals and cancer patients. The ability of DVL1 levels to distinguish between cancerous and healthy tissues was assessed using the pROC package, which included calculating the 95% confidence interval, the area under the curve (AUC), and creating ROC curves. Additionally, expression patterns of DVL1 in various cell subpopulations were analyzed using single-cell datasets associated with gastrointestinal tumors.

For methylation analysis, emphasis was placed on the TSS1500, TSS200, 1st Exon, and 5’ UTR regions, using Spearman correlation analysis to examine the relationship between methylation status and gene expression—particularly appropriate for analyzing correlations in non-normally distributed data. Copy number variation (CNV) analysis was carried out on 451 samples using the GISTIC scoring method, and the results were presented through bar charts. Chromosomal alterations were quantified, with indicators defined from C1 to C5. To explore expression differences among gene subgroups, ANOVA and TukeyHSD were employed for multiple comparisons.

Pathway activity was evaluated using the GSVA package with four parameters—z-score, GSVA, ssGSEA, and PLAGE—standardizing the results to Z-Score values. Differences in expression between tumor and normal tissues were tested using the Wilcoxon Rank Sum Test and visualized through boxplots using the ggplot2 package. The pan-cancer mutation landscape of the DVL1 gene was illustrated using the plotmafSummary function from the maftools package. Additionally, immune infiltration data from TCGA samples were retrieved from the TIMER 2.0 database to evaluate the presence of different immune cell types in the tumor microenvironment and their correlation with DVL1 expression. Correlations between immune cell abundance and gene expression were clearly illustrated using bar-scatter plots, showing correlation coefficients.

### 2.6 Spatial transcriptomic analysis of core genes at the single-cell level

In this paper, gene expression data obtained from the TISCH database for rectal cancer at the single-cell level up to October 2023 were analysed (116, 117). Heatmap of Gene Expression Patterns at the Single Cell Level in Different Cancer Types In order to detect and preserve gene expression patterns in different types of cancers, hierarchical clustering was performed using Euclidean distance and Ward’s minimum variance method. Due to the use of UMAP (Uniform Mobility Approximation and Projection) for high-dimensional data exploration, the original data structure was preserved as part of an algorithm designed specifically for non-linear data. Using UMAP to elucidate biological differences in gene expression in our cohort. The Kruskal-Wallis rank sum test was used to determine differences in gene expression between cell types. The Wilcoxon rank sum test is a non-parametric test used to determine if there is a significant difference between two independent groups. It does not assume that the data follow a normal distribution. The AUCell score, which quantifies the variability of pathway activity in single cells, was also used, as well as UMAP for visualisation. This approach provides a comprehensive view of the distribution of pathway activity and helps to identify potential biological differences.

### 2.7 Cell culture

RAW 264.7 Mouse macrophages (ATCC, Rockville, USA) were cultured in DMEM medium containing 10% heat-inactivated foetal bovine serum (FBS), 100 U/mL penicillin, and 100 μg/mL streptomycin at 37°C under 5% CO_2_. Digoxin and general HPLC reagents were purchased from Sigma (St. Louis, MO, USA). Cell culture media and supplements were provided by Invitrogen (Carlsbad, USA). THP-1 human monocytes (ATCC, Rockville, USA) were cultured in RPMI 1640 medium, which also contained 10% FBS, 100 U/mL penicillin, and 100 μg/mL streptomycin, and incubated under the same conditions at 37°C and 5% CO_2_ conditions. To induce differentiation into macrophages, THP-1 monocytes were exposed to PMA (100 ng/mL) for 5 days. To investigate the effect of Digoxin on P-glycoprotein (P-gp) activity in macrophages, RAW 264.7 cells were treated with 0.2 μM Digoxin for 4 hours (118, 119). Digoxin concentrations used in the treatments included 0, 0.025 mM (low), 0.05 mM (medium), and 0.1 mM (high).

Human colorectal cancer cell lines, including HCT116, SW480, CX-1, SW620, LoVo, COLO 205, LS-174T, and the normal colonic mucosa cell line FHC, were purchased from the American Typical Culture Collection (ATCC, Manassas, VA, USA). HCT116 cells were cultured in DMEM/F12 medium supplemented with 10% fetal bovine serum (FBS). SW480, SW620, and LoVo cells were maintained in DMEM containing 10% FBS. CX-1 and COLO 205 cells were grown in RPMI-1640 medium containing 10% FBS. In contrast, LS-174T cells were grown in Eagle Minimum Essential Medium (MEM) supplemented with 1% non-essential amino acids, 1 mM sodium pyruvate, and 10% FBS. Eagle Minimum Essential Medium (MEM) supplemented with 1% non-essential amino acids and 10% FBS. All cells were incubated at 37°C in a humidified environment with 5% CO_2_.

### 2.8 Statistical analysis

All statistical analyses were carried out with the help of GraphPad Prism 8.0 software. Descriptive statistics were used to summarise general data (120). For quantitative data, a t-test was used to compare means between two groups using an independent samples t-test. One-way analysis of variance (ANOVA) was used to assess differences in means between groups. P-values less than 0.05 were considered to indicate statistical significance.

## 3 Result

### 3.1 Core genes and pathways in sepsis-induced myocardial dysfunction: the role of DVL1

**Figure 1:**
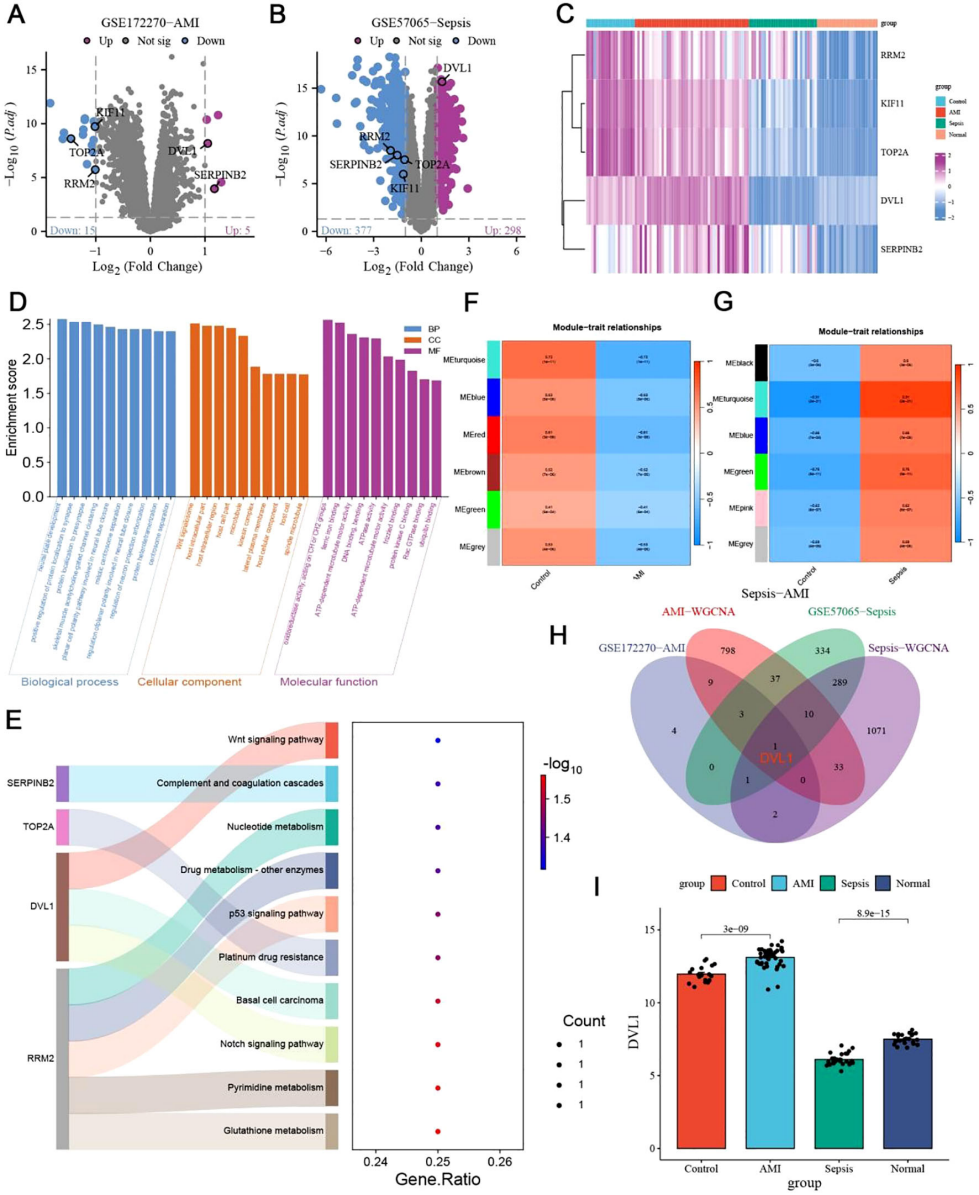
Identification of core genes in sepsis-induced myocardial dysfunction. **(A, B)** Volcano plots depicting differentially expressed genes (DEGs) in sepsis-Induced myocardial dysfunction. **(A)** DEGs from the GSE122720 dataset related to Acute Myocardial Infarction (AMI). Genes with significant upregulation (Log2 FC > 1, p < 0.05) are highlighted in red, while those significantly downregulated (Log2 FC < -1, p < 0.05) are shown in blue. Notable genes such as SERPINH1 and RRAD are labeled. **(B)** DEGs from the GSE57065 dataset related to sepsis. Significant upregulated and downregulated genes are indicated similarly, with DVL1 and SERPINH1 highlighted. **(C)** Heatmap of differentially expressed genes associated with Sepsis-Induced Myocardial Dysfunction. Hierarchical clustering of DEGs shows distinct expression patterns across different patient groups, with clustering performed on both gene expression profiles and patient samples. Key genes such as SERPINH1, TOP2A, and DVL1 are labeled, with expression levels indicated by the color gradient (from blue to pink representing low to high expression). **(D, E)** GO and KEGG pathway enrichment analysis of differentially expressed genes in Sepsis-Induced Myocardial Dysfunction. **(D)** GO enrichment analysis indicates significant biological processes, cellular components, and molecular functions associated with DEGs. **(E)** KEGG pathway enrichment analysis showing pathways such as Wnt signaling, complement and coagulation cascades, and nucleotide metabolism. The gene ratio indicates the proportion of DEGs involved in each pathway, with the color gradient representing the significance level [-log10(p-value)]. **(F, G)** Weighted Gene Co-expression Network Analysis (WGCNA) of Sepsis-Induced Myocardial Dysfunction. **(F)** Module-trait relationships identified in the AMI dataset, highlighting correlations between gene modules and clinical traits. **(G)** Module-trait relationships in the sepsis dataset, identifying key gene modules associated with disease severity. Color scale indicates the strength and direction of correlations. **(H)** Venn diagram illustrating the intersection of key genes identified across the datasets (GSE122720-AMI, GSE57065-Sepsis, AMI-WGCNA, Sepsis-WGCNA). This diagram highlights the core genes common to both conditions, emphasizing genes like DVL1 and SERPINH1 that are central to the disease process. **(I)** Relative expression analysis of the DVL1 gene across different patient groups (Control, AMI, Sepsis, Normal). The bar graph shows the mean ± standard deviation of DVL1 expression, with statistical significance denoted by p-values (e.g., p < 0.05). This analysis underscores the differential expression of DVL1 in Sepsis-Induced Myocardial Dysfunction, suggesting its potential role as a biomarker or therapeutic target.

The transcriptome analysis of Sepsis-Induced Myocardial Dysfunction (SIMD) across datasets, including GSE122720 for Acute Myocardial Infarction (AMI) and GSE57065 for sepsis, revealed significant differential expression patterns, with five core genes (KIF11, TOP2A, DVL1, RRM2, SERPINB2) being consistently differentially expressed across both conditions (**Figures 1A–E**). The hierarchical clustering of these genes highlighted distinct expression profiles, emphasizing their potential role in SIMD (**Figure 1C**). Subsequent Gene Ontology (GO) and KEGG pathway enrichment analyses identified key biological processes and pathways, such as the Wnt signaling pathway and complement cascades, which are implicated in the disease’s pathophysiology (**Figures 1D–E**). Further exploration using Weighted Gene Co-expression Network Analysis (WGCNA) pinpointed the MEturquoise module as significantly correlated with SIMD, containing numerous hub genes, including the core gene DVL1, which was consistently upregulated in SIMD (**Figures 1F–I**). The relative expression analysis of DVL1 across different patient groups further supported its potential as a biomarker, with significant upregulation observed in SIMD cases (**Figure 1I**). Supplementary analyses extended these findings to gastrointestinal tumors, where DVL1 was linked to poor prognosis and altered immune landscapes, reinforcing its role as a critical gene across multiple conditions (**Supplementary Figure 1**). Together, these results highlight the central role of DVL1 in SIMD and its broader implications in disease, positioning it as a promising target for future therapeutic strategies.

### 3.2 Molecular docking and dynamics simulation of DVL1 as a drug target

**Figure 2:**
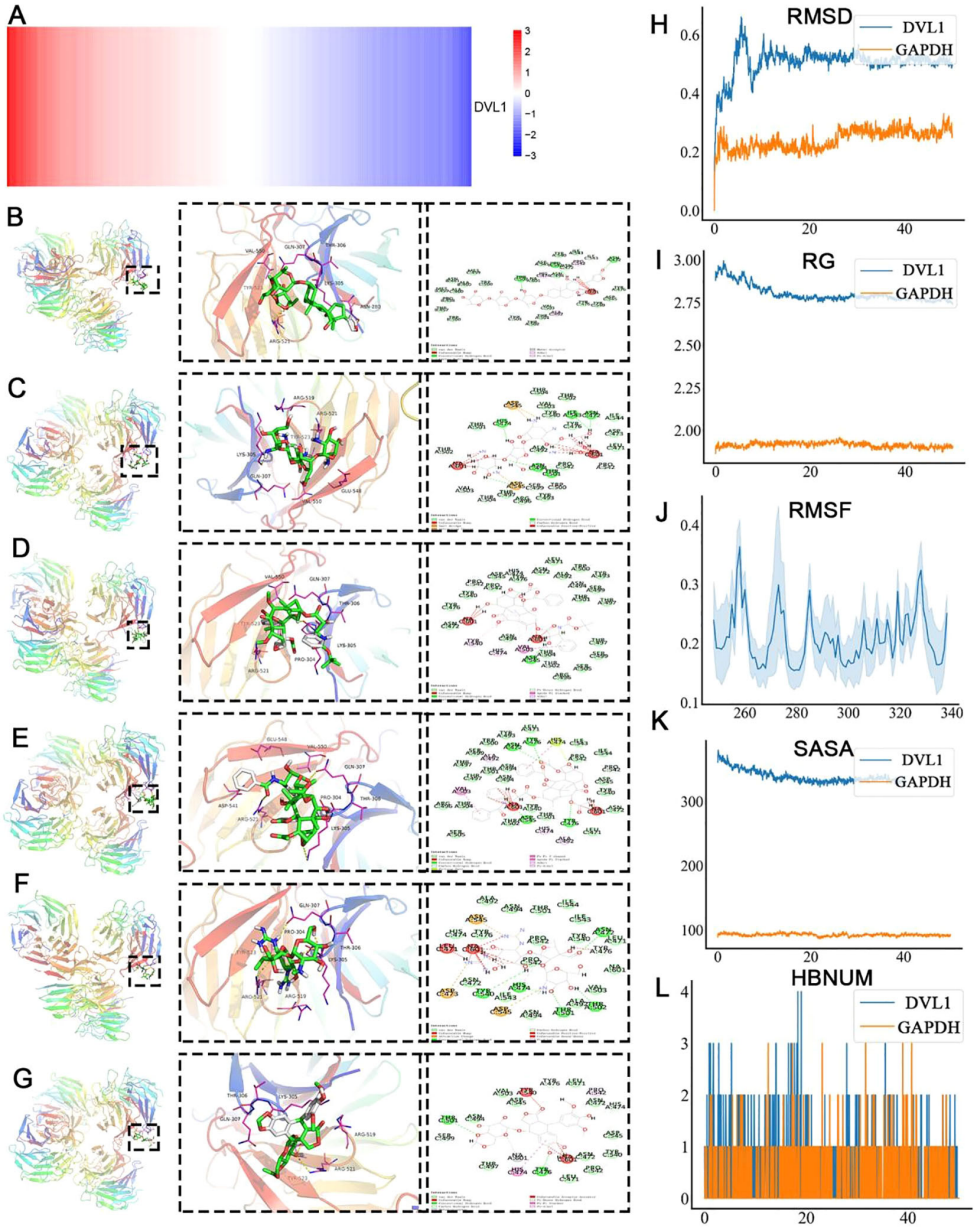
Molecular dynamics simulation and virtual screening of core protein DVL1. **(A)** Heat map representation of the virtual screening of core protein DVL1 against the FDA-approved drug library. The color gradient from red to blue represents the binding affinity, with red indicating high binding affinity and blue indicating low binding affinity. **(B-G)** Molecular docking models of the core protein DVL1 with selected ligands. Each panel shows the overall structure of DVL1 in a ribbon diagram (left), a zoomed-in view of the ligand-binding site with interacting residues highlighted (middle), and a 2D interaction diagram depicting the molecular interactions between DVL1 and the ligand (right). The models illustrate the different conformations of DVL1 when bound to various ligands, highlighting key interactions such as hydrogen bonds, hydrophobic contacts, and electrostatic interactions. **(H-L)** Molecular dynamics (MD) simulation analysis comparing DVL1 (blue) with the positive control protein GAPDH (orange). The analysis includes: **(H)** Root Mean Square Deviation (RMSD) analysis over the simulation time, showing the structural stability of DVL1 and GAPDH. **(I)** Radius of Gyration (RG) indicating the compactness of the protein structures. **(J)** Root Mean Square Fluctuation (RMSF) analysis, providing insight into the flexibility of specific residues within the protein structures. **(K)** Solvent Accessible Surface Area (SASA) analysis, representing the extent of exposure of the protein surface to the solvent. **(L)** Hydrogen Bond Number (HBNUM) analysis, illustrating the number of hydrogen bonds formed during the simulation, which correlates with protein stability.

The identification of DVL1 as a drug target was conducted through a combination of molecular docking and molecular dynamics (MD) simulations, revealing significant insights into its interactions with FDA-approved drugs. **Table 1** presents the binding affinity and docking scores of various compounds interacting with the DVL1 protein, as determined by molecular docking simulations using Autodock-Vina and Discovery Studio 2019 (**Table 1**). As shown in **Figure 2**, virtual screening highlighted small molecules with high binding affinity for DVL1, with docking scores visualized through a heat map (**Figure 2A**), where red represents strong binding affinity and blue represents weaker interactions. Among the top candidates, Digoxin was selected for further analysis due to its balanced docking score. Detailed molecular docking models (**Supplementary Figures 2B–G**) demonstrated the interaction of DVL1 with selected ligands, showcasing various conformations and key molecular interactions such as hydrogen bonds and hydrophobic contacts. MD simulations provided additional insights, with RMSD analysis (**Figure 2H**) showing fluctuations in the DVL1-Digoxin complex around 5 ns, stabilizing after 10 ns, indicating initial instability followed by equilibrium. The Radius of Gyration (RG) analysis (**Figure 2I**) revealed significant fluctuations in DVL1-Digoxin, suggesting transitions between unstable states, contrasting with the more stable GAPDH-Digoxin complex. RMSF analysis (**Figure 2J**) highlighted the flexibility of specific residues, with DVL1 showing considerable conformational changes. The Solvent Accessible Surface Area (SASA) analysis (**Figure 2K**) indicated a stable decrease in SASA for the DVL1-Digoxin complex, reflecting favorable binding and structural compactness. Finally, the Hydrogen Bond Number (HBNUM) analysis (**Figure 2L**) showed consistent hydrogen bond formation in both complexes, correlating with their stability. Overall, these findings underscore DVL1’s potential as a drug target, with Digoxin emerging as a promising ligand due to its strong binding and stability, as revealed through the comprehensive simulations.

**Table 1:** Molecular docking results of selected compounds with DVL1 protein (PDB ID: 6TTK) using autodock-vina and discovery studio 2019.

Protein (Binding Site)	Compound	Vina (kcal·mol^-1^)	RMSD	DS (LibDockScore)
**DVL1 (6TTK)**	**Digoxin**	-4.5	1.619	165.304
**DVL1 (6TTK)**	**Paromomycin**	-3.7	2.095	158.73
**DVL1 (6TTK)**	**Cabazitaxel**	-4.5	1.829	152.19
**DVL1 (6TTK)**	**Paclitaxel**	-4.6	1.516	151.868
**DVL1 (6TTK)**	**Streptomycin**	-4.6	2.614	148.925
**DVL1 (6TTK)**	**Toposar**	-5.2	0.452	147.71

This table presents the binding affinity and docking scores of various compounds interacting with the DVL1 protein, as determined by molecular docking simulations using Autodock-Vina and Discovery Studio 2019. The Vina score (expressed in kcal·mol^-^¹) reflects the binding affinity, where more negative values indicate stronger interactions between the compound and the protein. The RMSD (Root Mean Square Deviation) values provide insight into the stability and accuracy of the binding pose, with lower values indicating a more stable interaction. The DS (LibDockScore) from Discovery Studio 2019 represents the strength of interaction, with higher scores suggesting better binding affinity. The bold values in the table are the binding energies calculated by autodock - vina (Vina values, in kcal·mol- ¹), the root - mean - square deviations (RMSD), and the LibDockScore values calculated in Discovery Studio, which are used to measure the binding characteristics of compounds to the DVL1 protein.

### 3.3 Comprehensive analysis of DVL1 expression and mutation in gastrointestinal cancers

**Figure 3:**
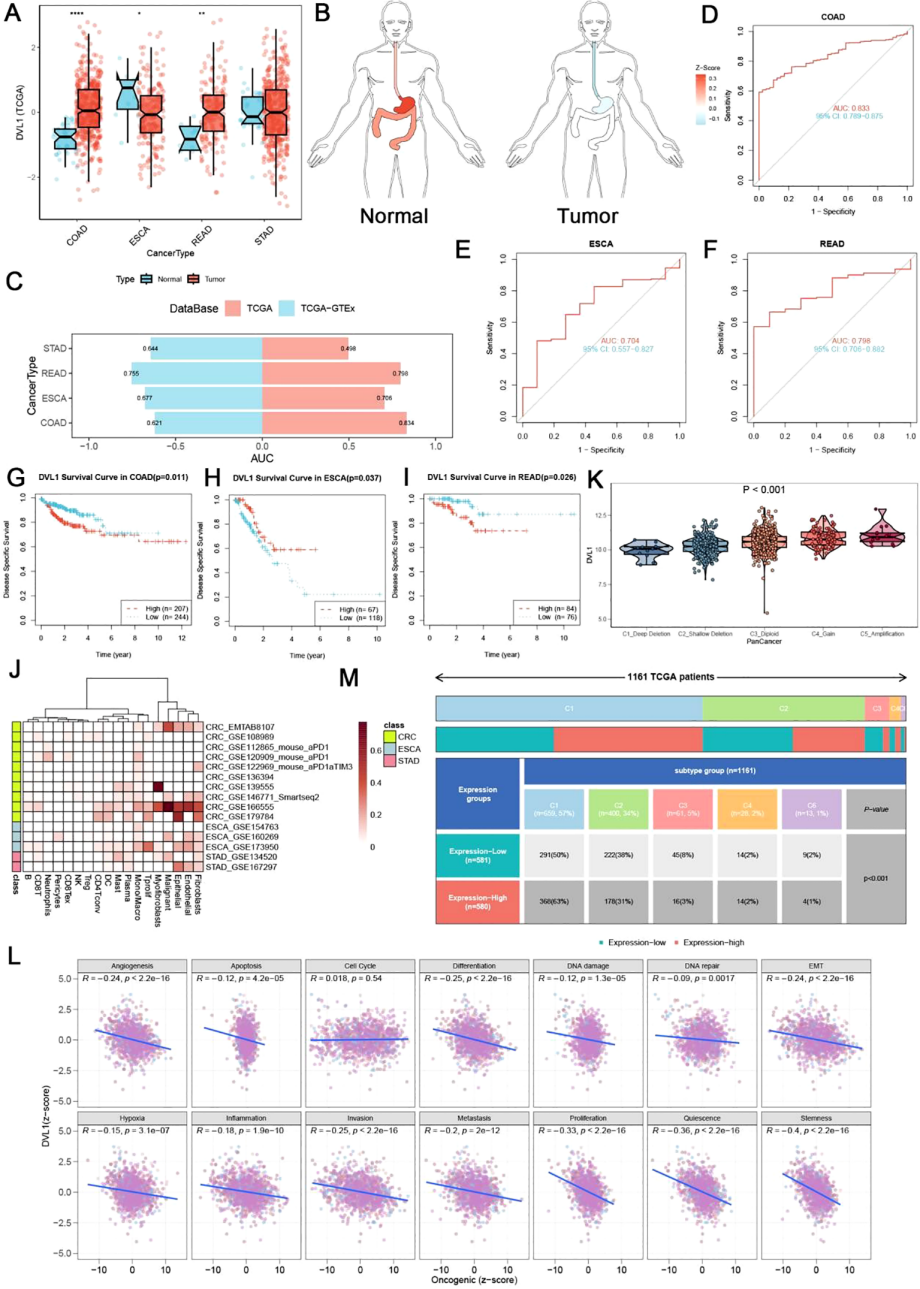
Landscape of DVL1 expression in gastrointestinal tumors. **(A, C)** DVL1 gene expression levels across four types of gastrointestinal tumors (COAD, STAD, ESCA, and READ) are depicted. Panel **(A)** shows a comparison between normal and tumor tissues based on data from the TCGA and GTEx databases, demonstrating differential expression patterns with statistical significance (indicated by p-values). Panel **(C)** provides a summary of the area under the curve (AUC) values for the receiver operating characteristic (ROC) analysis, reflecting the diagnostic potential of DVL1 in these cancers. **(B)** Illustration of DVL1 gene expression distribution across different organs in cancer patients, highlighting the variation in expression levels depending on the anatomical location of the tumor. **(D-F)** Receiver operating characteristic (ROC) curves for DVL1 gene in three types of gastrointestinal tumors (COAD, ESCA, and READ) are presented. The curves show the diagnostic accuracy of DVL1 expression, with each panel detailing the AUC values, sensitivity, and specificity metrics for each cancer type. **(G-I)** Kaplan-Meier survival curves analyzing the prognostic significance of DVL1 expression in three types of gastrointestinal tumors (COAD, ESCA, and READ). The survival analysis indicates the correlation between DVL1 expression levels and patient survival outcomes, with log-rank test p-values provided to denote statistical significance. **(J)** Heatmap showing DVL1 gene expression across different cell subgroups in four gastrointestinal tumors. This panel illustrates the heterogeneity in DVL1 expression among various cellular populations within the tumors. **(K)** Violin plot depicting the expression of DVL1 across different immune subtypes within gastrointestinal tumors. The plot demonstrates significant differences in DVL1 expression depending on the immune landscape of the tumor (p < 0.001). **(L)** Scatter plots examining the relationship between DVL1 expression and 14 different tumor phenotypes. Each plot includes regression lines and correlation coefficients, providing insight into the association between DVL1 expression and oncogenic pathways. **(M)** Summary of clinical data for 1181 TCGA patients with four types of gastrointestinal tumors, classified based on DVL1 expression levels. The panel provides an overview of clinical characteristics such as tumor stage, survival status, and molecular subtypes, highlighting the relevance of DVL1 expression in the clinical context. The symbols *, **, and **** represent statistical significance levels corresponding to p<0.05, p<0.01, and p<0.0001.

In this study, we investigated the role of DVL1 in gastrointestinal cancers, focusing on its expression pattern, diagnostic potential and prognostic significance. Analysis of DVL1 expression in four gastrointestinal tumors - COAD, STAD, ESCA and READ - showed significant overexpression in tumor tissues compared to normal tissues (**Figure 3A, C**). DVL1 gene expression levels varied in different organs of cancer patients, and the expression levels varied with the anatomical location of the tumor (**Figure 3B**). The ROC analysis demonstrated that DVL1 has strong diagnostic potential, with high AUC values in COAD, ESCA, and READ (**Supplementary Figures 3D–F**). Kaplan-Meier survival curves indicated that elevated DVL1 expression correlates with poorer survival outcomes in these cancers, suggesting its value as a prognostic marker (**Supplementary Figures 3G–I**). **Figures 3D–I** display the related analyses of DVL1 in gastrointestinal tumors, including ROC curves and survival curves for different tumor types. These figures intuitively demonstrate the important roles of DVL1 in diagnosis and prognosis. Further investigation revealed heterogeneity in DVL1 expression across different cellular populations within tumors (**Figure 3J**) and significant differences in expression across various immune subtypes (**Figure 3K**). Correlation analyses between DVL1 expression and oncogenic pathways highlighted its involvement in tumor biology (**Figure 3L**). A summary of clinical data from 1181 TCGA patients indicated that higher DVL1 expression is associated with advanced tumor stages and poorer outcomes (**Figure 3M**). In colorectal cancer, DVL1 showed a somatic mutation rate of 1.61%, with several mutation hotspots identified (**Supplementary Figure 2A**). Pan-cancer analysis confirmed DVL1 as one of the most mutated genes, emphasizing its potential role in carcinogenesis (**Supplementary Figure 2B**). Additionally, DVL1 expression varied significantly across different MSI subtypes, implicating it in MSI-driven tumorigenesis (**Supplementary Figure 2C**). Protein expression analysis using HPA data showed differential DVL1 expression in colorectal and stomach cancer tissues, supporting its involvement in cancer pathophysiology (**Supplementary Figure 2D**). These findings collectively suggest that DVL1 is a critical biomarker in gastrointestinal cancers, with significant implications for its use in diagnosis and prognosis.

### 3.4 Expression and prognostic significance of DVL1 in colorectal cancer

**Figure 4:**
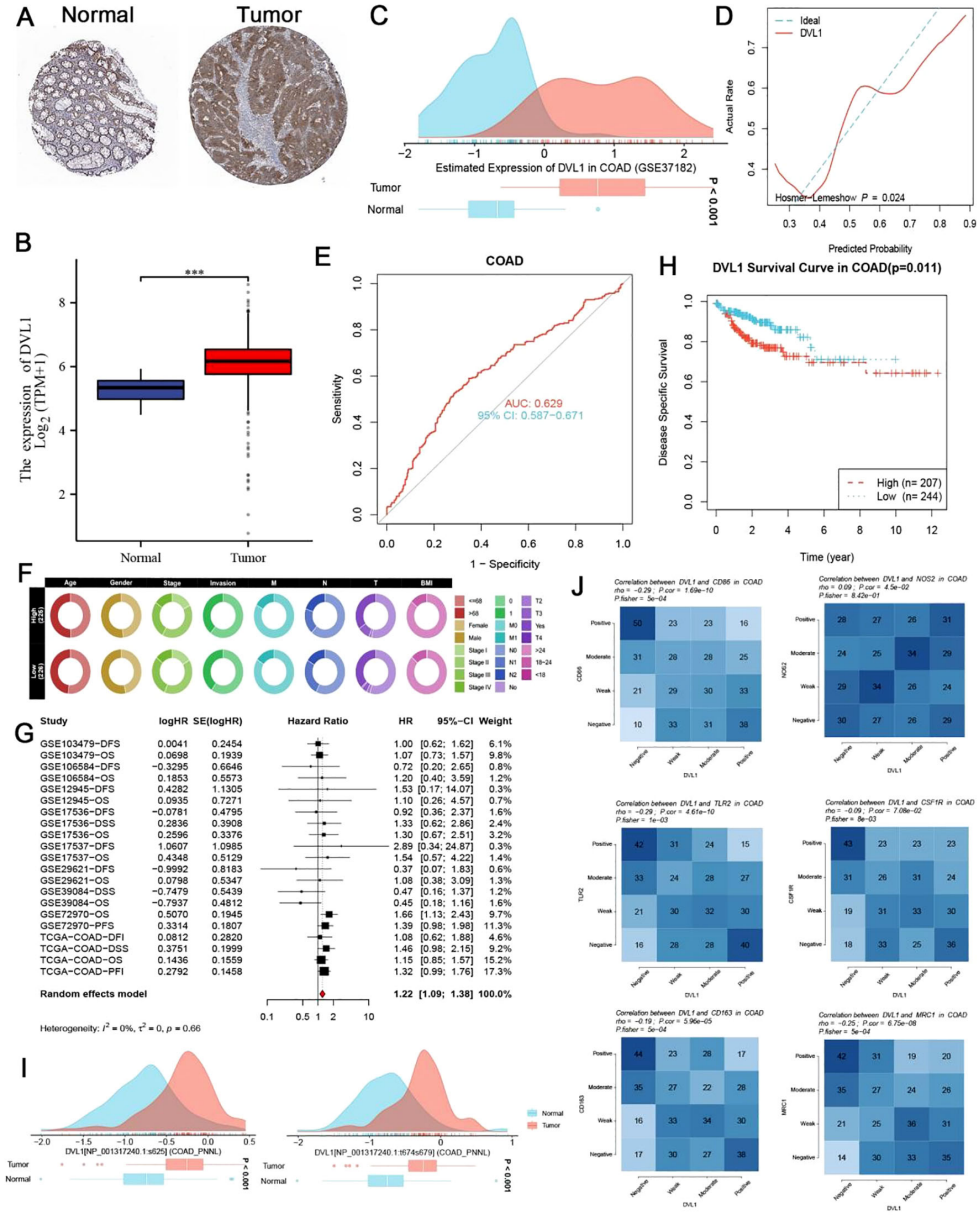
Expression and prognostic significance of DVL1 in colorectal cancer (COAD). **(A, B)** Immunohistochemical (IHC) staining for DVL1 in colorectal cancer tissues and adjacent normal tissues demonstrats increased expression of DVL1 in tumor tissues compared to normal tissues. Representative images from the study cohort are shown, with higher DVL1 expression observed in the tumor samples. Data were obtained using the Human Protein Atlas (HPA) database. **(C)** Distribution of DVL1 gene expression in colorectal cancer versus normal tissues, analyzed using the GSE37182 dataset. The data is presented as density plots, showing a significant upregulation of DVL1 in tumor tissues compared to normal tissues. **(D)** Receiver Operating Characteristic (ROC) curve assessing the diagnostic performance of DVL1 expression in distinguishing tumor tissues from normal tissues. The area under the curve (AUC) and the model’s discriminatory ability are shown, indicating a good diagnostic value for DVL1 expression in COAD. **(E)** ROC curve evaluating the prognostic performance of DVL1 expression in predicting outcomes in colorectal cancer patients. The AUC value and the 95% confidence interval (CI) are provided, highlighting the prognostic relevance of DVL1 expression in COAD. **(F)** Comparative analysis of clinical characteristics between high and low DVL1 expression groups in COAD patients. The circular heatmap visualizes the distribution of various clinical traits (e.g., age, gender, BMI, stage) between the two groups, illustrating significant associations with DVL1 expression. **(G)** Forest plot summarizing the univariate analysis of DVL1 expression across multiple datasets for COAD patients. Hazard ratios (HR) and 95% confidence intervals (CI) are depicted for each study, with a pooled HR calculated from the meta-analysis, indicating the overall prognostic impact of DVL1. The analysis shows a significant association between high DVL1 expression and poor prognosis. **(H)** Kaplan-Meier survival curve comparing overall survival between high and low DVL1 expression groups in COAD patients. The survival analysis shows a statistically significant difference (p = 0.011), with high DVL1 expression associated with worse survival outcomes. **(I)** Analysis of phosphorylation site changes in the DVL1 protein between normal and tumor tissues, indicating potential post-translational modifications that may contribute to altered function in colorectal cancer. The density plots depict the distribution of phosphorylation levels at specific sites, showing significant differences between normal and tumor groups. **(J)** Correlation heatmaps show the association between DVL1 expression and various immune markers in COAD, highlighting the positive and negative correlations with immune-related genes. The analysis provides insights into the potential role of DVL1 in modulating the immune microenvironment in COAD. The symbols *** represent statistical significance levels corresponding to p<0. 001.

Our study reveals that DVL1 is significantly overexpressed in colorectal cancer (COAD) tissues compared to adjacent normal tissues, as demonstrated by both immunohistochemical staining and RNA-seq analysis (**Figures 4A–C**). The ROC curves further confirm the diagnostic and prognostic value of DVL1, with AUC values indicating its potential to distinguish between tumor and normal tissues, and to predict patient outcomes (**Figures 4D, E**). High DVL1 expression correlates with poorer overall survival, as shown by Kaplan-Meier survival curves and a comparative analysis of clinical characteristics (**Figures 4F–H**). The observed changes in DVL1 phosphorylation sites between normal and tumor tissues suggest possible post-translational modifications contributing to its oncogenic role (**Figure 4I**). Thermal profiling data delineate significant covariations between DVL1 transcriptional activity and immunological biomarkers within COAD, establishing mechanistic insights into its regulatory potential within tumor-associated immune landscapes (**Figure 4J**). Additionally, the analysis of DVL1 expression across different tumor stages and its correlation with various COAD-related genes indicates a strong association with disease progression, although survival analysis did not show a significant difference between high and low-expression groups (**Supplementary Figures 3A–G**). Functional analyses, covering CRISPR-Cas9 screening as well as pathway enrichment studies, further highlighted the critical role of DVL1 in cancer biology, with significant enrichment in pathways associated with tumor progression (**Supplementary Figures 4A–I**). These findings suggest that DVL1 plays a crucial role in the development of COAD and could serve as a potential therapeutic target.

### 3.5 Analysis of DVL1 in colorectal cancer

In this research, we probed into the function of the DVL1 in COAD, concentrating on its expression patterns among tumor stages and its connection with key cancer-related pathways. **Supplementary Figure 4A** presents the distribution of DVL1 expression across four clinical stages of COAD (Stage I to IV). Statistical analysis shows that there are no remarkable differences in DVL1 expression levels among stages (P = 0.87). Likewise, a comparison between the early-stage (Stage I to II) and the late-stage (Stage III to IV) of COAD in **Supplementary Figure 4B** also reveals no significant difference (P = 0.995).To further explore the functional role of DVL1, we analyzed its dependency across various cancer types using CRISPR-Cas9 screening data from the DepMap database, as illustrated in **Figure 4C**. This analysis highlights variable DVL1 dependency across cancer cell lines, indicating its essential role in certain types of cancers. Next, we performed KEGG pathway enrichment analysis (**Supplementary Figure 4D**), which identified several cancer-related pathways associated with DVL1 expression, including the Wnt signaling pathway and pathways involved in cell cycle regulation. GSEA was conducted to assess hallmark gene sets, revealing significant enrichment in cellular processes related to proliferation, DNA repair, and apoptosis, particularly in the high DVL1 expression group, as shown in **Supplementary Figure 4E**. Additionally, **Supplementary Figure 4F** presents a LocusCompare analysis, which demonstrates specific genetic loci correlated with DVL1 expression. Finally, functional enrichment analysis for transcription factors associated with DVL1 expression was performed. The GO term analysis (**Supplementary Figure 4G**) highlights biological processes related to transcriptional regulation, while KEGG pathway analysis (**Supplementary Figure 4H**) indicates significant involvement in pathways such as p53 signaling and RNA polymerase activity. The Friends analysis in **Figure 4I** identifies key transcription factors, such as FOXM1 and NFKB2, which are strongly correlated with DVL1 expression and may contribute to its regulatory network in cancer. Collectively, these findings suggest that while DVL1 expression remains consistent across COAD stages, its dependency and functional interactions highlight its critical role in cancer biology, particularly in cell survival and proliferation pathways.

### 3.6 Comprehensive analysis of DVL1 in colorectal adenocarcinoma: gene interaction, immune landscape, and therapeutic implications

**Figure 5:**
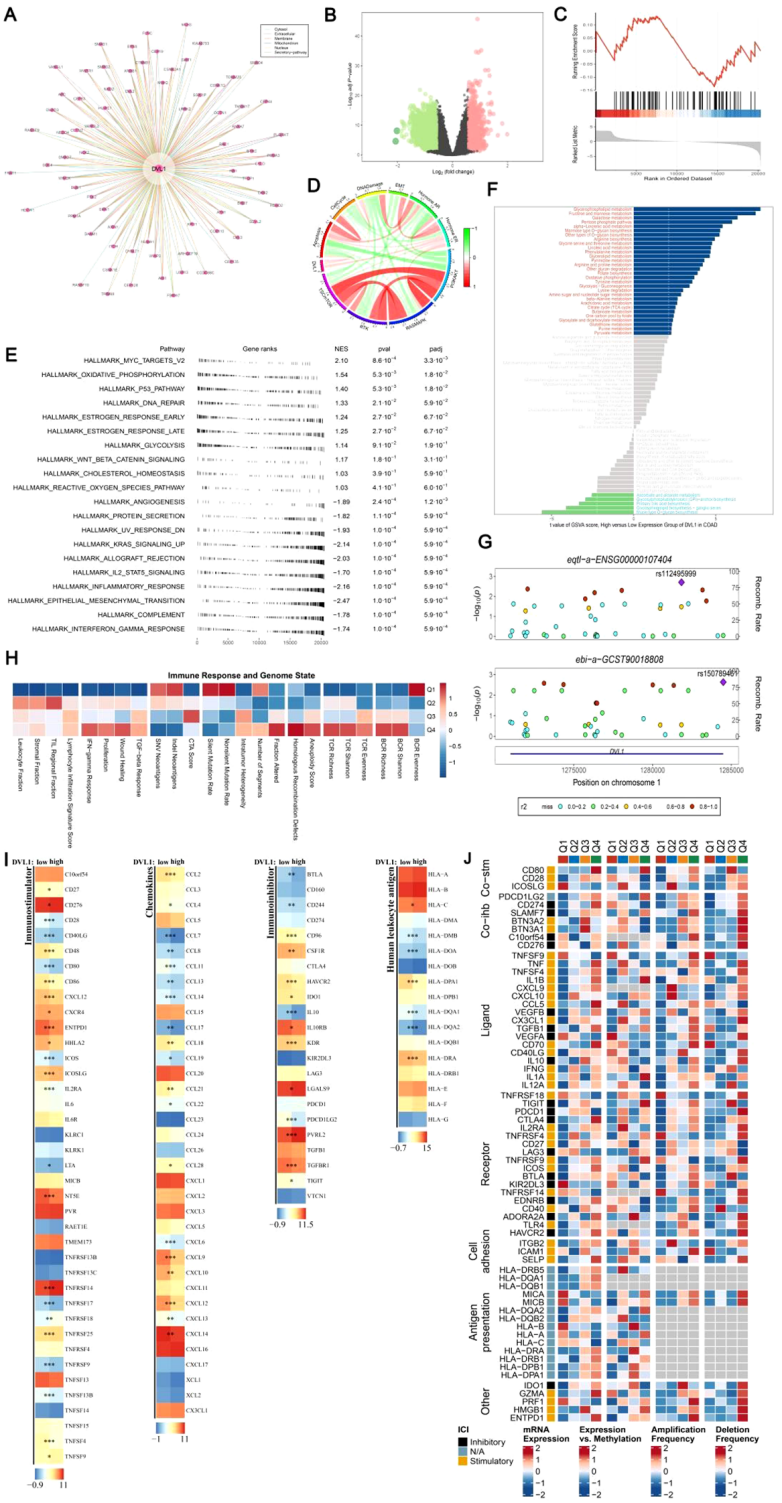
DVL1 gene enrichment analysis and associated immune landscape in COAD. **(A, D)** DVL1 gene interaction network: A network analysis illustrating the interactions of genes closely related to DVL1. Panel **(A)** shows a comprehensive gene-gene interaction network with DVL1 at the center, highlighting both direct and indirect interactions with associated genes. Panel **(D)** further details the connections and functional relationships among these genes using a circular plot. **(B)** Differential expression analysis of DVL1-associated genes: A volcano plot showing the differentially expressed genes associated with DVL1. Genes are categorized into upregulated (green), downregulated (red), and non-significant (black) groups based on log2 fold changes and statistical significance (p-value). **(C)** GSEA enrichment analysis of differentially expressed genes associated with DVL1: A line graph representing the Gene Set Enrichment Analysis (GSEA) results for DVL1-related differentially expressed genes, focusing on key gene sets that show significant enrichment or depletion. **(E)** Hallmark gene set enrichment analysis (GSEA): A dot plot visualizing the enrichment scores and significance levels of various hallmark gene sets associated with DVL1 expression. The pathways are ranked based on normalized enrichment score (NES) and statistical significance. **(F)** GSVA pathway enrichment scores comparing DVL1 high-expression versus low-expression groups: A bar chart depicting the difference in pathway activity scores between high and low DVL1 expression groups, identified using Gene Set Variation Analysis (GSVA). Pathways with significantly altered activity are color-coded based on their upregulation (blue) or downregulation (red) in high DVL1 expression groups. **(G)** Visualization analysis using gassocplot package: Scatter plots displaying the association between specific genetic variants and phenotypic traits related to DVL1, across various chromosomal locations. Points are color-coded according to their significance and categorized by variant type, with annotation of significant SNPs and genomic regions. **(H)** Immune response and genome state: A heatmap representing the correlation between DVL1 expression and various immune-related genes or pathways. Data points are color-coded to indicate the strength and direction of correlation, providing insights into the relationship between DVL1 and immune system activity. **(I)** Landscape of DVL1 in immunostimulator analysis: Heatmaps illustrating the association of DVL1 expression levels with various immunostimulatory genes across different sample sets. The analysis highlights significant correlations, with color intensities representing the degree of association. **(J)** Complex heatmap of immunomodulators in COAD: A detailed heatmap depicting the expression patterns, copy number variations, and mutation frequencies of key immunomodulatory genes in colorectal adenocarcinoma (COAD). The rows represent individual genes, and the columns represent different patient samples or conditions. The heatmap is annotated to show expression levels, amplification, deletion frequencies, and the presence of mutations, providing a comprehensive overview of the immunomodulatory landscape in relation to DVL1 expression in COAD. The symbols *, **, and *** represent statistical significance levels corresponding to p<0.05, p<0.01, and p<0. 001, respectively.

**Figure 6:**
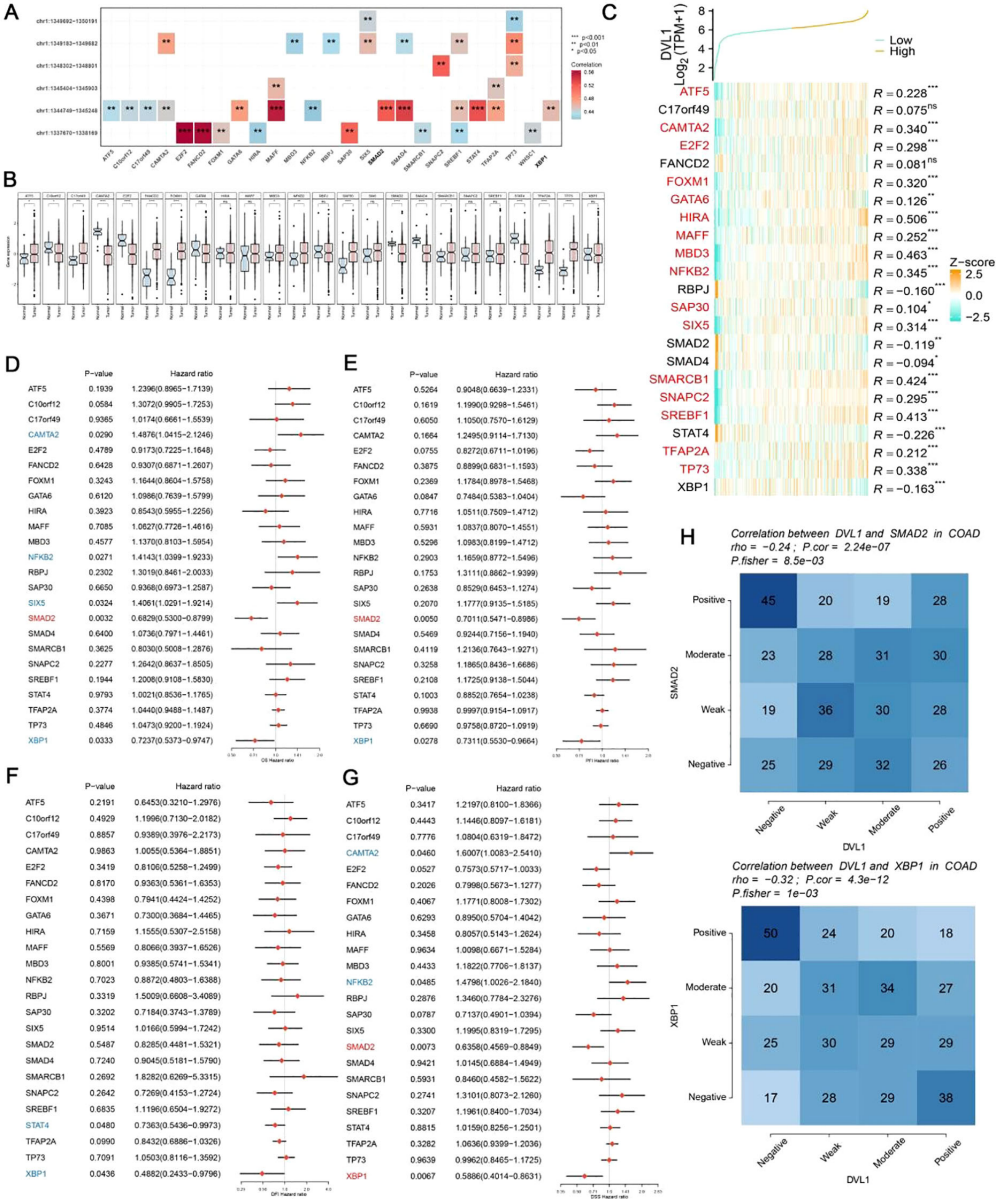
Analysis of upstream transcription factors of DVL1 gene. **(A)** Spearman correlation analysis between ATAC-Peak and transcription factors. This panel illustrates the Spearman correlation coefficients between ATAC-Peak signals and various transcription factors, providing insight into the potential regulatory relationships affecting DVL1 expression. The analysis highlights transcription factors with significant correlations, denoted by color-coded squares representing the strength and direction of correlation (positive in red, negative in blue). **(B)** Differential expression analysis of transcription factors associated with the DVL1 gene. Box plots display the expression levels of transcription factors across different sample groups, with statistical significance indicated for factors showing a differential expression. This analysis identifies transcription factors that are differentially regulated in association with DVL1, highlighting potential key regulators. **(C)** Friends analysis of the DVL1 gene to identify correlated transcription factors. A heatmap shows the correlation between DVL1 and selected transcription factors, identified through Friends analysis. Transcription factors with positive and negative correlations are listed alongside their correlation coefficients (R-values). The analysis helps to pinpoint transcription factors that may co-regulate with DVL1 or are part of the same regulatory network. **(D-G)** Forest plots screening prognostically relevant transcription factors through multi-gene analysis. These panels show hazard ratios and confidence intervals for multiple transcription factors in relation to overall survival in a cohort of cancer patients. The forest plots identify transcription factors significantly associated with prognosis, highlighting those with potential as biomarkers or therapeutic targets in conjunction with DVL1. **(H)** Correlation analysis between the DVL1 gene and transcription factors SMAD2 and XBP1. Heatmaps present the correlation strength between DVL1 and SMAD2/XBP1 across various samples, categorized into positive, moderate, weak, and negative correlations. The analysis provides a detailed view of the interaction between DVL1 and these specific transcription factors, offering insights into their potential collaborative roles in the biological processes studied. The symbols *, **, ***and ****represent statistical significance levels corresponding to p<0.05, p<0.01,p<0. 001 and p<0. 0001, respectively. ns, not significant.

The DVL1 gene plays a critical role in COAD, as demonstrated by a series of comprehensive analyses involving gene set enrichment, immune landscape evaluation, and upstream transcription factor studies. As illustrated in **Figure 5A**, a gene interaction network centered on DVL1 reveals significant associations with various genes, underscoring its involvement in essential cellular processes. Differential expression analysis (**Figure 5B**) further highlights the extensive alteration in gene expression associated with DVL1, implicating its pivotal role in tumor development. Gene set enrichment analysis (GSEA) and gene set variation analysis (GSVA) results (**Supplementary Figures 5C, E, F**) show significant pathway alterations between high and low DVL1 expression groups, particularly in hallmark gene sets, reinforcing DVL1’s influence on tumorigenesis. The immune response correlation (**Figure 5H**) and its association with immunostimulatory genes (**Figure 5I**) reveal a complex relationship between DVL1 expression and immune system activity, further supported by the immunomodulatory landscape in COAD (**Figure 5J**). Upstream transcription factors were analyzed to uncover potential regulatory mechanisms affecting DVL1 expression, with significant correlations observed between ATAC-Peak signals and specific transcription factors (**Figure 6A**). **Figure 6C** presents transcription factors associated with DVL1 identified via Friends analysis, offering critical insights for exploring the regulatory mechanisms of this signaling molecule. These transcription factors, highlighted in the differential expression analysis (**Figure 6B**) and prognostic forest plots (**Supplementary Figures 6D–G**), suggest potential targets for therapeutic intervention. The correlation of DVL1 with SMAD2 and XBP1 (**Figure 6H**) further suggests a collaborative role in COAD progression. In terms of therapeutic implications, DVL1’s role in predicting drug sensitivity and immunotherapy response is evidenced by the ROC-AUC analysis (**Supplementary Figure 5A**) and its significant correlation with drug sensitivity metrics (**Supplementary Figures 5B–D**). Notably, DVL1 expression correlated with increased sensitivity to the drug BI.2536 (**Supplementary Figures 5E, F**), suggesting that DVL1 could serve as a potential biomarker of drug response. Mutation analysis (**Supplementary Figures 6K–M**) provided insights into the mutational status of DVL1 and its impact on components of the tumor microenvironment (**Supplementary Figures 6N–P**), further cementing its relevance in colorectal cancer pathogenesis and treatment. Together, these findings highlight the importance of DVL1 as a key player in colorectal cancer, providing valuable insights for targeted therapy and prognostic assessment.

### 3.7 Comprehensive analysis of DVL1 expression in colorectal cancer using single-cell and spatial transcriptomics

**Figure 7:**
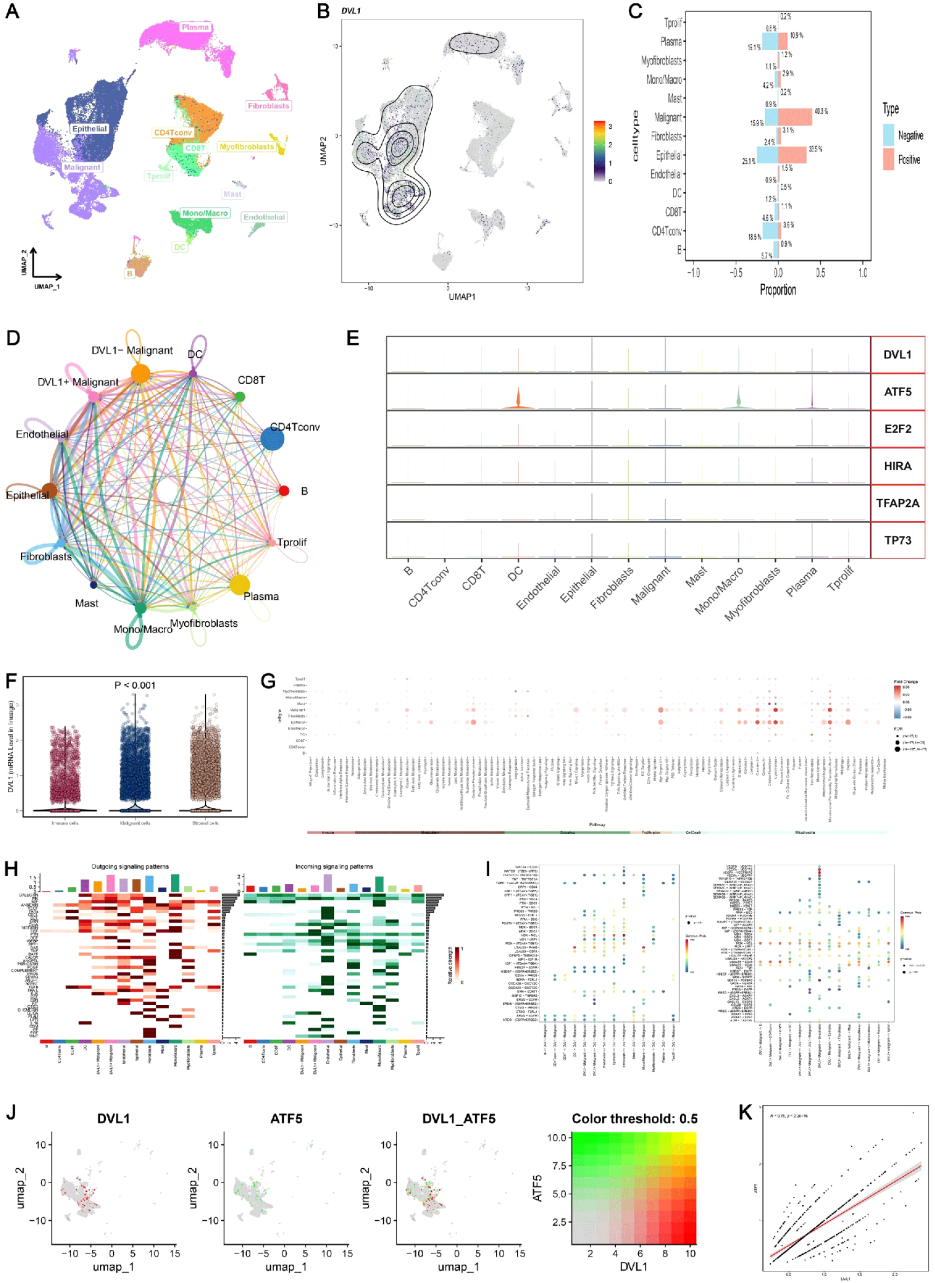
Single-cell sequencing analysis of DVL1 in colorectal cancer. **(A, B)** UMAP visualization of major cell lineages and DVL1 single-gene expression in single cells from colorectal cancer samples. **(A)** displays the distribution of the main cell lineages identified in the dataset, including T cells, B cells, epithelial cells, fibroblasts, myofibroblasts, mast cells, and others, with distinct clusters representing each lineage. **(B)** shows the UMAP plot highlighting the expression levels of the DVL1 gene across individual cells, with a gradient indicating varying expression levels. **(C)** Comparison of the proportions of different cell types between DVL1-positive and DVL1-negative groups. The bar graph presents the proportion of each cell type (e.g., T cells, B cells, fibroblasts) stratified by DVL1 expression status. The data suggest differential representation of cell types depending on DVL1 gene expression, with statistically significant differences noted. **(D)** Interaction network of different cell subsets in colorectal cancer. This network diagram illustrates the inferred interactions between various cell subsets within the tumor microenvironment, highlighting connections involving DVL1+ malignant cells and their interactions with other cell types such as CD8+ T cells, fibroblasts, and endothelial cells. The thickness of the lines corresponds to the strength or frequency of interactions. **(E)** Differential expression of DVL1 and upstream transcription factors across different cell types. The expression patterns of DVL1 and associated transcription factors such as ATF5, E2F2, HIRA, TFAP2A, and TP73 are shown across various cell types, including malignant cells, fibroblasts, and T cells. Each panel represents the distribution of expression levels across the cell types. **(F)** Variability in DVL1 expression across different tumor cell states. Box plots illustrate the differential expression of DVL1 among various tumor cell states, indicating statistically significant differences (P < 0.001). This comparison underscores the heterogeneity of DVL1 expression in distinct tumor microenvironments. **(G)** Pathway differences between DVL1-positive and DVL1-negative groups across different cell types. A dot plot shows the differential pathway activity scores between cells grouped by DVL1 expression status, across various cell types. Each dot represents a pathway, with size and color intensity reflecting the significance and magnitude of pathway activation differences. **(H, I)** Pathway enrichment differences between cell types. Heatmaps depict the enrichment of signaling pathways across different cell types in the tumor microenvironment. **(H)** displays outgoing signaling pathways, while **(I)** focuses on incoming signaling pathways. The data show distinct enrichment patterns, highlighting the unique roles of different cell types in signal transduction within the tumor context. **(J, K)** Correlation analysis of gene expression levels. **(J)** presents UMAP visualizations of the co-expression patterns of two specific genes, including DVL1 and ATF5, both individually and in combination. **(K)** shows a scatter plot demonstrating the correlation between the average expression levels of these two genes, with a color-coded threshold (0.5) indicating the strength of the correlation. This analysis reveals a significant positive correlation, suggesting potential regulatory interactions between DVL1 and ATF5.

**Figure 8:**
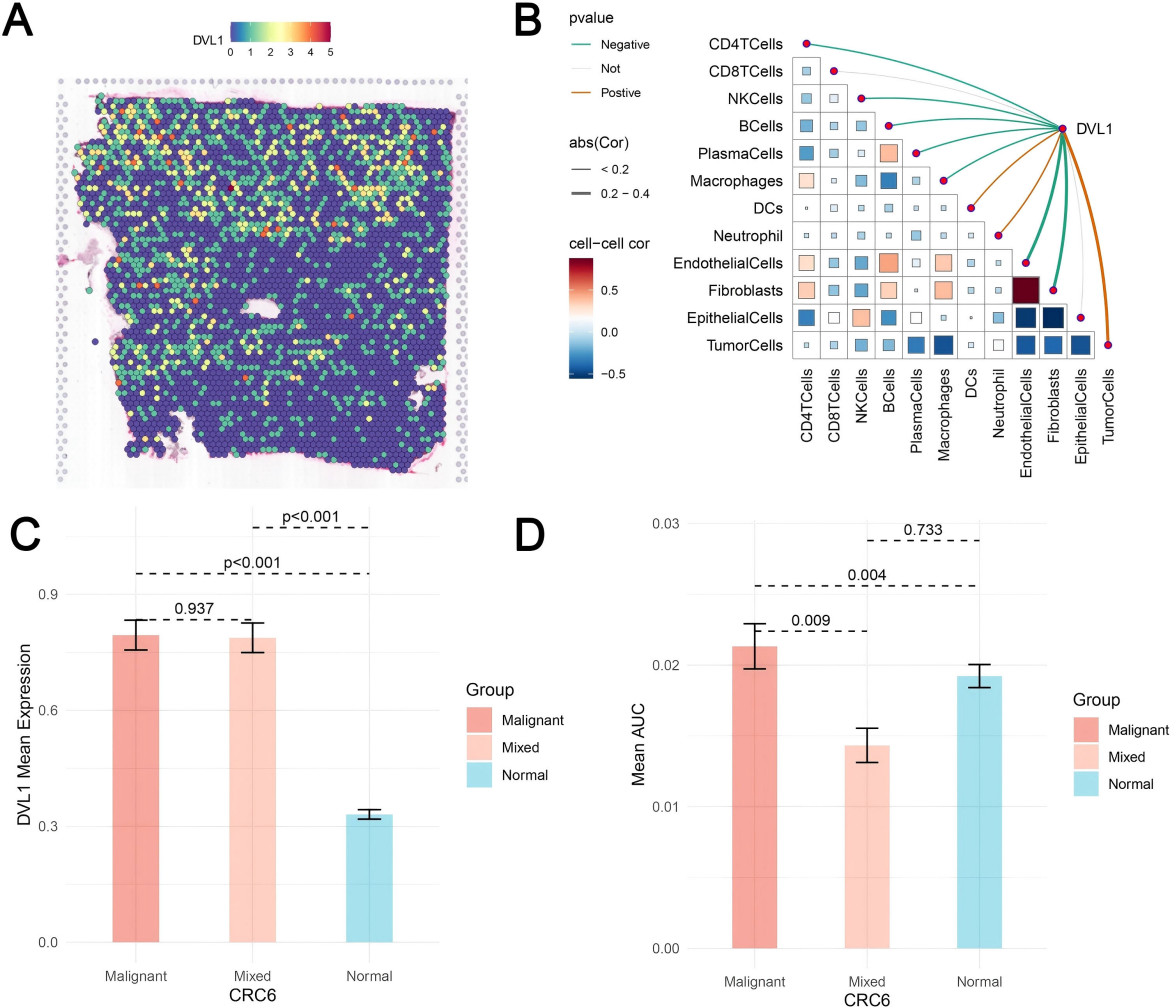
Spatial transcriptomics analysis of DVL1 in colorectal cancer. **(A)** Spatial distribution of DVL1 expression across the tissue sample. The heatmap illustrates expression levels, with higher intensities indicating elevated DVL1 expression. **(B)** Spearman correlation analysis between DVL1 expression and tumor microenvironment components. The correlation matrix represents the relationships between DVL1 and various cell types, with color gradients reflecting positive and negative correlations. Statistically significant correlations are highlighted. **(C)** Comparison of DVL1 mean expression levels among malignant, mixed, and normal tissue regions. Statistical significance is indicated by p-values. **(D)** Mean AUC values of a specific gene set across different tissue compositions, highlighting significant differences in tumor microenvironment interactions.

The comprehensive analysis of DVL1 expression in colorectal cancer, combining single-cell sequencing and spatial transcriptomics, reveals critical insights into the gene’s role within the tumor microenvironment. Through UMAP visualization of major cell lineages (**Figure 7A**), distinct clusters such as T cells, B cells, epithelial cells, and fibroblasts were identified, with **Figure 7B** highlighting varying levels of DVL1 expression across these populations. The comparison of cell type proportions between DVL1-positive and DVL1-negative groups (**Figure 7C**) underscores the differential representation of cell types, notably a higher presence of fibroblasts and T cells in DVL1-positive samples. Furthermore, the interaction network in **Figure 7D** demonstrates the significant role of DVL1+ malignant cells in coordinating cellular interactions, particularly with CD8+ T cells and fibroblasts. Differential expression analysis in **Figure 7E** highlights the association between DVL1 and transcription factors like ATF5 and E2F2 across various cell types. The variability in DVL1 expression across tumor cell states (**Figure 7F**) and pathway activity differences between DVL1-positive and DVL1-negative cells (**Figure 7G**) emphasize the gene’s influence on tumor heterogeneity and pathway activation. The pathway enrichment of different cell types in the tumor microenvironment (TME) revealed the differential activity of pro-inflammatory and immune regulation-related signaling pathways (e. g. Wnt/β -catenin, NF- κ B, TGF- β) in immune cells, fibroblasts and tumor cells (**Figures 7H, I**) Tumor-associated fibroblasts (CAF) showed high activity in TGF- β and ECM-related pathways, while Treg cells were enriched in IL-10-mediated anti-inflammatory pathways, suggesting unique roles for different cell types in TME regulation. Correlation analysis between DVL1 and ATF5 (**Figures 7J, K**) suggests a regulatory interaction, potentially impacting tumor progression. Spatial transcriptomics provides a refined visualization of DVL1 expression within tumor tissue, revealing its heterogeneous spatial distribution (**Figure 8A**). A robust correlation is noticed between the expression of DVL1 and key microenvironmental constituents (**Supplementary Figures 7A–D**). Spatial mapping further accentuates the enhanced expression of DVL1 in malignant areas, indicating its potential engagement in tumor progression and aggression (**Figures 8B–D**). Altogether, these discoveries emphasize the crucial role of DVL1 in coordinating the cellular and spatial dynamics of colorectal cancer, molding microenvironmental interactions and influencing tumor behavior.

### 3.8 DVL1 expression and its role in modulating cell proliferation and tumor progression

**Figure 9:**
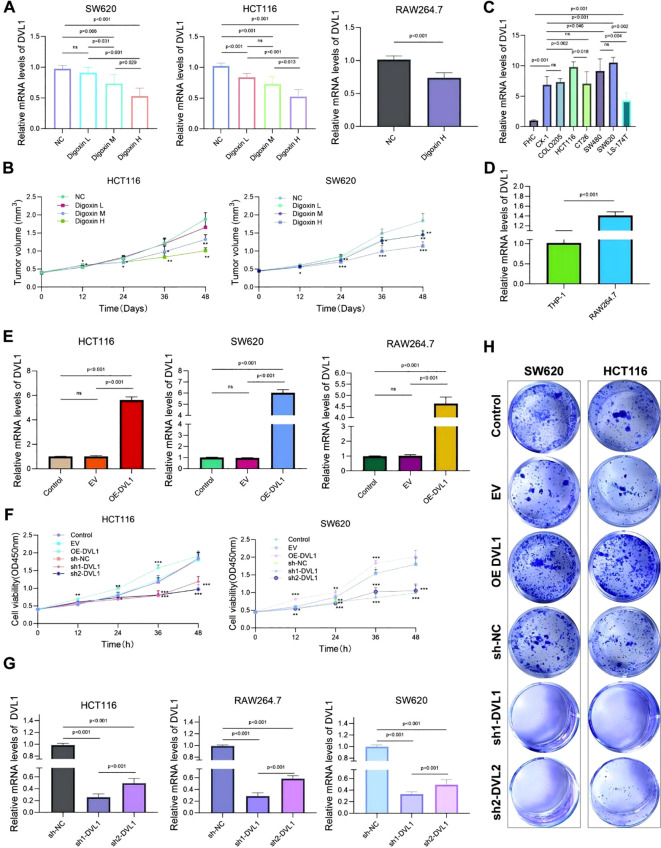
Analysis of DVL1 expression and its effects on cell proliferation and tumor progression across various cell lines. **(A)** Quantitative PCR Analysis: Relative mRNA levels of DVL1 in SW620, HCT116, and RAW264.7 cell lines following treatment with different concentrations of Digoxin (H, High; M, Medium; L, Low) were assessed using qPCR. The expression of DVL1 is significantly downregulated in cells treated with Digoxin, especially at high concentrations (p < 0.001). **(B)** CCK-8 Assay: Cell viability analysis of HCT116 and SW620 cells treated with different concentrations of Digoxin (H, M, L) over 48 hours using the CCK-8 assay. The results indicate a dose-dependent reduction in tumor volume in both cell lines, with Digoxin H showing the greatest inhibitory effect on proliferation (p < 0.05). **(C)** Inflammatory Cytokine Expression: Quantitative PCR was used to detect the mRNA expression levels of key inflammatory cytokines in multiple cell lines, including SW620, HCT116, and RAW264.7, treated with various conditions. Notable changes in cytokine expression are evident across different treatments (p < 0.001). **(D)** DVL1 Expression in THP-1 and RAW264.7 Cells: qPCR analysis of DVL1 mRNA levels in THP-1 and RAW264.7 cell lines, highlighting significant upregulation in RAW264.7 cells compared to THP-1 (p < 0.001). **(E)** DVL1 Expression in Various Cell Lines: qPCR results show the expression of DVL1 in SW620, HCT116, and RAW264.7 cells. The data indicate distinct differences in DVL1 expression levels among cell lines, with overexpression observed in specific groups (p < 0.001). **(F)** Impact of DVL1 on Cell Proliferation: The effect of DVL1 on cell proliferation in HCT116 and SW620 cells was evaluated using a CCK-8 assay. Results indicate that overexpression of DVL1 promotes cell proliferation, whereas knockdown significantly inhibits growth in both cell lines (p < 0.001). **(G)** DVL1 Expression Post-Knockdown: Quantitative PCR analysis of DVL1 expression in SW620, HCT116, and RAW264.7 cells following DVL1 knockdown. Results demonstrate a significant decrease in DVL1 mRNA levels across all tested cell lines after knockdown treatment (p < 0.001). **(H)** Plate Colony Formation Assay: Assessment of DVL1’s impact on colony-forming ability in SW620 and HCT116 cells through plate cloning experiments. Images depict colonies from control, EV (empty vector), DVL1 overexpression (OE-DVL1), and DVL1 knockdown groups (sh-DVL1). The data suggest that DVL1 overexpression enhances colony formation, while knockdown reduces it. The symbols *, **, and *** represent statistical significance levels corresponding to p<0.05, p<0.01, and p<0. 001, respectively. ns, not significant.

The study investigated the expression of DVL1 and its impact on cell proliferation and tumor progression across various cell lines, including SW620, HCT116, and RAW264.7. Quantitative PCR analysis, as shown in **Figure 9A**, revealed that DVL1 mRNA levels were significantly downregulated in response to different concentrations of Digoxin, particularly at high concentrations (p < 0.001). Among the tested cell lines, HCT116 and SW620 exhibited the most substantial reductions in DVL1 expression, suggesting that Digoxin effectively suppresses DVL1 expression. Additionally, **Figure 9D** highlights a significant upregulation of DVL1 in RAW264.7 cells compared to THP-1 cells (p < 0.001). To assess the functional role of DVL1, a CCK-8 assay was performed, revealing a dose-dependent decrease in cell viability in HCT116 and SW620 cells treated with Digoxin, with the greatest inhibition observed at high concentrations, as illustrated in **Figure 9B** (p < 0.05). This suggests that Digoxin-mediated DVL1 downregulation contributes to reduced tumor cell proliferation. Moreover, the expression of inflammatory cytokines was measured using qPCR across different treatments, showing significant changes in SW620, HCT116, and RAW264.7 cells (**Figure 9C**, p < 0.001), indicating a role of DVL1 in modulating inflammatory responses. Further analysis involved DVL1 overexpression and knockdown models, where **Figure 9E** demonstrated that overexpression significantly promoted cell proliferation, whereas knockdown markedly inhibited growth (p < 0.001). The plate colony formation assay results, depicted in **Figure 9H**, supported these findings, showing enhanced colony formation with DVL1 overexpression and a reduction with knockdown. CCK-8 proliferation assays demonstrated that DVL1 overexpression enhances cellular growth kinetics in both HCT116 and SW620 colorectal cancer models, whereas genetic silencing of DVL1 exerted potent growth-suppressive effects (**Figure 9F**). Finally, **Figure 9G** showed a substantial decrease in DVL1 mRNA levels in SW620, HCT116, and RAW264.7 cells following DVL1 knockdown (p < 0.001). Collectively, these results suggest that DVL1 plays a critical role in promoting cell proliferation and tumor progression, and that down-regulation of its expression by pharmacological agents or gene knockdown significantly inhibits these processes.

### 3.9 The effects of digoxin and DVL1 overexpression on inflammatory responses, cell viability, migration, and protein expression in cancer cells

**Figure 10:**
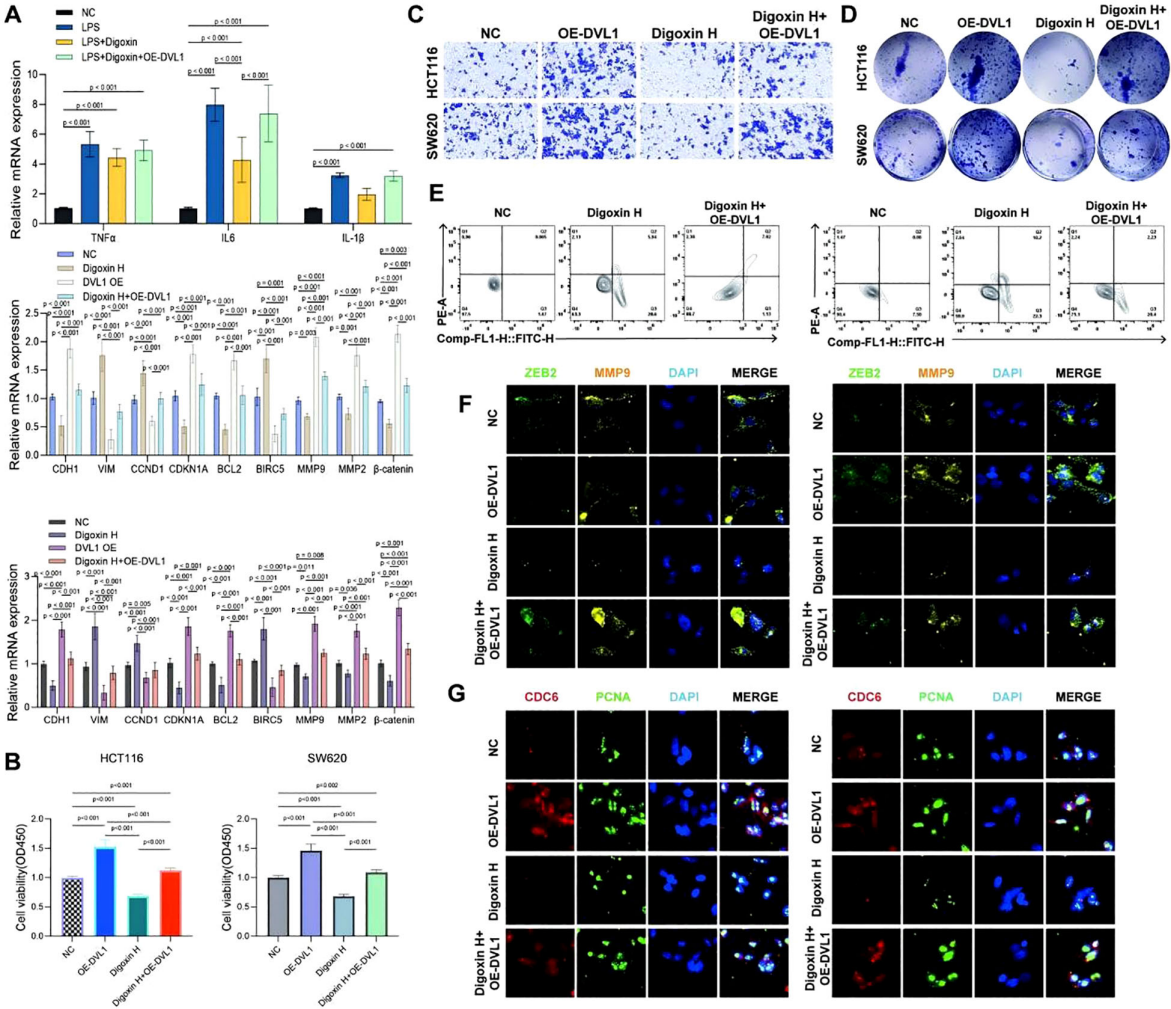
The effects of digoxin and DVL1 overexpression on inflammatory cytokine expression, cell viability, migration, proliferation, apoptosis, and key protein expression in SW620 and HCT116 cell lines. **(A)** Relative mRNA expression levels of inflammatory cytokines (TNFα, IL6, IL1β) and key EMT markers (CDH1, VIM, CDK1A, CDKN1, BCL2, BIRC5, MMP9, MMP2, β-catenin) were assessed in SW620 and HCT116 cell lines under different treatment conditions: Control group (NC), LPS, Digoxin, OE-DVL1, and the combination of LPS and Digoxin with OE-DVL1 overexpression. Data are presented as mean ± SD, with statistical significance indicated by p-values. **(B)** The effects of varying concentrations of Digoxin (High, Medium, Low) and OE-DVL1 on the viability of SW620 and HCT116 cells were measured using the CCK-8 assay. Significant decreases in viability were observed in cells treated with Digoxin H compared to controls, with further reductions upon OE-DVL1 overexpression. **(C)** The migratory capacity of SW620 and HCT116 cells was assessed using Transwell chambers. Cells treated with Digoxin H and those overexpressing DVL1 showed reduced migration compared to the control group, highlighting the role of Digoxin and DVL1 in inhibiting cell migration. **(D)** Proliferative ability was evaluated by plating SW620 and HCT116 cells. Colony formation was significantly reduced in the Digoxin H treatment group, with a further decrease in the combination of Digoxin H and OE-DVL1, indicating the suppressive effect of these treatments on cell proliferation. **(E)** Apoptosis was measured using flow cytometry in SW620 and HCT116 cells under various treatments. Increased levels of apoptosis were observed in cells treated with Digoxin H, especially when combined with OE-DVL1, compared to untreated controls. **(F, G)** Immunofluorescence staining of ZEB2, MMP9, CDC6, and PCNA: The expression of EMT marker ZEB2 and matrix metalloproteinase MMP9, as well as the cell cycle regulators CDC6 and PCNA, were visualized by immunofluorescence in SW620 and HCT116 cells. Cells treated with Digoxin H and those overexpressing OE-DVL1 displayed significant changes in these protein expressions, indicating alterations in EMT and cell cycle progression.

**Figure 11:**
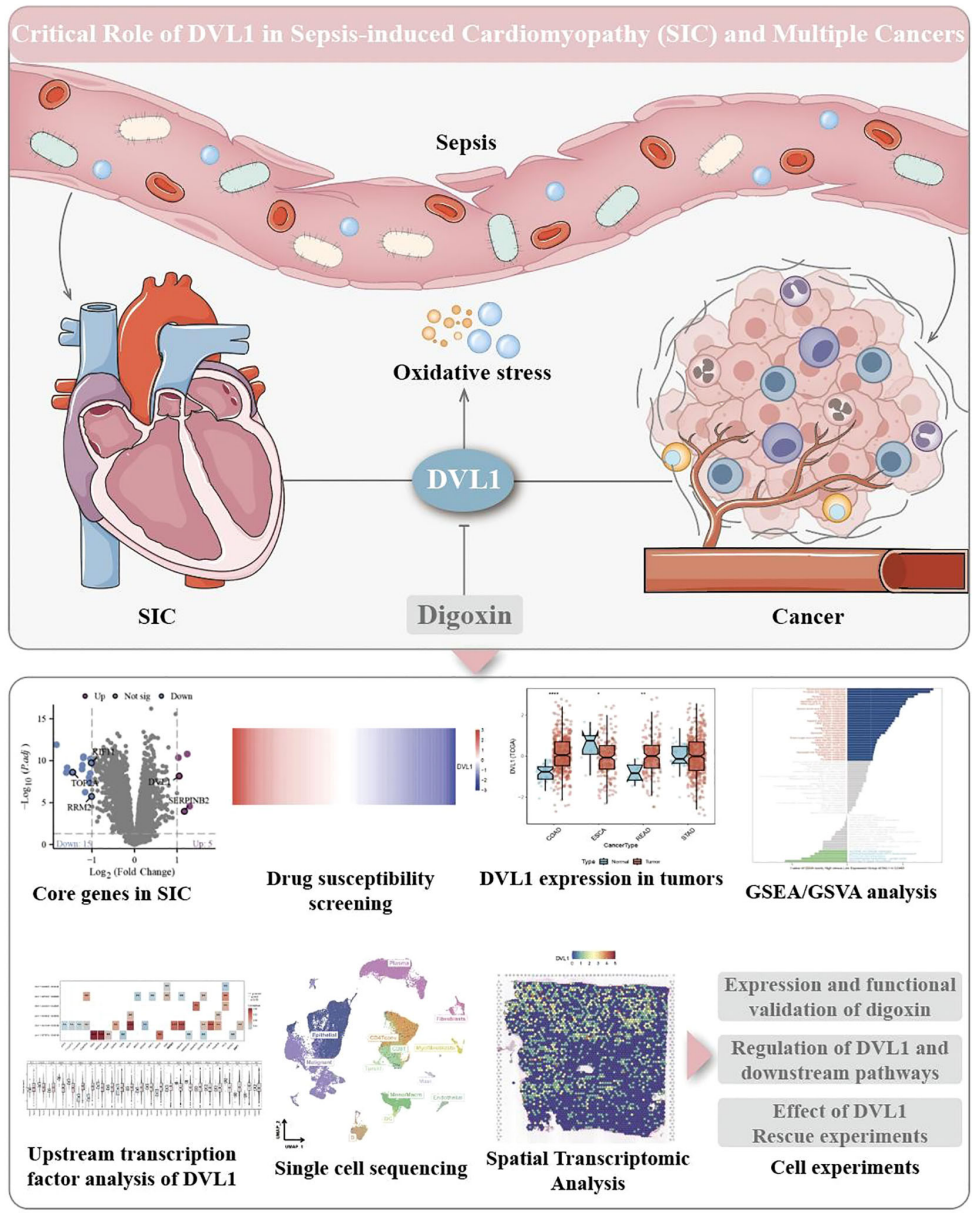
Critical role of DVL1 in sepsis-induced cardiomyopathy (SIC) and multiple cancers. This figure illustrates the central role of DVL1 in the progression of sepsis-induced cardiomyopathy and its association with various cancers, emphasizing the role of oxidative stress as a key mediator. The upper part of the image depicts sepsis-induced systemic inflammation leading to increased oxidative stress, which impacts the cardiovascular system, resulting in SIC. The illustration also highlights DVL1’s involvement in cancer development and progression through its effects on tumor microenvironments. Positioned centrally, DVL1 acts as a crucial node that links oxidative stress responses to both cardiac dysfunction and oncogenic processes. Digoxin is indicated as a potential therapeutic agent that targets DVL1, offering a promising approach for modulating DVL1 activity and its downstream pathways. The lower section of the figure provides an overview of various experimental analyses related to DVL1’s function. It includes differential expression analysis of core genes in SIC, highlighting significant alterations in gene expression (e.g., DVL1) through a volcano plot. Drug susceptibility screening results are presented in a heatmap, identifying the responsiveness of SIC-associated cells to potential therapeutic agents. Additionally, the figure shows DVL1 expression across different tumor types through box plots, revealing its dysregulation in multiple cancers. Gene set enrichment analysis (GSEA) and gene set variation analysis (GSVA) further demonstrate DVL1’s involvement in critical signaling pathways. Transcription factor analysis and single-cell sequencing provide insights into the regulatory mechanisms and cellular heterogeneity associated with DVL1 expression. Spatial transcriptomic analysis maps the spatial distribution of DVL1 in tissue samples, while cell-based experiments validate the functional impact of DVL1, including the effects of Digoxin treatment and DVL1 knockdown on downstream signaling and cell viability.

This study investigated the impact of Digoxin and OE-DVL1 on various cellular processes in SW620 and HCT116 cell lines, focusing on inflammatory cytokine expression, EMT markers, cell viability, migration, proliferation, apoptosis, and protein expression. qRT-PCR analysis showed that Digoxin and OE-DVL1 significantly reduced the expression of pro-inflammatory cytokines TNFα, IL6, and IL1β compared to the LPS-treated group, suggesting an anti-inflammatory effect (**Figure 10A**). The expression of key EMT markers such as CDH1, VIM, and MMP9 was modulated by the combination of Digoxin and OE-DVL1, suggesting its role in inhibiting EMT-related changes (**Figure 10A**). CCK-8 demonstrated that Digoxin, particularly at high doses, significantly reduced the viability of SW620 and HCT116 cells, with further decreases when combined with OE-DVL1, highlighting their combined inhibitory effect on cell proliferation (**Figure 10B**). Transwell migration assays confirmed that both Digoxin and OE-DVL1 significantly reduced cell migration, further supporting their role in inhibiting metastatic potential (**Figure 10C**). Colony formation assays showed a marked decrease in the number of colonies formed in cells treated with Digoxin, with an additional reduction observed when combined with OE-DVL1, suggesting enhanced anti-proliferative effects (**Figure 10D**). Flow cytometry analysis indicated increased apoptosis levels in cells treated with Digoxin, particularly when combined with OE-DVL1, highlighting the pro-apoptotic effects of these treatments (**Figure 10E**). Immunofluorescence staining revealed decreased expression of EMT markers ZEB2 and MMP9, as well as cell cycle regulators CDC6 and PCNA, indicating alterations in EMT status and cell cycle progression following treatment (**Figures 10F, G**). Furthermore, **Figure 11** illustrates the broader role of DVL1 in SICand multiple cancers, with oxidative stress identified as a critical mediator linking DVL1 to these conditions. Digoxin emerges as a potential therapeutic agent targeting DVL1, modulating its activity and downstream signaling pathways. The combined results from transcriptomic analyses, drug susceptibility screening, and spatial transcriptomics provide a comprehensive view of DVL1’s involvement in both SIC and cancer, positioning Digoxin as a promising therapeutic strategy for regulating these pathways.

## 4 Discussion

SIC is a serious complication in critically ill cancer patients and is closely associated with heart failure and high mortality (2, 121). TME-driven immune imbalance may exacerbate the development and progression of SIC (122, 123). The interaction mechanisms between tumor-associated inflammation, dysfunction of VSMC, and myocardial injury are still poorly elucidated (56, 124). Therefore, it is crucial to investigate the pathogenesis. In this study, the role of DVL1 in SIC was investigated by integrating transcriptome analysis, WGCNA, molecular docking and drug screening, and explored the possibility of DVL1 as a potential therapeutic target for SIC.

This study revealed the critical role of DVL1 gene in SIC in cancer patients (2). Multiomic analysis indicates that DVL1 is significantly upregulated in SIC and various gastrointestinal cancers, and is closely associated with the occurrence of poor prognosis and enhanced inflammatory response (115, 125). These findings not only highlight the role of DVL1 in the progression of SIC, but also elucidate its underlying mechanism in immune regulation within the tumor microenvironment. Furthermore, these insights have important implications for understanding the differentiated response patterns during patient immunotherapy (63, 126). We used a variety of experimental methods to study the function of DVL1 in SIC and selected FDA-approved drugs such as Digoxin and paromomycin as potential inhibitors of DVL1. Among them, Digoxin reduces the level of sepsis-induced oxidative stress by targeting DVL1, thereby improving the survival rate of cardiomyocytes. The results of this study provide a new direction for pharmacological intervention in SIC. Approved drugs (repurposed drugs) may be used to improve clinical outcomes in patients with SIC. Especially in cancer patients, the impact of SIC on cardiac function may be more severe, and thus DVL1-targeted therapies may be an important complement to personalised immunotherapy. WGCNA further identified a turquoise module that is closely associated with SIC. This module contains a set of key genes that may synergistically contribute to the ground inflammatory response during sepsis. Immune infiltration analyses showed that increased DVL1 expression levels were closely associated with increased infiltration of pro-inflammatory immune cells (e.g., macrophages and T-cells), suggesting that DVL1 may influence the susceptibility of SIC patients by modulating the behavior of immune cells in the sepsis microenvironment. These results suggest that SIC is, at least in part, an immune-mediated disease and further reveal a central role for 138DVL1 in the regulation of inflammation.

In a broader biological context, this study revealed DVL1 as a key molecular link between sepsis, cancer, and cardiac dysfunction (31, 127).DVL1 is a core regulator of the Wnt signaling pathway and plays important roles in biological processes such as cell proliferation, differentiation, and migration (128, 129). Through bioinformatics analysis and experimental verification, studying the association of signaling pathways and diseases has become an important direction of current medical research, among which the research on the Wnt signaling pathway has yielded fruitful results (130).These studies provide new targets and ideas of disease treatment, further highlighting the significance for SIC therapy in the research focusing on DVL1 and the Wnt signaling pathway.DVL1, as a central mediator of the Wnt signaling pathway, modulates downstream signaling upon Wnt signaling activation by interacting with the frizzled receptor as well as Lrp5/6. It promotes the accumulation of β-cyclins by inhibiting GSK-3β and bringing it into the nucleus, which ultimately affects the expression of downstream genes (131, 132). Aberrant activation of the Wnt signaling pathway may promote pathological remodeling of cardiac tissue associated with SIC, especially in cancer patients in a hyperinflammatory state (133, 134). Because to the central role of Wnt signaling in immune regulation, this mechanism can help to understand the emergence of immunotherapy resistance in certain cancer subtypes.

This study also explored the epigenetic mechanisms of DVL1 transcriptional regulation, combined with ATAC-seq data. The methods of this study borrowed and combineed advanced technology and experimental procedures of several documents (135–137). In this process, big data and bioinformatics technologies play a key role. Their use in biomarker identification is increasingly important to aid in the diagnosis and prognostic assessment of the disease (138). We found that the open chromatin state of the DVL1 promoter and enhancer regions is closely associated with the binding of multiple transcription factors, including key transcriptional regulators such as MYC, NF- κ B and STAT 3. These transcription factors all play important roles in the inflammatory response, tumor progression, and immune regulation. The abnormal activation of MYC may aggravate the inflammatory response in the tumor microenvironment by enhancing DVL1 expression, and the synergistic action of NF- κ B and STAT 3 may further drive the pathological progression of SIC. It has been shown that the open state of chromatin not only determines gene accessibility, but also affects the extent to which tumor cells respond to immunotherapy. In the study of cancer immunotherapy, new mechanisms of immune cells have been explored from the aspects of epigenetic modification, metabolic regulation and intercellular communication to provide a theoretical basis for the optimization of cancer immunotherapy strategies (139). These studies highlight the important role of bioinformatics and transcriptomic approaches in resolving the tumor immune microenvironment and provide new perspectives for the development of future targeted therapeutic protocols (140, 141). This has similarities with our study on the role of DVL1 in SIC and the therapeutic strategy. Therefore, we hypothesized that targeted regulation of DVL1-associated epigenetic regulatory networks may help to optimize therapeutic strategies for SIC and related tumors. The study of biomarkers is a key link in early disease diagnosis and precision treatment (142). Using multi-omics analysis technology and combined with bioinformatics means it can reveal the potential markers of diseases from the molecular level, such as the identification of disease-related MicroRNAs, metabolic fingerprint maps and extracellular vesicle surface proteins from biological samples such as bile and serum, opening up a new way for the early detection of diseases and disease monitoring (143). The open chromatin regions revealed by ATAC-seq data can be used to screen patients who respond to DVL1 targeted therapy to enable precision treatment. Moreover, combined with single-cell sequencing technology, it is expected to further investigate the role of DVL1 in different immune cell subsets and reveal its dynamic changes in the remodeling of the immune microenvironment. These findings provide new perspectives for future research on targeted intervention strategies for DVL1. Despite the importance of this study, some limitations remain. First, we focused on the direct effect of DVL1 on cardiomyocytes, and did not deeply investigate its specific role in VSMCs in SIC (48, 56). VSMCs play a key role in maintaining vascular homeostasis and vascular remodeling processes, and their response during sepsis may influence the pathological progression of SIC (60, 144). Future studies investigating DVL1 function in VSMCs and analyzing its effects on vascular dysfunction should be performed to refine the mechanism of DVL1 action in the pathogenesis of SIC. Second, although this study explored the pharmacological intervention strategies for DVL1, its synergy with non-pharmacological interventions (e.g., exercise) has not been fully evaluated (145, 146). Exercise has been shown to improve the course of SIC by modulating the Wnt signaling pathway and reducing oxidative stress and inflammation.

In the future, further research can be made to investigate whether exercise improves SIC by affecting the DVL1 signaling pathway and evaluate the effect of combined exercise and drug intervention to optimize the comprehensive treatment regimen of SIC. Moreover, this study has some limitations in terms of sample size and dataset. The sample size may not be sufficiently representative of all potential patient population characteristics.The data in the database used may also have geographical, ethnic and other bias, thus affecting the generalizability of the study results. Future studies should also cover a wider range of cell types and explore in-depth the function of DVL1 in different microenvironments (e.g., tumor microenvironment and immune system).At the same time, the development of “off-the-shelf” gene therapy nanoparticles based on existing drugs or the use of CRISPR technology also provides new possibilities for clinical applications in medicine (147, 148). Furthermore, through in-depth analysis of patient engagement and social support systems, the investigators revealed the important impact of these factors on disease management and patient mental health (149, 150). Future studies on SIC can consider including these psychosocial factors in the research category, comprehensively evaluate their interaction with biological factors, develop more comprehensive and effective treatment and management programs, and promote the overall recovery of patients.DVL1 expression is upregulated in a variety of cancers (such as colorectal and gastric cancers) and is closely associated with the abnormal activation of the Wnt signaling pathway. Understanding these interactions could provide new insights into how DVL1 mediates immune responses in the SIC. Finally, the main conclusions of this study are based on *in vitro* experiments and bioinformatics analysis, and lack support from *in vivo* experimental and clinical data. In the future, mouse SIC models should be constructed to verify the efficacy of DVL1 inhibitors (such as Digoxin) in SIC treatment and evaluate the correlation of DVL1 expression level and the prognosis of SIC patients combined with clinical data. The further development of animal experiments and clinical research will provide stronger evidence for the wide application of DVL1-targeted drugs in SIC treatment. Although this study focuses on the interaction of post-tumor inflammation with the dysfunction of VSMC and the mechanism of DVL1 in sepsis-induced cardiomyopathy (SIC), the field of medical research is broad and interconnected (43, 151). The coding research of the biological meta-universe explores the progress and challenges in the neural field from the macro level to the micro level, combined with the simulation of the nervous system information transmission, and lays the foundation for the future development of human-computer interaction and neuroregulation technology (152). In terms of microbial research, the study of the gut microbiome has always been a hot topic (153, 154). Using metagenomic sequencing technology combined with bioinformatics data analysis to deeply explore the interactions between microorganisms in the gut,is important for understanding the relationship between human health and disease. Microorganisms are also emerging in drug delivery. bacteria-based drug delivery systems have opened up new ways for the treatment of non-neoplastic diseases, showing unique therapeutic advantages (155). In this study, although the microbiome-related content was not directly involved, the microbiome combined with bioinformatics analysis can also open up new ways for the diagnosis and treatment of diseases (156, 157). In the future, in the study of SIC and related diseases, we may learn from the ideas and methods of microbial research, use bioinformatics to analyze the pathogen genome and host immune response data, deeply explore the relationship between microorganisms and diseases in SIC patients, and provide multi-dimensional support for the development of more effective treatment strategies (158, 159). This also suggests that in future studies, we should focus on the integration of research results in different fields and explore the pathogenesis and treatment strategies of SIC from a broader perspective (8, 121). In conclusion, this study reveals the critical role of DVL1 in the pathogenesis of SIC and provides strong evidence as a novel therapeutic target, providing important clues for precision medicine of SIC. Moreover, the synergistic effects of pharmacological and non-pharmacological interventions still deserve intensive investigation in the hope of providing more effective personalized treatment options for patients with SIC (160).In this process, big data and bioinformatics technologies play a pivotal role, particularly in biomarker identification, which is increasingly important for aiding the diagnosis and prognostic assessment of diseases (138). In the future, combining big data analysis, bioinformatics means, and multi-level experimental validation, it is expected to further elucidate the regulatory network of DVL1 and optimize its targeted intervention strategies (161, 162). The findings of this study not only deepen the understanding of the molecular mechanisms of DVL1, but also establish the theoretical basis for future personalized treatment strategies for patients with SIC (163, 164). Research of biomarkers is crucial for the early diagnosis and precise treatment of diseases (142, 165). In this study, multi-omics analysis techniques and bioinformatics means were used to study the DVL1 gene as a potential biomarker (125, 166). In the future, further research is expected to identify potential markers such as MicroRNAs, metabolic fingerprints, and extracellular vesicle surface proteins from more biological samples so as to provide more ways for early disease detection and disease monitoring and promote the development of personalized treatment for SIC and related diseases (143). With the continuous advancement of research, DVL1 will become a new breakthrough in the treatment of SIC and even cancer-related cardiovascular diseases, contributing to the development of new treatment methods (167–169).

## 5 Conclusion

This study highlights DVL1 as a key gene in SIC and its association with poor outcomes in cancer, particularly in the context of immunotherapy resistance. DVL1’s upregulation is linked to increased inflammation and unfavorable prognosis, suggesting its role in the complex landscape of intratumor heterogeneity. Molecular docking identified Digoxin as a promising candidate for targeting DVL1, with the potential to reduce oxidative stress and modulate immune responses in SIC. WGCNA further confirmed the central role of DVL1 in the gene network driving disease progression. These findings underscore the potential of targeting DVL1 to improve therapeutic outcomes in SIC and cancer, particularly when integrated with pharmacotherapy and exercise regimens. By addressing the challenges posed by intratumor heterogeneity, this study offers new insights into the molecular mechanisms underlying SIC and its overlap with cancer progression. Further clinical research is needed to validate the therapeutic potential of targeting DVL1, aiming to enhance immunotherapy effectiveness and provide more personalized treatment strategies for patients facing both SIC and cancer.

## Data Availability

The original contributions presented in the study are included in the article/**Supplementary Material**. Further inquiries can be directed to the corresponding authors.

## References

[1] Lin Y-M Lee M-C Toh HS Chang W-T Chen S-Y Kuo F-H. Association of sepsis-induced cardiomyopathy and mortality: a systematic review and meta-analysis. Ann Intensive Care. (2022) 12:112. doi: 10.1186/s13613-022-01089-3 36513882 PMC9748009

[2] Hanumanthu BKJ Nair AS Katamreddy A Gilbert JS You JY Offor OL. Sepsis-induced cardiomyopathy is associated with higher mortality rates in patients with sepsis. Acute Crit Care. (2021) 36:215–22. doi: 10.4266/acc.2021.00234 PMC8435447 34311515

[3] Wang Y Zhou J Wu K. High 28-day mortality in critically ill patients with sepsis and concomitant active cancer. J Int Med Res. (2018) 46:5030–9. doi: 10.1177/0300060518789040 PMC6300972 30088429

[4] Nazer L Lopez-Olivo MA Cuenca JA Awad W Brown AR Abusara A. All-cause mortality in cancer patients treated for sepsis in intensive care units: a systematic review and meta-analysis. Support Care Cancer. (2022) 30:10099–109. doi: 10.1007/s00520-022-07392-w PMC9549043 36214879

[5] Kanterman J Sade-Feldman M Baniyash M. New insights into chronic inflammation-induced immunosuppression. Semin Cancer Biol. (2012) 22:307–18. doi: 10.1016/j.semcancer.2012.02.008 22387003

[6] Shalapour S Karin M. Immunity, inflammation, and cancer: an eternal fight between good and evil. J Clin Invest. (2015) 125:3347–55. doi: 10.1172/JCI80007 PMC4588298 26325032

[7] Mirouse A Vigneron C Llitjos J-F Chiche J-D Mira J-P Mokart D. Sepsis and cancer: an interplay of friends and foes. Am J Respir Crit Care Med. (2020) 202:1625–35. doi: 10.1164/rccm.202004-1116TR 32813980

[8] Williams B Zou L Pittet J-F Chao W. Sepsis-induced coagulopathy: A comprehensive narrative review of pathophysiology, clinical presentation, diagnosis, and management strategies. Anesth Analg. (2024) 23. doi: 10.1213/ANE.0000000000006888 PMC10916756 38324297

[9] Palaskas NL Ali H-J Koutroumpakis E Ganatra S Deswal A. Cardiovascular toxicity of immune therapies for cancer. BMJ. (2024) 385:e075859. doi: 10.1136/bmj-2023-075859 38749554

[10] Totzeck M Schuler M Stuschke M Heusch G Rassaf T. Cardio-oncology - strategies for management of cancer-therapy related cardiovascular disease. Int J Cardiol. (2019) 280:163–75. doi: 10.1016/j.ijcard.2019.01.038 30661849

[11] Heller G Fuereder T Grandits AM Wieser R. New perspectives on biology, disease progression, and therapy response of head and neck cancer gained from single cell RNA sequencing and spatial transcriptomics. Oncol Res. (2024) 32:1–17. doi: 10.32604/or.2023.044774 PMC10767240 38188682

[12] Guo J Tong C Shi J Li X Chen X. A prognosis model for predicting immunotherapy response of esophageal cancer based on oxidative stress-related signatures. Oncol Res. (2024) 32:199–212. doi: 10.32604/or.2023.030969 PMC10774069 38196829

[13] Xu J Wang F Li Y Li P Zhang Y Xu G. Estrogen inhibits TGF−β1−stimulated cardiac fibroblast differentiation and collagen synthesis by promoting Cdc42. Mol Med Rep. (2024) 30:123. doi: 10.3892/mmr.2024.13246 38785153 PMC11130745

[14] Gao D Ge G. Exploring the underlying biology of cancer and potential therapeutic strategies: a special issue focused on mechanism-based studies. Acta Biochim Biophys Sin. (2023) 55:891–2. doi: 10.3724/abbs.2023114 PMC10326411 37337636

[15] Hu D Sheeja Prabhakaran H Zhang Y-Y Luo G He W Liou Y-C. Mitochondrial dysfunction in sepsis: mechanisms and therapeutic perspectives. Crit Care. (2024) 28:292. doi: 10.1186/s13054-024-05069-w 39227925 PMC11373266

[16] Galley HF. Oxidative stress and mitochondrial dysfunction in sepsis. Br J Anaesth. (2011) 107:57–64. doi: 10.1093/bja/aer093 21596843

[17] Mourad M Chow-Chine L Faucher M Sannini A Brun JP De Guibert JM. Early diastolic dysfunction is associated with intensive care unit mortality in cancer patients presenting with septic shock. Br J Anaesth. (2014) 112:102–9. doi: 10.1093/bja/aet296 24046293

[18] Tirichen H Yaigoub H Xu W Wu C Li R Li Y. Mitochondrial reactive oxygen species and their contribution in chronic kidney disease progression through oxidative stress. Front Physiol. (2021) 12:627837. doi: 10.3389/fphys.2021.627837 33967820 PMC8103168

[19] Sanderson TH Reynolds CA Kumar R Przyklenk K Hüttemann M. Molecular mechanisms of ischemia–reperfusion injury in brain: pivotal role of the mitochondrial membrane potential in reactive oxygen species generation. Mol Neurobiol. (2013) 47:9–23. doi: 10.1007/s12035-012-8344-z 23011809 PMC3725766

[20] Arba F Ferretti S Leigh R Fara A Warach SJ Luby M. Cerebral small vessel disease and infarct growth in acute ischemic stroke treated with intravenous thrombolysis. Transl Stroke Res. (2024) 24. doi: 10.1007/s12975-024-01277-2 38963535

[21] Zhang W Liu Y Zhou J Qiu T Xie H Pu Z. Chicoric acid advanced PAQR3 ubiquitination to ameliorate ferroptosis in diabetes nephropathy through the relieving of the interaction between PAQR3 and P110α pathway. Clin Exp Hypertens. (2024) 46:2326021. doi: 10.1080/10641963.2024.2326021 38525833

[22] Xu W Gao X Luo H Chen Y. FGF21 attenuates salt-sensitive hypertension via regulating HNF4α/ACE2 axis in the hypothalamic paraventricular nucleus of mice. Clin Exp Hypertens. (2024) 46:2361671. doi: 10.1080/10641963.2024.2361671 38841901

[23] Chen D Zhang X Li Z Zhu B. Metabolic regulatory crosstalk between tumor microenvironment and tumor-associated macrophages. Theranostics. (2021) 11:1016–30. doi: 10.7150/thno.51777 PMC7738889 33391518

[24] Wang N Liang H Zen K. Molecular mechanisms that influence the macrophage M1â€”M2 polarization balance. Front Immunol. (2014) 5:614. doi: 10.3389/fimmu.2014.00614 25506346 PMC4246889

[25] Thakur A Banerjee R Thakur S Kumar G Thakur SS. Role of macrophage polarization in cancer progression and their association with COVID-19 severity. Cancer Insight. (2023) 24. doi: 10.58567/ci02010005

[26] Wang Y Liu W Zhang J Geng P Jin X. The role of crosstalk between cerebral immune cells and peripheral immune cells in the damage and protection of blood–brain barrier after intracerebral hemorrhage. Brain Hemorrhages. (2024) 5:117–30. doi: 10.1016/j.hest.2024.02.002

[27] Peters Van Ton AM Kox M Abdo WF Pickkers P. Precision immunotherapy for sepsis. Front Immunol. (2018) 9:1926. doi: 10.3389/fimmu.2018.01926 30233566 PMC6133985

[28] Patil NK Bohannon JK Sherwood ER. Immunotherapy: A promising approach to reverse sepsis-induced immunosuppression. Pharmacol Res. (2016) 111:688–702. doi: 10.1016/j.phrs.2016.07.019 27468649 PMC5026606

[29] Rudiger A Singer M. Mechanisms of sepsis-induced cardiac dysfunction. Crit Care Med. (2007) 35:1599–608. doi: 10.1097/01.CCM.0000266683.64081.02 17452940

[30] Potz B A Sellke FW Abid MR. Endothelial ROS and impaired myocardial oxygen consumption in sepsis-induced cardiac dysfunction. J Intensive Crit Care. (2016) 02:24. doi: 10.21767/2471-8505.100020 PMC4847432 27135058

[31] Huang H Wang Q Ma L Wu Y. ITGAM: A pivotal regulator in macrophage dynamics and cardiac function during sepsis-induced cardiomyopathy. Cureus. (2024) 24. doi: 10.7759/cureus.59342 PMC11070384 38711712

[32] Del Bufalo D Bagnato A Fusco A Milella M. Lost in translation: bridging the gap between cancer research and effective therapies. Cell Death Differ. (2011) 18:1082–4. doi: 10.1038/cdd.2010.186 PMC3131946 21252913

[33] Stanczak MA Läubli H. Siglec receptors as new immune checkpoints in cancer. Mol Aspects Med. (2023) 90:101112. doi: 10.1016/j.mam.2022.101112 35948467

[34] Clària J Arroyo V Moreau R. The acute-on-chronic liver failure syndrome, or when the innate immune system goes astray. J Immunol. (2016) 197:3755–61. doi: 10.4049/jimmunol.1600818 27815438

[35] Alonso De Vega JM Díaz J Serrano E Carbonell LF. Oxidative stress in critically ill patients with systemic inflammatory response syndrome. Crit Care Med. (2002) 30:1782–6. doi: 10.1097/00003246-200208000-00018 12163793

[36] Zanotti S Kumar A Kumar A. Cytokine modulation in sepsis and septic shock. Expert Opin Investig Drugs. (2002) 11:1061–75. doi: 10.1517/13543784.11.8.1061 12150702

[37] Houschyar KS Pyles MN Rein S Nietzschmann I Duscher D Maan ZN. Continuous hemoadsorption with a cytokine adsorber during sepsis – a review of the literature. Int J Artif Organs. (2017) 40:205–11. doi: 10.5301/ijao.5000591 28525674

[38] Mann DL. Stress-activated cytokines and the heart: from adaptation to maladaptation. Annu Rev Physiol. (2003) 65:81–101. doi: 10.1146/annurev.physiol.65.092101.142249 12500970

[39] Mehra VC Ramgolam VS Bender JR. Cytokines and cardiovascular disease. J Leukoc Biol. (2005) 78:805–18. doi: 10.1189/jlb.0405182 16006537

[40] Williams JC Ford ML Coopersmith CM. Cancer and sepsis. Clin Sci. (2023) 137:881–93. doi: 10.1042/CS20220713 PMC10635282 37314016

[41] Drosatos K Lymperopoulos A Kennel PJ Pollak N Schulze PC Goldberg IJ. Pathophysiology of sepsis-related cardiac dysfunction: driven by inflammation, energy mismanagement, or both? Curr Heart Fail Rep. (2015) 12:130–40. doi: 10.1007/s11897-014-0247-z PMC4474734 25475180

[42] D’Oria R Schipani R Leonardini A Natalicchio A Perrini S Cignarelli A. The role of oxidative stress in cardiac disease: from physiological response to injury factor. Oxid Med Cell Longev. (2020) 2020:1–29. doi: 10.1155/2020/5732956 PMC7244977 32509147

[43] Liu Y-C Yu M-M Shou S-T Chai Y-F. Sepsis-induced cardiomyopathy: mechanisms and treatments. Front Immunol. (2017) 8:1021. doi: 10.3389/fimmu.2017.01021 28970829 PMC5609588

[44] Yao J-Y Yang Y-L Chen W-J Fan H-Y. Exploring the therapeutic potential of Qi Teng Mai Ning recipe in ischemic stroke and vascular cognitive impairment. Tradit Med Res. (2024) 9:57. doi: 10.53388/TMR20240214001

[45] Pourbagheri-Sigaroodi A Fallah F Momeny M Rezaei N Bashash D. Unveiling the predictive power of bacterial response-related genes signature in hepatocellular carcinoma: with bioinformatics analyses and experimental approaches. BIOCELL. (2024) 48:1781–804. doi: 10.32604/biocell.2024.055848

[46] Lv X Mao Z Sun X Liu B. Intratumoral heterogeneity in lung cancer. Cancers. (2023) 15:2709. doi: 10.3390/cancers15102709 37345046 PMC10216154

[47] Janiszewska M. The microcosmos of intratumor heterogeneity: the space-time of cancer evolution. Oncogene. (2020) 39:2031–9. doi: 10.1038/s41388-019-1127-5 PMC7374939 31784650

[48] Zaky A Deem S Bendjelid K Treggiari MM. Characterization of cardiac dysfunction in sepsis: an ongoing challenge. Shock. (2014) 41:12–24. doi: 10.1097/SHK.0000000000000065 24351526

[49] Comen EA Bowman RL Kleppe M. Underlying causes and therapeutic targeting of the inflammatory tumor microenvironment. Front Cell Dev Biol. (2018) 6:56. doi: 10.3389/fcell.2018.00056 29946544 PMC6005853

[50] McAllister SS Weinberg RA. The tumour-induced systemic environment as a critical regulator of cancer progression and metastasis. Nat Cell Biol. (2014) 16:717–27. doi: 10.1038/ncb3015 PMC6220424 25082194

[51] Nieminen MS Dickstein K Fonseca C Serrano JM Parissis J Fedele F. The patient perspective: Quality of life in advanced heart failure with frequent hospitalisations. Int J Cardiol. (2015) 191:256–64. doi: 10.1016/j.ijcard.2015.04.235 25981363

[52] Sila CA. Cognitive impairment in chronic heart failure. Cleve Clin J Med. (2007) 74:S132–2. doi: 10.3949/ccjm.74.Suppl_1.S132 17455562

[53] Omerovic E. Did Jesus die of a ‘broken heart’? Eur J Heart Fail. (2009) 11:729–31. doi: 10.1093/eurjhf/hfp095 19633099

[54] Oyinbo C. Secondary injury mechanisms in traumatic spinal cord injury: a nugget of this multiply cascade. Acta Neurobiol Exp (Warsz). (2011) 71:281–99. doi: 10.55782/ane-2011-1848 21731081

[55] Song J Ren K Zhang D Lv X Sun L Deng Y. A novel signature combing cuproptosis- and ferroptosis-related genes in sepsis-induced cardiomyopathy. Front Genet. (2023) 14:1170737. doi: 10.3389/fgene.2023.1170737 37035738 PMC10076593

[56] Sorokin V Vickneson K Kofidis T Woo CC Lin XY Foo R. Role of vascular smooth muscle cell plasticity and interactions in vessel wall inflammation. Front Immunol. (2020) 11:599415. doi: 10.3389/fimmu.2020.599415 33324416 PMC7726011

[57] Orr AW Hastings NE Blackman BR Wamhoff BR. Complex regulation and function of the inflammatory smooth muscle cell phenotype in atherosclerosis. J Vasc Res. (2010) 47:168–80. doi: 10.1159/000250095 PMC2842170 19851078

[58] Sergi C Shen F Lim DW Liu W Zhang M Chiu B. Cardiovascular dysfunction in sepsis at the dawn of emerging mediators. BioMed Pharmacother. (2017) 95:153–60. doi: 10.1016/j.biopha.2017.08.066 28841455

[59] Zanotti-Cavazzoni SL Hollenberg SM. Cardiac dysfunction in severe sepsis and septic shock. Curr Opin Crit Care. (2009) 15:392–7. doi: 10.1097/MCC.0b013e3283307a4e 19633546

[60] Strela FB Brun BF Berger RCM Melo S De Oliveira EM Barauna VG. Lipopolysaccharide exposure modulates the contractile and migratory phenotypes of vascular smooth muscle cells. Life Sci. (2020) 241:117098. doi: 10.1016/j.lfs.2019.117098 31794773

[61] Tang H-Y Chen A-Q Zhang H Gao X-F Kong X-Q Zhang J-J. Vascular smooth muscle cells phenotypic switching in cardiovascular diseases. Cells. (2022) 11:4060. doi: 10.3390/cells11244060 36552822 PMC9777337

[62] Ashraf JV Al Haj Zen A. Role of vascular smooth muscle cell phenotype switching in arteriogenesis. Int J Mol Sci. (2021) 22:10585. doi: 10.3390/ijms221910585 34638923 PMC8508942

[63] Nie Q Zhang J He B Wang F Sun M Wang C. A novel mechanism of protection against isoproterenol-induced cardiac inflammation via regulation of the SIRT1/NRF2 signaling pathway with a natural SIRT1 agonist. Eur J Pharmacol. (2020) 886:173398. doi: 10.1016/j.ejphar.2020.173398 32763301

[64] Wang R Peng X Yuan Y Shi B Liu Y Ni H. Dynamic immune recovery process after liver transplantation revealed by single-cell multi-omics analysis. Innovation. (2024) 5:100599. doi: 10.1016/j.xinn.2024.100599 38510071 PMC10952083

[65] Zhang J He J Chen W Chen G Wang L Liu Y. Single-cell RNA-binding protein pattern-mediated molecular subtypes depict the hallmarks of the tumor microenvironment in bladder urothelial carcinoma. Oncologie. (2024) 26:657–69. doi: 10.1515/oncologie-2024-0071

[66] Raines EW Ferri N. Thematic Review Series: The Immune System and Atherogenesis. Cytokines affecting endothelial and smooth muscle cells in vascular disease. J Lipid Res. (2005) 46:1081–92. doi: 10.1194/jlr.R500004-JLR200 15834121

[67] Feng D Li D Wang J Wu R Zhang C. Senescence-associated lncRNAs indicate distinct molecular subtypes associated with prognosis and androgen response in patients with prostate cancer. Acta Mater Med. (2023) 2:24. doi: 10.15212/AMM-2023-0025

[68] Zhang M Otsuki K Li W. Molecular networking as a natural products discovery strategy. Acta Mater Med. (2023) 2:24. doi: 10.15212/AMM-2023-0007

[69] Vanhoutte D Schellings MWM Götte M Swinnen M Herias V Wild MK. Increased expression of syndecan-1 protects against cardiac dilatation and dysfunction after myocardial infarction. Circulation. (2007) 115:475–82. doi: 10.1161/CIRCULATIONAHA.106.644609 17242279

[70] Shimada BK Boyman L Huang W Zhu J Yang Y Chen F. Pyruvate-driven oxidative phosphorylation is downregulated in sepsis-induced cardiomyopathy: A study of mitochondrial proteome. Shock. (2022) 57:553–64. doi: 10.1097/SHK.0000000000001858 PMC8904652 34506367

[71] Verma SK Garikipati VNS Kishore R. Mitochondrial dysfunction and its impact on diabetic heart. Biochim Biophys Acta BBA - Mol Basis Dis. (2017) 1863:1098–105. doi: 10.1016/j.bbadis.2016.08.021 PMC5332436 27593695

[72] Lin J Kirshenbaum LA. Wnt-1 dishevelled signaling functionally links calcium-calmodulin–dependent protein kinase II and cardiac dysfunction. Hypertension. (2015) 65:287–8. doi: 10.1161/HYPERTENSIONAHA.114.04616 25489063

[73] Chen Y-R Zweier JL. Cardiac mitochondria and reactive oxygen species generation. Circ Res. (2014) 114:524–37. doi: 10.1161/CIRCRESAHA.114.300559 PMC4118662 24481843

[74] Gao D Yang J Wu Y Wang Q Wang Q Lai EY. Targeting dynamin 2 as a novel pathway to inhibit cardiomyocyte apoptosis following oxidative stress. Cell Physiol Biochem. (2016) 39:2121–34. doi: 10.1159/000447908 27802433

[75] Costa R Peruzzo R Bachmann M Montà GD Vicario M Santinon G. Impaired mitochondrial ATP production downregulates wnt signaling via ER stress induction. Cell Rep. (2019) 28:1949–1960.e6. doi: 10.1016/j.celrep.2019.07.050 31433973

[76] Arrázola MS Silva-Alvarez C Inestrosa NC. How the Wnt signaling pathway protects from neurodegeneration: the mitochondrial scenario. Front Cell Neurosci. (2015) 9:166. doi: 10.3389/fncel.2015.00166 25999816 PMC4419851

[77] Kuroshima T Kawaguchi S Okada M. Current perspectives of mitochondria in sepsis-induced cardiomyopathy. Int J Mol Sci. (2024) 25:4710. doi: 10.3390/ijms25094710 38731929 PMC11083471

[78] Haybar H Shahrouzian M Gatavizadeh Z Saki N Maniati M Deris Zayeri Z. Cyclin D1: A golden gene in cancer, cardiotoxicity, and cardioprotection. Jundishapur J Chronic Dis Care. (2021) 10. doi: 10.5812/jjcdc.112413

[79] Ramel D Gayral S Sarthou M-K Augé N Nègre-Salvayre A Laffargue M. Immune and smooth muscle cells interactions in atherosclerosis: how to target a breaking bad dialogue? Front Pharmacol. (2019) 10:1276. doi: 10.3389/fphar.2019.01276 31824304 PMC6882774

[80] Sabe VT Ntombela T Jhamba LA Maguire GEM Govender T Naicker T. Current trends in computer aided drug design and a highlight of drugs discovered via computational techniques: A review. Eur J Med Chem. (2021) 224:113705. doi: 10.1016/j.ejmech.2021.113705 34303871

[81] Wu Z Chen S Wang Y Li F Xu H Li M. Current perspectives and trend of computer-aided drug design: a review and bibliometric analysis. Int J Surg. (2024) 24. doi: 10.1097/JS9.0000000000001289 PMC11175770 38502850

[82] Huang W-B Qin S-Y Zou J-B Li X Kang W-L Yuan P-W. Efficacy of Juanbi capsule on ameliorating knee osteoarthritis: a network pharmacology and experimental verification-based study. Tradit Med Res. (2024) 9:33. doi: 10.53388/TMR20230829002

[83] Zhang J Zhao F Ma B Shen X Geng Y. Fluoxetine inhibited RANKL-induced osteoclastic differentiation *in vitro* . Open Med. (2024) 19:20241094. doi: 10.1515/med-2024-1094 PMC11662947 39711842

[84] Lou C Pang C Jing C Wang S He X Liu X. Dynamic balance measurement and quantitative assessment using wearable plantar-pressure insoles in a pose-sensed virtual environment. Sensors. (2018) 18:4193. doi: 10.3390/s18124193 30513590 PMC6308589

[85] Hussen BM Taheri M RKh Y Gh A Sr A Rk K. Revolutionizing medicine: recent developments and future prospects in stem-cell therapy. Int J Surg. (2024) 110:8002–24. doi: 10.1097/JS9.0000000000002109 PMC11634165 39497543

[86] Zhou W Wu J Zhang J Liu X Guo S Jia S. Integrated bioinformatics analysis to decipher molecular mechanism of compound Kushen injection for esophageal cancer by combining WGCNA with network pharmacology. Sci Rep. (2020) 10:12745. doi: 10.1038/s41598-020-69708-2 32728182 PMC7391752

[87] Chu C Sun W Chen S Jia Y Ni Y Wang S. Squid-inspired anti-salt skin-like elastomers with superhigh damage resistance for aquatic soft robots. Adv Mater. (2024) 36:2406480. doi: 10.1002/adma.202406480 39267419

[88] Ni Y Li B Chu C Wang S Jia Y Cao S. One-step fabrication of ultrathin porous Janus membrane within seconds for waterproof and breathable electronic skin. Sci Bull. (2024) S2095927324009502. doi: 10.1016/j.scib.2024.12.040 39837718

[89] Liu D Wang T Wang Q Dong P Liu X Li Q. Identification of key genes in sepsis-induced cardiomyopathy based on integrated bioinformatical analysis and experiments *in vitro* and *in vivo* . PeerJ. (2023) 11:e16222. doi: 10.7717/peerj.16222 38025678 PMC10668858

[90] Li J Zhou L Li Z Yang S Tang L Gong H. Identification of crucial genes and infiltrating immune cells underlying sepsis-induced cardiomyopathy via weighted gene co-expression network analysis. Front Genet. (2021) 12:812509. doi: 10.3389/fgene.2021.812509 35003233 PMC8740124

[91] Chen Y Chen X Luo Z Kang X Ge Y Wan R. Exercise-induced reduction of IGF1R sumoylation attenuates neuroinflammation in APP/PS1 transgenic mice. J Adv Res. (2024) S2090123224001279. doi: 10.1016/j.jare.2024.03.025 PMC11954827 38565402

[92] Chen Y Huang L Luo Z Han D Luo W Wan R. Pantothenate-encapsulated liposomes combined with exercise for effective inhibition of CRM1-mediated PKM2 translocation in Alzheimer’s therapy. J Controlled Release. (2024) 373:336–57. doi: 10.1016/j.jconrel.2024.07.010 38996921

[93] Chen Y Luo Z Sun Y Li F Han Z Qi B. Exercise improves choroid plexus epithelial cells metabolism to prevent glial cell-associated neurodegeneration. Front Pharmacol. (2022) 13:1010785. doi: 10.3389/fphar.2022.1010785 36188600 PMC9523215

[94] Wu Y Li Y Wu T Yang H. The dual roles of S-nitrosylation of proteins in cancer: molecular mechanisms and recent advancements. Cancer Insight. (2024) 3:37–48. doi: 10.58567/ci03020005

[95] Yuan L Liu Y Sun Y Ren L Gu X Chen L. Puerarin attenuates remifentanil−induced postoperative hyperalgesia via targeting PAX6 to regulate the transcription of TRPV1. Mol Med Rep. (2024) 29:81. doi: 10.3892/mmr.2024.13204 38516772 PMC10975072

[96] Yauch RL Settleman J. Recent advances in pathway-targeted cancer drug therapies emerging from cancer genome analysis. Curr Opin Genet Dev. (2012) 22:45–9. doi: 10.1016/j.gde.2012.01.003 22321987

[97] Nicolaides NC Sass PM Grasso L. Advances in targeted therapeutic agents. Expert Opin Drug Discovery. (2010) 5:1123–40. doi: 10.1517/17460441.2010.521496 22827748

[98] Chistiakov DA Orekhov AN Bobryshev YV. Vascular smooth muscle cell in atherosclerosis. Acta Physiol. (2015) 214:33–50. doi: 10.1111/apha.12466 25677529

[99] Clarke MCH Figg NL Bennett MR. Vascular smooth muscle cell apoptosis induces interleukin-1–directed inflammation: effects of hyperlipidemia-mediated inhibition of phagocytosis. Circ Res. (2010) 106:363–72. doi: 10.1161/CIRCRESAHA.109.208389 19926874

[100] Marques L Costa B Pereira M Silva A Santos J Saldanha L. Advancing precision medicine: A review of innovative in silico approaches for drug development, clinical pharmacology and personalized healthcare. Pharmaceutics. (2024) 16:332. doi: 10.3390/pharmaceutics16030332 38543226 PMC10975777

[101] Maggi E Patterson NE Montagna C. Technological advances in precision medicine and drug development. Expert Rev Precis Med Drug Dev. (2016) 1:331–43. doi: 10.1080/23808993.2016.1176527 PMC5017245 27622214

[102] Chen M Kong C Zheng Z Li Y. Identification of biomarkers associated with septic cardiomyopathy based on bioinformatics analyses. J Comput Biol. (2020) 27:69–80. doi: 10.1089/cmb.2019.0181 31424269

[103] Long Q Li G Dong Q Wang M Li J Wang L. Exploration of the shared gene signatures between myocardium and blood in sepsis: evidence from bioinformatics analysis. BioMed Res Int. (2022) 2022:1–16. doi: 10.1155/2022/3690893 PMC9375705 35971449

[104] Huang D Zheng S Liu Z Zhu K Zhi H Ma G. Machine learning revealed ferroptosis features and a novel ferroptosis-based classification for diagnosis in acute myocardial infarction. Front Genet. (2022) 13:813438. doi: 10.3389/fgene.2022.813438 35145551 PMC8821875

[105] Cirulis MM Beesley SJ Wilson EL Stubben C Olsen TD Hirshberg EL. The peripheral blood transcriptome in septic cardiomyopathy: an observational, pilot study. Intensive Care Med Exp. (2019) 7:57. doi: 10.1186/s40635-019-0271-0 31650252 PMC6813402

[106] Wu P Nielsen TE Clausen MH. Small-molecule kinase inhibitors: an analysis of FDA-approved drugs. Drug Discovery Today. (2016) 21:5–10. doi: 10.1016/j.drudis.2015.07.008 26210956

[107] Lin H-H Zhang L-L Yan R Lu J-J Hu Y. Network analysis of drug–target interactions: A study on FDA-approved new molecular entities between 2000 to 2015. Sci Rep. (2017) 7:12230. doi: 10.1038/s41598-017-12061-8 28947756 PMC5612934

[108] Wishart DS Feunang YD Guo AC Lo EJ Marcu A Grant JR. DrugBank 5.0: a major update to the DrugBank database for 2018. Nucleic Acids Res. (2018) 46:D1074–82. doi: 10.1093/nar/gkx1037 PMC5753335 29126136

[109] Vilar S Harpaz R Uriarte E Santana L Rabadan R Friedman C. Drug—drug interaction through molecular structure similarity analysis. J Am Med Inform Assoc. (2012) 19:1066–74. doi: 10.1136/amiajnl-2012-000935 PMC3534468 22647690

[110] Khatami MH Bromberek M Saika-Voivod I Booth V. Molecular dynamics simulations of histidine-containing cod antimicrobial peptide paralogs in self-assembled bilayers. Biochim Biophys Acta BBA - Biomembr. (2014) 1838:2778–87. doi: 10.1016/j.bbamem.2014.07.013 25058381

[111] Vo ATN Murphy MA Stone TW Phan PK Baskes MI Prabhu RK. Molecular dynamics simulations of phospholipid bilayer mechanoporation under different strain states—a comparison between GROMACS and LAMMPS. Model Simul Mater Sci Eng. (2021) 29:055015. doi: 10.1088/1361-651X/abfeaf

[112] Alves KMA Cardoso FJB Honorio KM De Molfetta FA. Design of inhibitors for glyceraldehyde-3-phosphate dehydrogenase (GAPDH) enzyme of leishmania mexicana. Med Chem. (2020) 16:784–95. doi: 10.2174/1573406415666190712111139 31309897

[113] Guterres H Im W. Improving protein-ligand docking results with high-throughput molecular dynamics simulations. J Chem Inf Model. (2020) 60:2189–98. doi: 10.1021/acs.jcim.0c00057 PMC7534544 32227880

[114] Zhao W. Cyclin D1 in various cancers: Mechanisms and clinical implications. Theor Nat Sci. (2024) 29:87–91. doi: 10.54254/2753-8818/29/20240752

[115] He R Chen Y Qian C Hu Y Huang X Tao R. Dishevelled segment polarity protein 2 promotes gastric cancer progression through Wnt/β-catenin pathway. Tissue Cell. (2023) 82:102119. doi: 10.1016/j.tice.2023.102119 37257286

[116] Yang X Qi Q Pan Y Zhou Q Wu Y Zhuang J. Single-cell analysis reveals characterization of infiltrating T cells in moderately differentiated colorectal cancer. Front Immunol. (2021) 11:620196. doi: 10.3389/fimmu.2020.620196 33584715 PMC7873865

[117] Sun D Wang J Han Y Dong X Ge J Zheng R. TISCH: a comprehensive web resource enabling interactive single-cell transcriptome visualization of tumor microenvironment. Nucleic Acids Res. (2021) 49:D1420–30. doi: 10.1093/nar/gkaa1020 PMC7778907 33179754

[118] Wang Y Ma Q Zhang S Liu H Zhao B Du B. Digoxin enhances the anticancer effect on non-small cell lung cancer while reducing the cardiotoxicity of adriamycin. Front Pharmacol. (2020) 11:186. doi: 10.3389/fphar.2020.00186 32180730 PMC7059749

[119] Zha W Wang G Xu W Liu X Wang Y Zha BS. Inhibition of P-glycoprotein by HIV protease inhibitors increases intracellular accumulation of berberine in murine and human macrophages. PloS One. (2013) 8:e54349. doi: 10.1371/journal.pone.0054349 23372711 PMC3553168

[120] Hardin J Kloke J. Statistical analyses. Curr Protoc Essent Lab Tech. (2017) 14:24. doi: 10.1002/cpet.10

[121] Han Z Quan Z Zeng S Wen L Wang H. Utilizing omics technologies in the investigation of sepsis-induced cardiomyopathy. IJC Heart Vasc. (2024) 54:101477. doi: 10.1016/j.ijcha.2024.101477 PMC11334652 39171080

[122] Chen C Wang Z Ding Y Qin Y. Tumor microenvironment-mediated immune evasion in hepatocellular carcinoma. Front Immunol. (2023) 14:1133308. doi: 10.3389/fimmu.2023.1133308 36845131 PMC9950271

[123] De Visser KE Joyce JA. The evolving tumor microenvironment: From cancer initiation to metastatic outgrowth. Cancer Cell. (2023) 41:374–403. doi: 10.1016/j.ccell.2023.02.016 36917948

[124] Wiejak J Murphy FA Maffia P Yarwood SJ. Vascular smooth muscle cells enhance immune/vascular interplay in a 3-cell model of vascular inflammation. Sci Rep. (2023) 13:15889. doi: 10.1038/s41598-023-43221-8 37741880 PMC10517978

[125] Sharma M Castro-Piedras I Rodgers AD Pruitt K. Genomic profiling of DVL-1 and its nuclear role as a transcriptional regulator in triple negative breast cancer. Genes Cancer. (2021) 12:77–95. doi: 10.18632/genesandcancer.217 34659647 PMC8513712

[126] Iba T Nisio MD Levy JH Kitamura N Thachil J. New criteria for sepsis-induced coagulopathy (SIC) following the revised sepsis definition: a retrospective analysis of a nationwide survey. BMJ Open. (2017) 7:e017046. doi: 10.1136/bmjopen-2017-017046 PMC5623518 28963294

[127] Li J Zhang Y Zhang D Li Y. The role of long non-coding RNAs in sepsis-induced cardiac dysfunction. Front Cardiovasc Med. (2021) 8:684348. doi: 10.3389/fcvm.2021.684348 34041287 PMC8141560

[128] Gentzel M Schambony A. Dishevelled paralogs in vertebrate development: redundant or distinct? Front Cell Dev Biol. (2017) 5:59. doi: 10.3389/fcell.2017.00059 28603713 PMC5445114

[129] Alshahrani SH Rakhimov N Rana A Alsaab HO Hjazi A Adile M. Dishevelled: An emerging therapeutic oncogene in human cancers. Pathol - Res Pract. (2023) 250:154793. doi: 10.1016/j.prp.2023.154793 37683388

[130] Ding C Wang J Wang J Niu J Xiahou Z Sun Z. Heterogeneity of cancer-associated fibroblast subpopulations in prostate cancer: Implications for prognosis and immunotherapy. Transl Oncol. (2025) 52:102255. doi: 10.1016/j.tranon.2024.102255 39721245 PMC11732565

[131] National Cancer Institute. DVL1 gene.,” Definitions. Qeios. (2020). doi: 10.32388/7Z8HQ4

[132] Gan X Wang J Xi Y Wu Z Li Y Li L. Nuclear Dvl, c-Jun, β-catenin, and TCF form a complex leading to stabiLization of β-catenin–TCF interaction. J Cell Biol. (2008) 180:1087–100. doi: 10.1083/jcb.200710050 PMC2290839 18347071

[133] Wang W Xu H Lin H Molnar M Ren H. The role of the cholinergic anti-inflammatory pathway in septic cardiomyopathy. Int Immunopharmacol. (2021) 90:107160. doi: 10.1016/j.intimp.2020.107160 33243604

[134] Zhang W Xu X Kao R Mele T Kvietys P Martin CM. Cardiac fibroblasts contribute to myocardial dysfunction in mice with sepsis: the role of NLRP3 inflammasome activation. PloS One. (2014) 9:e107639. doi: 10.1371/journal.pone.0107639 25216263 PMC4162616

[135] Liang Y Zhang R Biswas S Bu Q Xu Z Qiao L. Integrated single-cell transcriptomics reveals the hypoxia-induced inflammation-cancer transformation in NASH-derived hepatocellular carcinoma. Cell Prolif. (2024) 57:e13576. doi: 10.1111/cpr.13576 37994257 PMC10984103

[136] Sui S Tian Y Wang X Zeng C Luo OJ Li Y. Single-cell RNA sequencing gene signatures for classifying and scoring exhausted CD8+ T cells in B-cell acute lymphoblastic leukaemia. Cell Prolif. (2024) 57:e13583. doi: 10.1111/cpr.13583 38030593 PMC10905324

[137] Sui S Wei X Zhu Y Feng Q Zha X Mao L. Single-cell multiomics reveals TCR clonotype-specific phenotype and stemness heterogeneity of T- ALL cells. Cell Prolif. (2024) e13786. doi: 10.1111/cpr.13786 39675761 PMC11969251

[138] Yuan J Liao Y Zhang T Tang Y Yu P Liu Y. Integrating bulk RNA and single-cell sequencing data unveils efferocytosis patterns and ceRNA network in ischemic stroke. Transl Stroke Res. (2024). doi: 10.1007/s12975-024-01255-8 38678526

[139] Qin S Xie B Wang Q Yang R Sun J Hu C. New insights into immune cells in cancer immunotherapy: from epigenetic modification, metabolic modulation to cell communication. MedComm. (2024) 5:e551. doi: 10.1002/mco2.551 38783893 PMC11112485

[140] Ni G Sun Y Jia H Xiahou Z Li Y Zhao F. MAZ-mediated tumor progression and immune evasion in hormone receptor-positive breast cancer: Targeting tumor microenvironment and PCLAF+ subtype-specific therapy. Transl Oncol. (2025) 52:102280. doi: 10.1016/j.tranon.2025.102280 39805182 PMC11780959

[141] Ma Y Wang S Ding H. Bioinformatics analysis and experimental validation of cystathionine-gamma-lyase as a potential prognosis biomarker in hepatocellular carcinoma. BIOCELL. (2024) 48:463–71. doi: 10.32604/biocell.2024.048244

[142] Al Ghamdi ASM Alalawi RO Almalki ASA Alzahrani AM Nezamuldeen A Alshikhey MA. Critical analysis of laboratory biomarker discovery: bridging biomedical research with clinical diagnostics for early disease detection. J Ecohumanism. (2024) 3:24. doi: 10.62754/joe.v3i8.5314

[143] Liu DSK Puik JR Venø MT Mato Prado M Rees E Patel BY. MicroRNAs as Bile-based biomarkers in pancreaticobiliary cancers (MIRABILE): a cohort study. Int J Surg. (2024) 110:6518–27. doi: 10.1097/JS9.0000000000001888 PMC11486953 39041944

[144] Zhuge Y Zhang J Qian F Wen Z Niu C Xu K. Role of smooth muscle cells in Cardiovascular Disease. Int J Biol Sci. (2020) 16:2741–51. doi: 10.7150/ijbs.49871 PMC7586427 33110393

[145] Yang F Zhao LN Sun Y Chen Z. Levosimendan as a new force in the treatment of sepsis-induced cardiomyopathy: mechanism and clinical application. J Int Med Res. (2019) 47:1817–28. doi: 10.1177/0300060519837103 PMC6567749 30958071

[146] Tan Y Chen S Zhong J Ren J Dong M. Mitochondrial injury and targeted intervention in septic cardiomyopathy. Curr Pharm Des. (2019) 25:2060–70. doi: 10.2174/1381612825666190708155400 31284854

[147] O’Shea D Hodgkinson T Dixon J Curtin C O’Brien F. Development of an “off-the-shelf” gene therapeutic nanoparticle formulation for incorporation into biomaterials for regenerative medicine applications. Eur Cell Mater. (2024) 47:152–69. doi: 10.22203/eCM.v047a11

[148] Graham J Werba L Federico I Gonzalez-Fernandez T. Crispr strategies for stem cell engineering: a new frontier in musculoskeletal regeneration. Eur Cell Mater. (2023) 46:91–118. doi: 10.22203/eCM.v046a05

[149] Lamore KA. Constantly Evolving Journal: Reflecting on 2023Une revue en constante évolution : retour sur l’année 2023. Psycho-Oncol. (2024) 18:1–3. doi: 10.32604/po.2024.050518

[150] Dutheil S Bacqué M-F Lamore K. Promoting Health DemocracyFavoriser la démocratie en santé. Psycho-Oncol. (2024) 18:5–7. doi: 10.32604/po.2024.050924

[151] Nong Y Wei X Yu D. Inflammatory mechanisms and intervention strategies for sepsis-induced myocardial dysfunction. Immun Inflammation Dis. (2023) 11:e860. doi: 10.1002/iid3.860 PMC10187025 37249297

[152] Cai Y Hu W Pei Y Zhao H Yu G. Encoding biological metaverse: Advancements and challenges in neural fields from macroscopic to microscopic. Innovation. (2024) 5:100627. doi: 10.1016/j.xinn.2024.100627 38706956 PMC11068916

[153] Chen S. The impact of the human microbiome on gut health: recent research. Int J Clin Case Rep. (2024). doi: 10.5376/ijccr.2024.14.0004

[154] Kwa WT Sundarajoo S Toh KY Lee J. Application of emerging technologies for gut microbiome research. Singapore Med J. (2023) 64:45–52. doi: 10.4103/Singaporemedj.SMJ-2021-432 36722516 PMC9979803

[155] Cao Z Pang Y Pu J Liu J. Bacteria-based drug delivery for treating non-oncological diseases. J Controlled Release. (2024) 366:668–83. doi: 10.1016/j.jconrel.2024.01.020 38219912

[156] Wang Z. Research on microbial data modeling and disease prediction by fusion and integration of deep learning. Appl Math Nonlinear Sci. (2024) 9:20240884. doi: 10.2478/amns-2024-0884

[157] Vo D Singh SC Safa S Sahoo D. Boolean implication analysis unveils candidate universal relationships in microbiome data. BMC Bioinf. (2021) 22:49. doi: 10.1186/s12859-020-03941-4 PMC7863539 33546590

[158] Kumar AR Nair B Kamath AJ Nath LR Calina D Sharifi-Rad J. Impact of gut microbiota on metabolic dysfunction-associated steatohepatitis and hepatocellular carcinoma: pathways, diagnostic opportunities and therapeutic advances. Eur J Med Res. (2024) 29:485. doi: 10.1186/s40001-024-02072-3 39367507 PMC11453073

[159] Duan J Li Q Cheng Y Zhu W Liu H Li F. Therapeutic potential of Parabacteroides distasonis in gastrointestinal and hepatic disease. MedComm. (2024) 5:e70017. doi: 10.1002/mco2.70017 39687780 PMC11647740

[160] Rasha F Boligala GP Yang MV Martinez-Marin D Castro-Piedras I Furr K. Dishevelled 2 regulates cancer cell proliferation and T cell mediated immunity in HER2-positive breast cancer. BMC Cancer. (2023) 23:172. doi: 10.1186/s12885-023-10647-2 36809986 PMC9942370

[161] Benstead-Hume G Wooller SK Pearl FMG. [amp]]lsquo;Big data’ approaches for novel anti-cancer drug discovery. Expert Opin Drug Discovery. (2017) 12:599–609. doi: 10.1080/17460441.2017.1319356 28462602

[162] Chen B Butte A. Leveraging big data to transform target selection and drug discovery. Clin Pharmacol Ther. (2016) 99:285–97. doi: 10.1002/cpt.318 PMC4785018 26659699

[163] Christaki E Giamarellos-Bourboulis EJ. The beginning of personalized medicine in sepsis: small steps to a bright future. Clin Genet. (2014) 86:56–61. doi: 10.1111/cge.12368 24579691

[164] Itenov TS Murray DD Jensen JUS. Sepsis: personalized medicine utilizing ‘Omic’ Technologies—A paradigm shift? Healthcare. (2018) 6:111. doi: 10.3390/healthcare6030111 30205441 PMC6163606

[165] Sireesha V Fatima F Sultana S Kumar MSS Pravarsha Y Tatikonda RR. A comprehensive review on biomarker and its role in diseases. Cardiol Angiol Int J. (2024) 13:75–81. doi: 10.9734/ca/2024/v13i1395

[166] Rosati D Palmieri M Brunelli G Morrione A Iannelli F Frullanti E. Differential gene expression analysis pipelines and bioinformatic tools for the identification of specific biomarkers: A review. Comput Struct Biotechnol J. (2024) 23:1154–68. doi: 10.1016/j.csbj.2024.02.018 PMC10951429 38510977

[167] Petersen BK Yang J Grathwohl WS Cockrell C Santiago C An G. Deep reinforcement learning and simulation as a path toward precision medicine. J Comput Biol. (2019) 26:597–604. doi: 10.1089/cmb.2018.0168 30681362 PMC6590719

[168] Palma P Rello J. Precision medicine for the treatment of sepsis: recent advances and future prospects. Expert Rev Precis Med Drug Dev. (2019) 4:205–13. doi: 10.1080/23808993.2019.1626714

[169] Sharma M Castro-Piedras I Simmons GE Pruitt K. Dishevelled: A masterful conductor of complex Wnt signals. Cell Signal. (2018) 47:52–64. doi: 10.1016/j.cellsig.2018.03.004 29559363 PMC6317740

