# Supplementary material for: Interaction between post-tumor inflammation and vascular smooth muscle cell dysfunction in sepsis-induced cardiomyopathy
Source: Front Immunol. 2025 Apr 10;16:1560717. doi: 10.3389/fimmu.2025.1560717 (PMC12018406; doi:10.3389/fimmu.2025.1560717)
Supplement: Supplementary file 1 [file Table1.docx]

**Table.1**. Molecular Docking Results of Selected Compounds with DVL1 Protein (PDB ID: 6TTK) Using Autodock-Vina and Discovery Studio 2019

| **Protein**  **(Binding Site)** | **Compound** | **Vina(kcal·mol^-1^)** | **RMSD** | **DS(LibDockScore)** |
| --- | --- | --- | --- | --- |
| **DVL1(6TTK)** | **Digoxin** | -4.5 | 1.619 | 165.304 |
| **DVL1(6TTK)** | **Paromomycin** | -3.7 | 2.095 | 158.73 |
| **DVL1(6TTK)** | **Cabazitaxel** | -4.5 | 1.829 | 152.19 |
| **DVL1(6TTK)** | **Paclitaxel** | -4.6 | 1.516 | 151.868 |
| **DVL1(6TTK)** | **Streptomycin** | -4.6 | 2.614 | 148.925 |
| **DVL1(6TTK)** | **Toposar** | -5.2 | 0.452 | 147.71 |

# This table presents the binding affinity and docking scores of various compounds interacting with the DVL1 protein, as determined by molecular docking simulations using Autodock-Vina and Discovery Studio 2019. The Vina score (expressed in kcal·mol-¹) reflects the binding affinity, where more negative values indicate stronger interactions between the compound and the protein.The RMSD (Root Mean Square Deviation) values provide insight into the stability and accuracy of the binding pose, with lower values indicating a more stable interaction. The DS(LibDockScore) from Discovery Studio 2019 represents the strength of interaction, with higher scores suggesting better binding affinity.
