# Supplementary material for: Interaction between post-tumor inflammation and vascular smooth muscle cell dysfunction in sepsis-induced cardiomyopathy
Source: Front Immunol. 2025 Apr 10;16:1560717. doi: 10.3389/fimmu.2025.1560717 (PMC12018406; doi:10.3389/fimmu.2025.1560717)
Supplement: Supplementary file 2 [file Table2.docx]

**Supplementary Methods**

**1.1 Analysis of DVL1 Gene Expression in Rectal Cancer**

The differential expression of DVL 1 genes in rectal cancer tissues and adjacent normal tissues was extensively analyzed using HPA data(1,2). ROC analysis was performed using the pROC package, 95% confidence intervals and area under the curve (AUC) were calculated, and ROC curves were plotted to assess the diagnostic potential of DVL1 gene expression in differentiating rectal cancer from normal tissue. This assessment was based on RNA-seq data corrected by TCGA and processed using the Firehose pipeline. Z-Score standardization was applied to identify any outliers.and the Wilcoxon rank sum test was used to assess differences in expression between tumor and standard samples.

Pairwise analyses were performed using data from the GTEx and TCGA databases, and Z-Score standardization was used to eliminate outliers. ROC analyses were performed to assess gene ground diagnostic performance. Calibration curves and goodness-of-fit tests were applied to validate the accuracy of the predictive models. The study also examined the relationship between six immune molecular subtypes, tumor characteristics,and prognosis. The significance of subtype proportions was determined using chi-square tests based on median groupings. The Kruskal-Wallis rank sum test was used to compare the differences in DVL1 expression among the molecular subtypes. In contrast, the chi-square test assessed the distribution of clinical variables among the different expression groups.

Kaplan-Meier survival analysis explored the association between DVL1 expression levels and patient survival outcomes. Detailed survival analyses were performed using the R survival package, and the survminer package assisted in determining the optimal cutoff point for defining high and low-expression groups. Log-rank tests were performed using the surfeit function to assess survival differences between groups with different DVL1 expression levels. In order to combine the results of multiple studies, a meta-analysis using a univariate Cox proportional risk model was performed, applying an inverse variance method to derive hazard ratios (HR). These were classified into HR < 1 and HR > 1 groups, indicating potential tumor suppressor or tumor promoter effects of the gene. Statistical analyses, including forest and funnel plot visualizations, were performed in R (version 4.3.2) using the Meta package, providing a clear representation of the combined effect size and assessing publication bias. While this classification approach offers a straightforward analysis, it does not explore the underlying biological mechanisms of the genes.

**1.2 GSEA/GSVA Enrichment Analysis of the DVL1 Gene**

This study used a stratified approach to identify significant changes in gene expression(1,3). Samples were divided into high and low-expression groups, with the top 30% of DVL1 expression levels designated as the high-expression group and the bottom 30% as the low-expression group(4,5). Differential expression analysis was performed using the limma package, calculating log2 fold change (log2FC) to identify genes with significant expression changes. Following this, gene set enrichment analyses were performed using the fgsea function in the fgsea package, utilizing gene sets from the KEGG database. Cellular annotation and functional enrichment analyses were performed using clusterProfiler, GSEA, and GO analyses to ensure the scientific validity and reliability of the enrichment analysis. Enrichment scores (ES) were calculated, followed by significance tests and adjustments for multiple comparisons. Gene sets with unadjusted p-values below 0.05 and adjusted p-values below 0.25 were considered biologically significant and subsequently visualized.

To further explore cellular states within tumor cells, functional analysis was performed using the CancerSEA platform, which brings together data from a variety of sources to redefine 14 functional states. The z-score calculation method developed by Lee et al. was implemented using the GSVA package to analyze the set of functional state genes, and the results were normalized to z-scores. Pearson correlation analysis was then applied to assess the relationship between gene expression and these functional states to determine the correlation between DVL1 expression levels and z-scores for each functional state.

Furthermore, the gsva function in the GSVA package was utilized to compute the scores for 73 metabolic gene sets that were obtained from the KEGG database. Subsequently, these GSVA scores were applied to make a comparison of the activity levels of metabolic pathways between the highand low-expression groups by means of the limma package. This analysis was intended to illuminate the role of diverse metabolic pathways in the progression of the disease.

**1.3 Analysis of Correlations Related to the DVL1 Gene**

Using the Spearman correlation method (implemented via the cor). test function(6,7).Statistical significance of gene-protein pairs was achieved when the absolute correlation coefficient was >0.3 (more than 30%) and the p-value was <0.001. Scatter plots elucidated these strong relationships. RPPA data were also obtained from the TCPA database. Enter the name of the target gene in your gateway below box under Step 1 and then go to Step 3 as mentioned above at the very top of the page. The activity scores are calculated only based on the previously published research 14 pathways related to cancer were evaluated, which identified among Cancer-related Ten There were pathways frequently associated with the target gene, as assessed via Spearman correlation

Microbial abundance data were retrieved from TCMA database, and Spearman correlation analysis was used to explore the correlation between the abundance of microbes and gene expression levels in patients. We have implemented this approach to identify microbial species potentially involved in disease and provide new insights into the relationship between populations of microbes and host gene expression. Such analyses and integration will provide important insights into the more complex relationship between gene expression, protein levels, pathway activity, and microbial diversity.

**1.4 Analysis of Immune Infiltration Related to DVL1**

In this study, samples were divided into high and low-expression groups based on the median expression level of the DVL1 gene to ensure a high confidence separation between high and low-expression levels

(8,9). The Wilcoxon rank sum test, a robust non-parametric statistical test, was used to compare differences in immune cell populations between the two groups. This method is beneficial for datasets with different distributions as it does not require the assumption of normality. The analysis revealed distinct immune cell types with significantly different abundance between the high and low-expression groups. A heatmap sorting was performed to visualize the immune cell content across samples by increasing the expression of specified genes. The colour intensity gradient presented on the heatmap provided a visual sense of immune cell abundance, revealing trends and differences in immune infiltration between the two groups.

**1.5 DVL1 Gene Mutation Analysis**

This paper analyses the dependency scores of about 17,000 candidate genes using the CERES algorithm with the help of genome-wide CRISPR screening data from the DepMap portal site(10,11). The pan-cancer mutation landscape of the DVL1 gene was visualized using the plotmafSummary function from the maftools package. To investigate the relationship between gene expression levels and specific mutations, a substitution test was performed using the independence_test function from the coin package in R. This test was used to identify mutations with a frequency greater than 10% and a p-value less than 0.01. This test was used to identify mutations with a frequency greater than 10% and a p-value less than 0.01. Mutations with a frequency greater than 10% and a p-value less than 0.01 were considered to be significantly correlated and were subsequently visualized.

A GISTIC score-based approach was used to identify genomic copy number variants (CNVs) in the analysis of tumor copy number profiles in the TCGA-READ project. A total of 451 samples were processed to provide a detailed overview of CNVs, and the results were visualized in bar charts. Quantitative metrics of genomic alterations, including FGA, FGG and FGL, were calculated by measuring genomic distances in cloned regions. Analysis of variance (ANOVA) was applied in assessing differences between gene expression subgroups, and multiple comparisons were performed using the TukeyHSD method to further analyse significant findings. To examine the relationship between CNV scores and gene expression levels, scatter plots and Spearman's rank correlation coefficients were used; Spearman's rank correlation is a non-parametric method used to assess the monotonic relationship between two variables. Experimental data on copy number variation was obtained from the TCGA Genome Characterisation Centre by genome-wide microarray analysis. Gene-level CNV estimates were generated using the TCGA FIREHOSE pipeline and the GISTIC2 method. The Kruskal-Wallis test is another non-parametric method for comparing multiple groups to assess differences in gene expression from copy number states of -2 to 2. the Kruskal-Wallis test is a non-parametric method for assessing differences in gene expression from copy number states of -2 to 2. The Kruskal-Wallis test is a non-parametric method to evaluate differences in gene expression from -2 to 2 copy number states.

**1.6 Single-Cell Sequencing Analysis of Colorectal Cancer for a Specific Gene**

In this paper, we used single-cell gene expression data of rectal cancer from the GSE166555 dataset in the GEO database (1,12). Gene expression patterns of different cancer types at the single-cell level were visualized with the help of heatmaps generated by the pheatmap software package. Hierarchical clustering analysis using Euclidean distance and Ward's minimum variance method revealed the intrinsic patterns of gene expression and the conserved features among cancers. In order to reduce the dimensionality of the data while maintaining the topology, the UMAP (Uniform Mobility Approximation and Projection) technique was applied, which enabled clear visualisation of DVL1 expression patterns and helped to identify key biological differences. To assess the variation in gene expression between different cell types, the Kruskal-Wallis rank sum test was used. This non-parametric method is particularly suitable for identifying significant differences between independent groups with non-normal distributions. We used the AUCell score as a measure of pathway activity variability within individual cells and visualized and analysed the scores by UMAP. This approach provides a clear understanding of the distribution of pathway activity and helps to identify potential biological differences.

**1.7 Immunofluorescence**

Slides were rinsed twice with phosphate-buffered saline (PBS) at 37°C and then fixed with 3% paraformaldehyde and 0.1% Trillatone X-100 for 20 minutes at room temperature. After fixation, slides were incubated with primary antibodies for 12 hours at 4°C and then with the corresponding secondary antibodies for 1 hour at room temperature. Afterwards, the slides were rinsed with PBS. Cell nuclei were stained with 1 μg/mL Hoechst 33342 (blue). Images were acquired using an inverted confocal microscope (LSM 800, Zeiss) and further analysed using Adobe Photoshop.

**1.8 Flow Cytometry**

Cells were inoculated at a density of 1.0 × 10⁶ cells per dish in 10 cm ground culture dishes and incubated overnight. Once appressed, cells were treated with or without Digoxin for 24, 48, or 72 hours. After treatment, cells were harvested and washed twice with PBS. They are then fixed in 1 ml of 70% ethanol at -20°C overnight. Fixed cells were resuspended in 1.0 ml of 0.1% PBS containing 0.05 mg/ml Triton X-100 and 0.25 mg/ml propidium iodide and incubated at 37°C for 15 minutes. Flow cytometry analysis was performed using an excitation wavelength of 488 nm.

**1.9 qRT-PCR**

To isolate total RNA from cells, use 1 mL of Trizol Reagent per well and transfer the mixture to 1.5 mL EP tubes for 10 minutes of cell lysis. Add 200 μL of chloroform to each tube and centrifuge at 12,000 rpm at 4°C for 15 minutes. The upper aqueous phase is carefully transferred to a new tube and 400 μL of isopropanol is added to precipitate the RNA. After a series of centrifugation steps, the supernatant is removed, and the RNA precipitate is dissolved in 20 μL of DEPC-treated water. The RNA is reverse-transcribed to cDNA at 25°C for 5 minutes, 50°C for 15 minutes, 85°C for 5 minutes, and finally 4°C for 10 minutes. The cDNA is then diluted ten times to make the cDNA. The cDNA is then diluted tenfold. The cDNA is then diluted tenfold and amplified using real-time quantitative PCR according to the following protocol: 2 minutes at 50°C, 10 minutes at 95°C, followed by 40 cycles of 30 seconds at 95°C and 30 seconds at 60°C.

**1.10 CCK8 Assay for Cell Viability**

Logarithmic growth phase cells with approximately 80% confluence were selected for this assay. Each cell group was subjected to trypsin digestion followed by centrifugation to collect the cells, which were then resuspended in a complete medium. Cells were counted using a microscopic hematocrit counter and then inoculated into 96-well plates at a density of 4 x 10³ cells per well. After a 24-hour incubation period, the medium was replaced with fresh medium containing 10% CCK8 reagent. Incubate the plates at 37°C in an atmosphere of 5% CO₂ for 1-3 hours. Absorbance is read at 450 nm using an enzyme marker.

**1.11 Transwell Invasion Assay**

For Transwell invasion assays, 800 μL of F12K medium supplemented with 10% FBS was added to the lower chamber of a 24-well plate, and then the Transwell inserts were placed into the wells and equilibrated for 1 hour. Approximately 200 μL of the ground cell suspension for each experimental group was gently introduced into the upper chamber of the insert. The incubation device was incubated again at 37°C, 5% CO₂ for 24 hours,and repeated three times. After incubation, remove the insert and wash with PBS. Non-invasive cells were gently removed from the upper surface of the membrane using a sterile cotton swab. The membrane was fixed with 10% methanol for 20 minutes. After fixation, the penetrating cells are stained with 5% crystal violet solution for 20 minutes at room temperature. After staining, the membrane is washed with PBS and the insert is turned over for microscopic observation and imaging(13). The acquired data were analyzed using Image J software.

**1.12 Colony Forming Assay**

Cells were inoculated in 6-well plates at a density of 5.0×10² per well. After wall attachment, the cells were exposed to cardiac glycosides (CGs) or control agents for 10 days, and the medium was changed every 2 days. Cells were washed with phosphate-buffered saline (PBS) and fixed in 100% methanol for 10 minutes at room temperature. After fixation, the cells were stained with 0.5% crystal violet for 15 minutes at room temperature, and excess stain was rinsed off with double-distilled water (ddH₂O). Images of the colonies were taken using a camera, and then 500 μL of DMSO was added to each well to dissolve the crystal violet stain. Quantify the number of colonies using a microplate reader and measure absorbance at 595nm(14).

**1.13 Flow Cytometry for Apoptosis Detection**

Apoptosis was assessed using the Annexin V-FITC/PI Apoptosis Detection Kit (ThermoFisher Scientific, USA). Cells were rinsed with pre-cooled PBS and centrifuged to collect approximately 5 × 10⁵ cells. A 1× working solution was prepared by diluting a 5× binding buffer with double-distilled water. Cells were then resuspended in 500 μL of 1× binding buffer, 5 μL of Annexin V-FITC and 10 μL of ground PI were added. The mixture was gently vortexed and incubated at room temperature for 5 minutes away from light. Apoptotic cells were analysed by flow cytometry, with Annexin V-FITC detected in the FITC channel (Ex=488 nm, Em=530 nm) and PI detected in the PI channel (Ex=535 nm, Em=615 nm). FlowJo software was used for data analysis.

**References**

1. Liu B-X, Huang G-J, Cheng H-B. Comprehensive Analysis of Core Genes and Potential Mechanisms in Rectal Cancer. *Journal of Computational Biology* (2019) 26:1262–1277. doi: 10.1089/cmb.2019.0073

2. Huang M-Y, Yen L-C, Liu H-C, Liu P-P, Chung F-Y, Wang T-N, Wang J-Y, Lin S-R. Significant Overexpression of DVL1 in Taiwanese Colorectal Cancer Patients with Liver Metastasis. *IJMS* (2013) 14:20492–20507. doi: 10.3390/ijms141020492

3. Xu H, Ma Y, Zhang J, Gu J, Jing X, Lu S, Fu S, Huo J. Identification and Verification of Core Genes in Colorectal Cancer. *BioMed Research International* (2020) 2020:1–13. doi: 10.1155/2020/8082697

4. Li J, Guo G, Li J, Hao J, Zhang J, Guo Y, Yu H. The expression and significance of dishevelled in human glioma. *Journal of Surgical Research* (2014) 192:509–514. doi: 10.1016/j.jss.2014.06.034

5. Fabijanovic D, Zunic I, Martic TN, Skenderi F, Serman L, Vranic S. The expression of SFRP1, SFRP3, DVL1, and DVL2 proteins in testicular germ cell tumors. *APMIS* (2016) 124:942–949. doi: 10.1111/apm.12588

6. Shankavaram UT, Reinhold WC, Nishizuka S, Major S, Morita D, Chary KK, Reimers MA, Scherf U, Kahn A, Dolginow D, et al. Transcript and protein expression profiles of the NCI-60 cancer cell panel: an integromic microarray study. *Molecular Cancer Therapeutics* (2007) 6:820–832. doi: 10.1158/1535-7163.MCT-06-0650

7. Östlund G, Sonnhammer ELL. Quality criteria for finding genes with high mRNA–protein expression correlation and coexpression correlation. *Gene* (2012) 497:228–236. doi: 10.1016/j.gene.2012.01.029

8. Sørlie T, Perou CM, Tibshirani R, Aas T, Geisler S, Johnsen H, Hastie T, Eisen MB, Van De Rijn M, Jeffrey SS, et al. Gene expression patterns of breast carcinomas distinguish tumor subclasses with clinical implications. *Proc Natl Acad Sci USA* (2001) 98:10869–10874. doi: 10.1073/pnas.191367098

9. Yang H. “Systematic Evaluation of Gene Expression Data Analysis Methods Using Benchmark Data.,” In: Saberi Mohamad M, Rocha MP, Fdez-Riverola F, Domínguez Mayo FJ, De Paz JF, editors. *10th International Conference on Practical Applications of Computational Biology & Bioinformatics*. Advances in Intelligent Systems and Computing. Cham: Springer International Publishing (2016). p. 91–98 doi: 10.1007/978-3-319-40126-3_10

10. Dempster JM, Rossen J, Kazachkova M, Pan J, Kugener G, Root DE, Tsherniak A. Extracting Biological Insights from the Project Achilles Genome-Scale CRISPR Screens in Cancer Cell Lines. (2019) doi: 10.1101/720243

11. Bhattacharya A, Urzúa-Traslaviña CG, Van Vugt MATM, Fehrmann RSN. Abstract 5019: Eliminating copy number alteration effects in the gene essentiality data from the Cancer Dependency Map project. *Cancer Research* (2022) 82:5019–5019. doi: 10.1158/1538-7445.AM2022-5019

12. Wen L, Han Z, Du Y. Identification of gene biomarkers and immune cell infiltration characteristics in rectal cancer. *J Gastrointest Oncol* (2021) 12:964–980. doi: 10.21037/jgo-21-255

13. Wang H, Zhang Y. Effect of miR-590-3p/DKK1 Axis on the Progression of Wilms’ Tumour. *Archivos Españoles de Urología* (2024) 77:135. doi: 10.56434/j.arch.esp.urol.20247702.18

14. Zhang Y, Lin N, Liu X, Yao T. Dishevelled Segment Polarity Protein 3 (DVL3) Induced by Bacterial LPS Promotes the Proliferation and Migration of Prostate Cancer Cells through the TLR4 Pathway. *Archivos Españoles de Urología* (2024) 77:193. doi: 10.56434/j.arch.esp.urol.20247702.25

**Supplementary Figure**


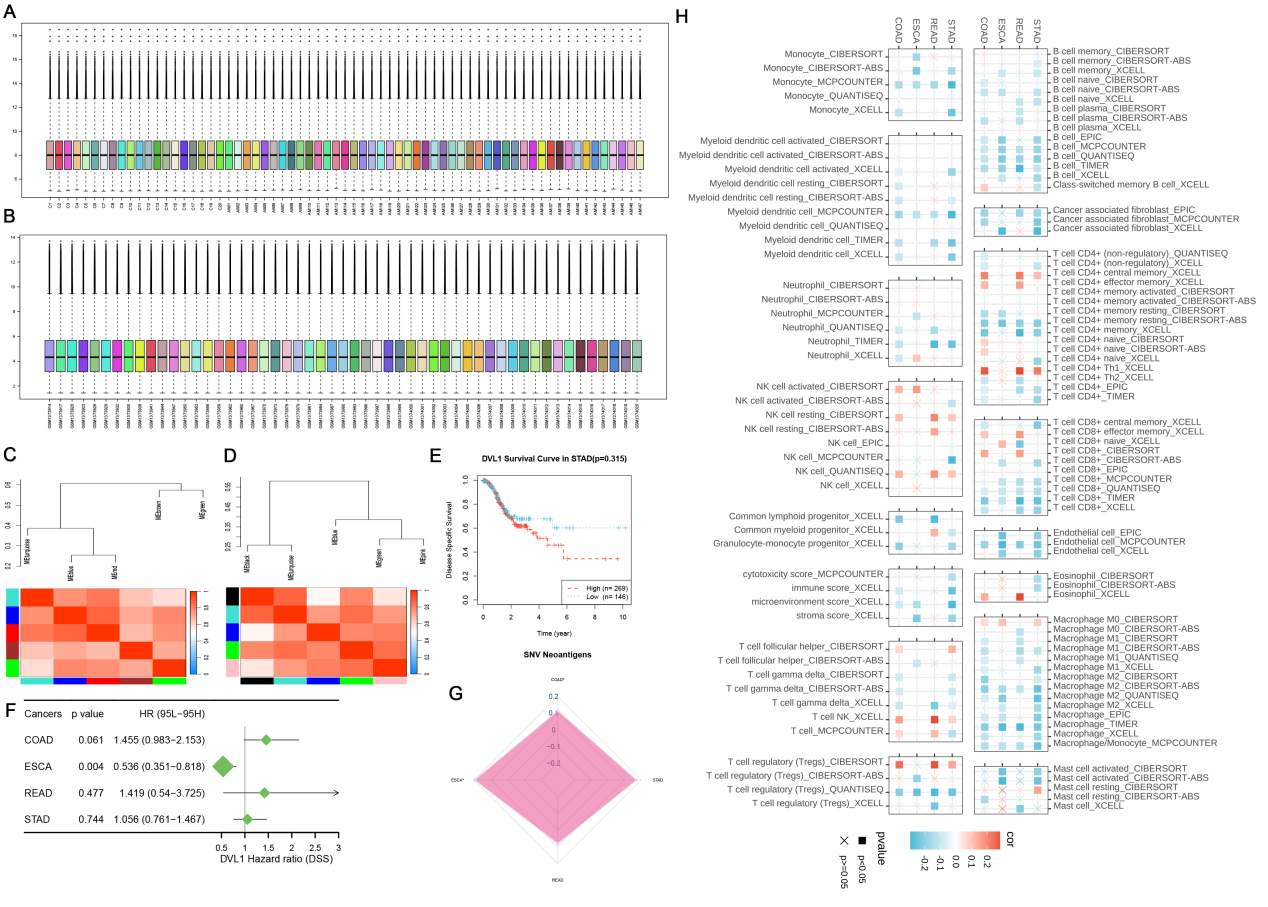


**Supplementary Figure 1. Expression Landscape of DVL1 in Gastrointestinal Tumors.**

(A & B) Normalization of septic cardiomyopathy samples. Boxplots display the expression levels of DVL1 after normalization across different samples. Each box represents the distribution of DVL1 expression within individual samples, showing the central tendency and spread of the data. The normalization process was carried out to adjust for potential technical variabilities and ensure comparability across samples.

(C & D) Weighted Gene Co-expression Network Analysis (WGCNA) module heatmaps for septic cardiomyopathy. The heatmaps illustrate the correlation between different gene modules and clinical traits. The hierarchical clustering dendrogram above each heatmap groups similar expression patterns, indicating potential co-regulated gene clusters. The color gradient represents the strength of module-trait relationships, with red indicating a strong positive correlation and blue a strong negative correlation.

(E) Survival prognosis curve for the DVL1 gene in STAD (Stomach Adenocarcinoma). Kaplan-Meier survival analysis was performed to assess the impact of DVL1 expression on patient survival. The red and blue lines represent high and low DVL1 expression groups, respectively, with the p-value indicating the significance of the difference in survival rates between these groups.

(F) Univariate prognosis analysis of the DVL1 gene in four gastrointestinal tumors (COAD, ESCA, STAD, and READ). Forest plots display the hazard ratios (HR) and 95% confidence intervals (CI) for DVL1 expression in each cancer type, indicating its potential as a prognostic marker. A HR greater than 1 suggests that high DVL1 expression is associated with a poorer prognosis, while a HR less than 1 indicates a better prognosis.

(G) Radar plot showing the distribution of Single Nucleotide Variant (SNV) neoantigens associated with DVL1 across four gastrointestinal tumors. The radar plot visually represents the neoantigen landscape, highlighting differences in the immune response potential across the cancer types.

(H) Distribution of immune cells in four gastrointestinal tumors based on DVL1 expression. Heatmaps display the correlation between DVL1 expression and the abundance of various immune cell types within the tumor microenvironment. Blue and red colors indicate negative and positive correlations, respectively, with stronger colors representing more significant relationships. The analysis provides insights into the immune-modulatory role of DVL1 across different gastrointestinal cancers.


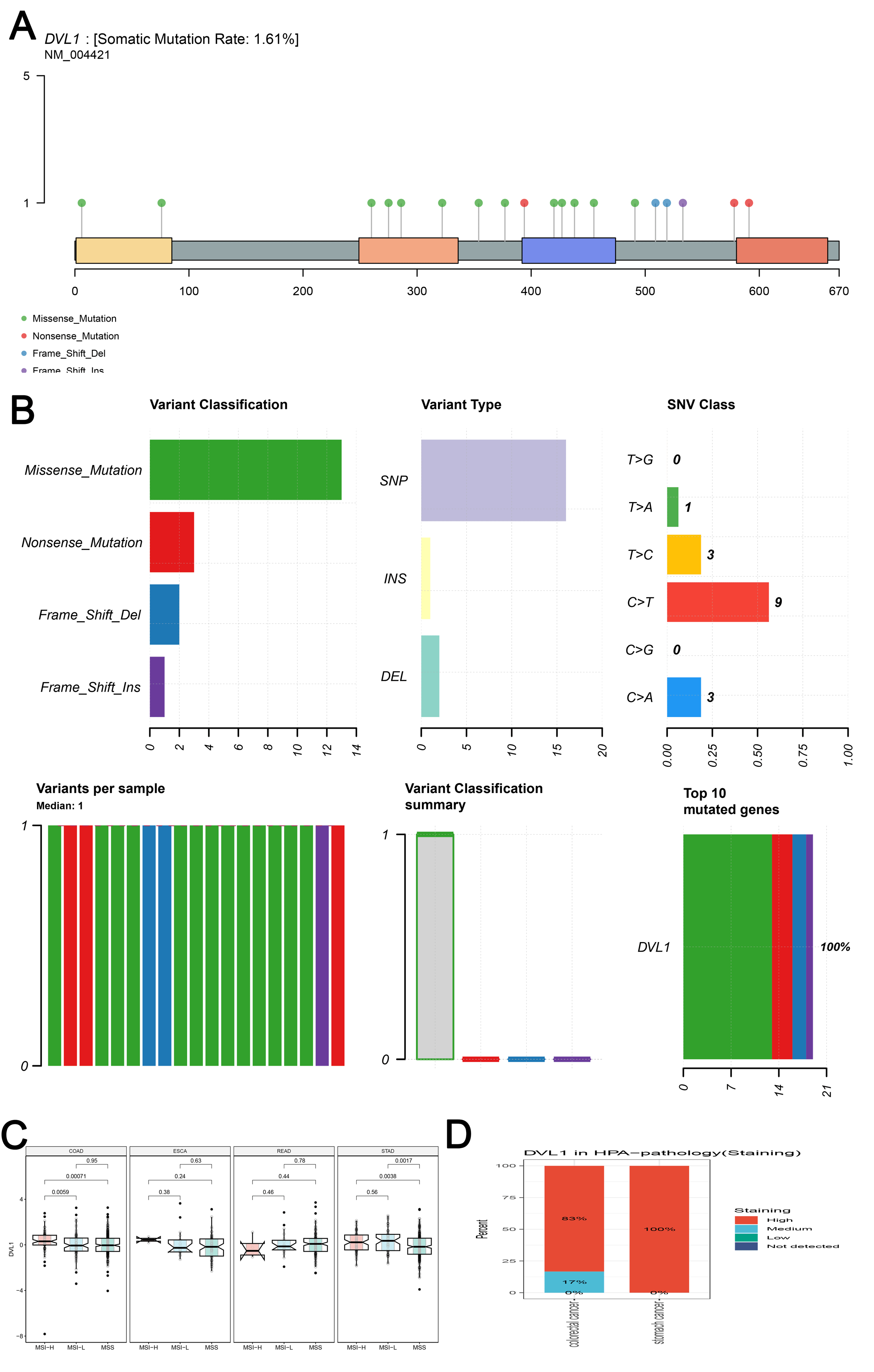


**Supplementary Figure 2. Expression and Mutation Analysis of DVL1 in Colorectal Cancer.**

(A) The somatic mutation rate of the DVL1 gene is depicted with a lollipop plot showing the distribution and types of mutations across the DVL1 coding region. The different types of mutations are color-coded: missense mutations (green), nonsense mutations (red), frameshift deletions (yellow), and frameshift insertions (purple). The somatic mutation rate is quantified as 1.61%, as indicated above the plot. This analysis provides an overview of the mutation hotspots within the DVL1 gene in colorectal cancer.

(B) Pan-cancer mutation analysis of the DVL1 gene across various tumor samples. The bar charts on the left display the classification of the mutations into missense, nonsense, frameshift deletions, and frameshift insertions. The middle panel categorizes the variants into single nucleotide polymorphisms (SNPs), insertions (INS), and deletions (DEL), with further breakdown into specific SNV classes such as C>T transitions, which predominate. The bottom panels present a summary of variant classification per sample and the top 10 most mutated genes, with DVL1 showing a 100% mutation frequency in the analyzed samples.

(C) Expression analysis of the DVL1 gene in relation to microsatellite instability (MSI) subtype across four types of gastrointestinal tumors. The boxplots show the expression levels of DVL1 stratified by MSI subtype, with statistical comparisons indicated. The analysis reveals significant differences in DVL1 expression between MSI subtypes in each tumor type, underscoring the gene's potential role in MSI-driven tumorigenesis.

(D) Protein expression of DVL1 in two gastrointestinal cancers based on Human Protein Atlas (HPA) pathology staining data. The bar chart shows the percentage of samples with different staining intensities (strong, medium, weak, not detected) across colorectal cancer and stomach cancer tissues. This analysis highlights the differential expression of DVL1 at the protein level, suggesting its involvement in the pathophysiology of these cancers.


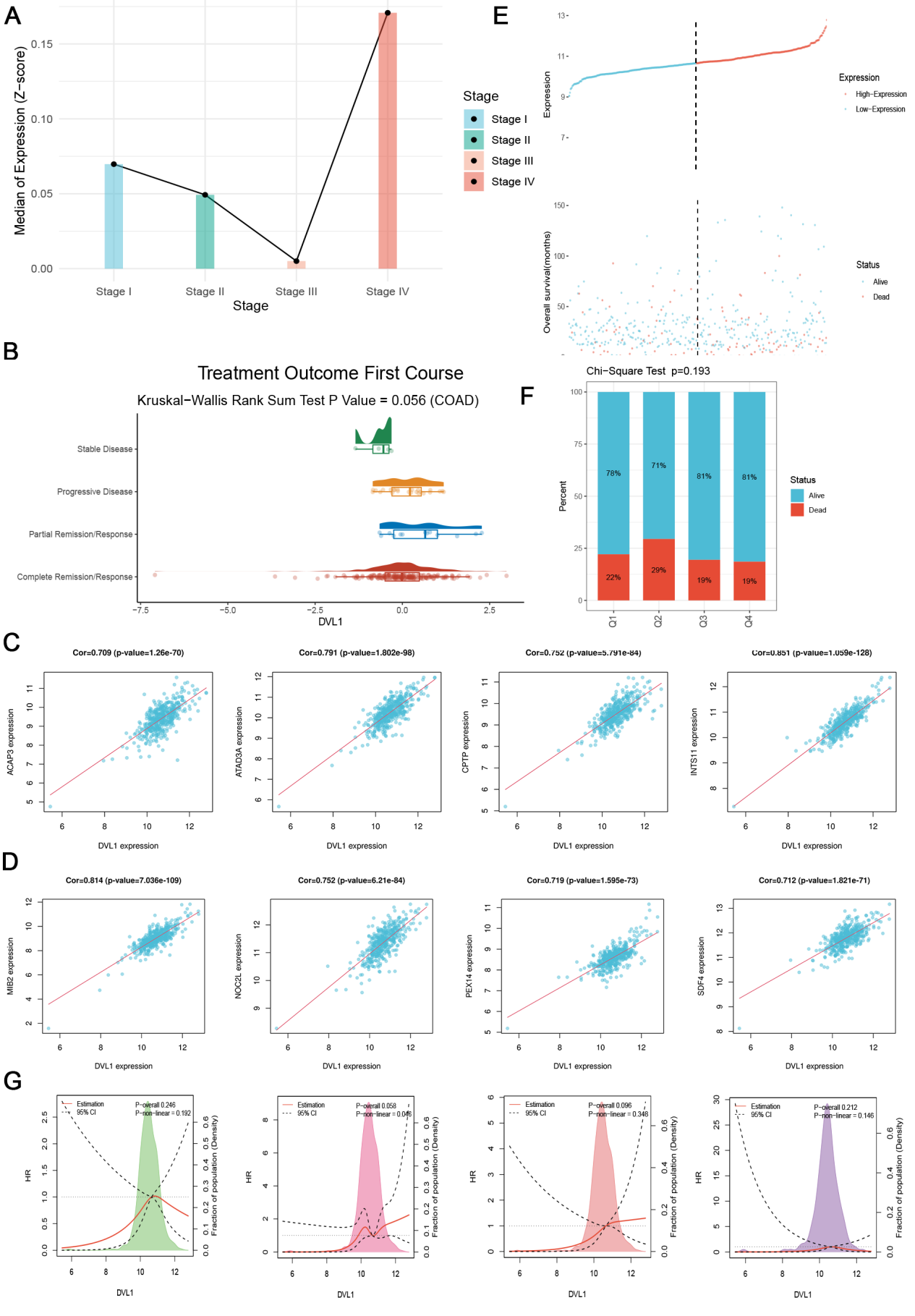


**Supplementary Figure 3. Analysis of DVL1 Gene-Related Differential Expression in COAD.**

(A) Expression differences of the DVL1 gene across different stages of COAD tumors are presented as a median expression level (Z-score) across Stages I-IV, demonstrating variable expression patterns with the highest expression in Stage IV tumors.

(B) Evaluation of DVL1 gene expression in relation to disease progression is depicted, with a Kruskal-Wallis Rank Sum Test revealing a p-value of 0.056, indicating a trend towards significance. The graph categorizes disease outcomes into Static Disease, Progressive Disease, Partial Remission/Response, and Complete Remission/Response, with DVL1 expression potentially linked to disease trajectory.

(C & D) Correlation analyses between DVL1 gene expression and various genes associated with COAD are shown. Multiple scatter plots display significant positive correlations (p-values < 1e-79 to 1e-128), suggesting that DVL1 expression is strongly related to these genes' expression levels across different samples.

(E) Survival prognosis curve stratified by DVL1 expression levels in COAD patients. The Kaplan-Meier curve demonstrates the association between high or low DVL1 expression and overall survival, though no significant difference is observed (Chi-square Test, p=0.193). Data is further stratified by patient status (Alive vs. Dead).

(F) Analysis of DVL1 expression across different tumor stages (I-IV) and patient status (Alive vs. Dead) is illustrated with a bar graph, showing the percentage distribution of alive and dead patients in each stage, indicating no significant correlation between DVL1 expression and survival (Chi-square Test, p=0.193).

(G) The restricted cubic spline method is applied to explore the potential non-linear relationship between DVL1 expression and four survival endpoints: Overall Survival (OS), Disease-Specific Survival (DSS), Progression-Free Interval (PFI), and Disease-Free Interval (DFI). Each spline plot demonstrates the relationship between DVL1 levels and the risk associated with these survival outcomes, with confidence intervals depicted to assess the statistical significance of the non-linearity.


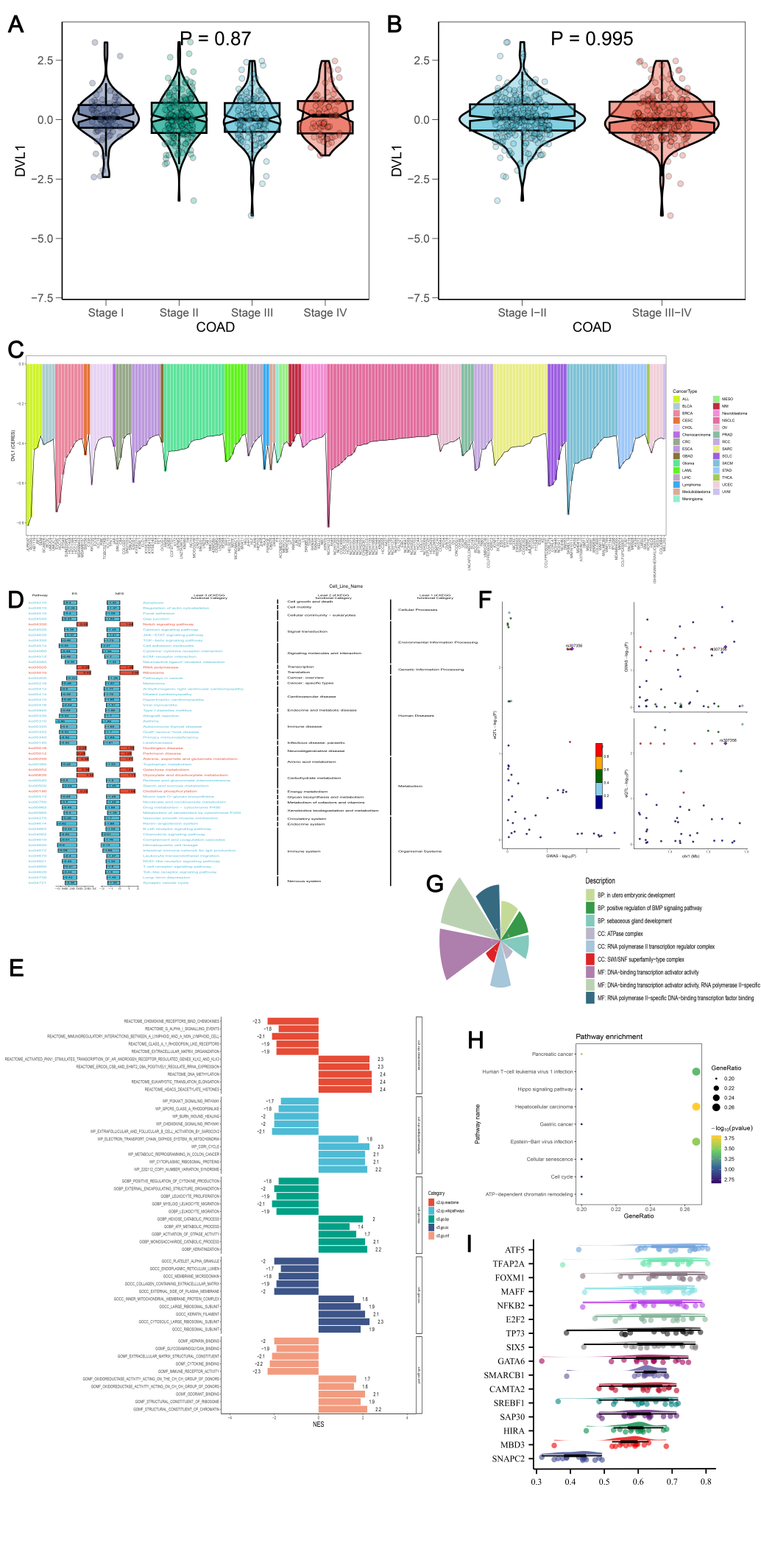


**Supplementary Figure 4. Analysis of DVL1 Gene Correlations in Colorectal Cancer (CRC).**

(A & B) Expression distribution of the DVL1 gene across different tumor stages in colorectal cancer (COAD). Panel (A) shows the expression levels across all four stages (Stage I to Stage IV), with statistical analysis indicating no significant difference (P = 0.87). Panel (B) further compares the expression between early stages (Stage I-II) and late stages (Stage III-IV), also showing no significant difference (P = 0.995). Violin plots are used to represent the distribution of expression levels, with data points overlaid to show individual sample values.

(C) Visualization of the top 200 cell lines from the DepMap database, showing CRISPR-Cas9 screening scores (CERES) for DVL1 gene dependency across various cancer types. This analysis highlights the essentiality of the DVL1 gene for the growth of different cancer cell lines, with each bar representing a specific cell line and color-coded by cancer type.

(D) KEGG pathway enrichment analysis for genes associated with DVL1 expression. The analysis identifies significantly enriched pathways, with a focus on those related to cancer biology. Pathways are ranked by enrichment score, with a color gradient indicating significance levels.

(E) Gene set enrichment analysis (GSEA) performed using the clusterProfiler package, comparing high DVL1 expression groups to low-expression groups. The bar plot shows the enrichment of multiple gene sets, with pathways related to cellular processes and cancer progression prominently featured. The analysis includes several hallmark gene sets, with enrichment scores and significance levels indicated.

(F) LocusCompare analysis visualizing the association between DVL1 gene expression and genetic loci. This panel highlights specific loci correlated with DVL1 expression, with scatter plots showing the relationship between expression levels and locus-specific genetic variation.

(G & H) GO and KEGG enrichment analysis of transcription factors differentially associated with DVL1 expression. The pie chart (G) illustrates the distribution of enriched Gene Ontology (GO) terms, while the dot plot (H) shows the KEGG pathway enrichment with gene ratios and significance levels. This analysis identifies biological processes and pathways potentially regulated by DVL1-associated transcription factors.

1. Friends analysis of the DVL1 gene, identifying transcription factors correlated with DVL1 expression. The scatter plot displays the correlation between DVL1 and selected transcription factors, with individual points representing different sample correlations. The analysis highlights transcription factors with strong associations, which may play a role in DVL1-mediated signaling pathways.


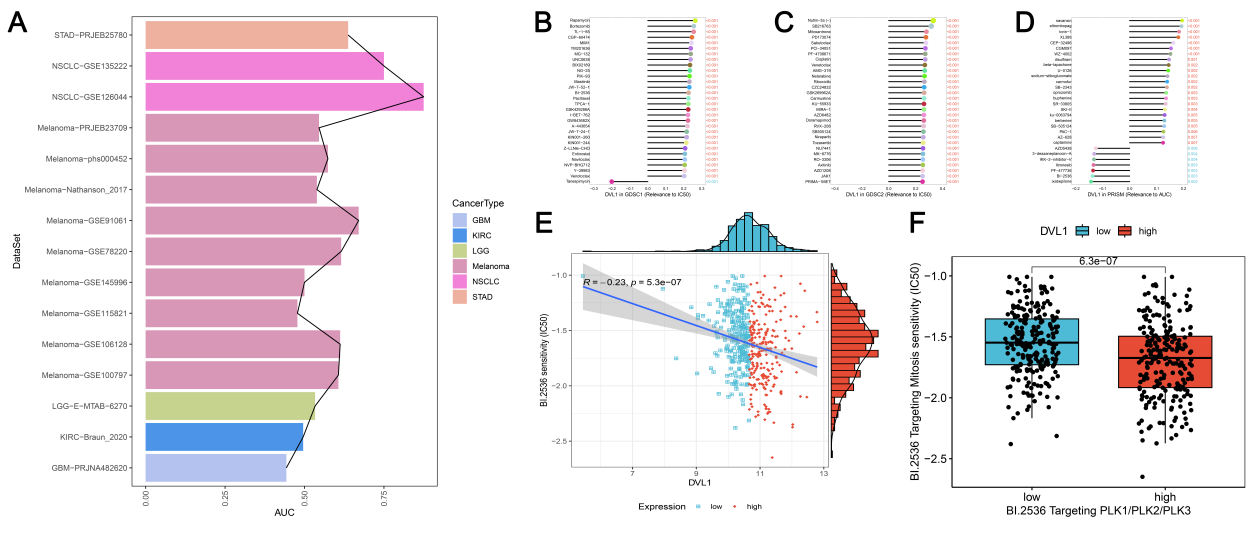


**Supplementary Figure 5. DVL1 Gene Drug Sensitivity and Immunotherapy-Related Analysis.**

(A) The ROC-AUC values for the prediction of immunotherapy response versus non-response across various cancer types based on DVL1 gene expression. The plot illustrates the area under the curve (AUC) for different cancer cohorts, including NSCLC (Non-Small Cell Lung Cancer), Melanoma, and others. The colored bars represent different cancer types with the respective AUC values plotted on the x-axis, indicating the performance of the predictive model.

(B-D) Spearman correlation analysis between DVL1 gene expression and drug sensitivity metrics (IC50 or AUC values) from the GDSC1, GDSC2, PRISM, and CTRP databases. These panels show the correlation coefficients for a range of drugs, with each bar representing a different compound. The drugs are ranked based on their correlation with DVL1 expression, and the corresponding p-values are shown to indicate statistical significance. The data sets used include large-scale drug screening data from multiple cancer cell lines.

(E) Correlation analysis between DVL1 expression and BI.2536 sensitivity. The scatter plot displays the relationship between DVL1 gene expression levels and the IC50 values of BI.2536 across cancer cell lines. The linear regression line is shown with a confidence interval, and the statistical significance of the correlation is annotated (p < 0.05). Blue and red colors distinguish cell lines with high and low DVL1 expression, respectively.

(F) Boxplot showing the distribution of BI.2536 IC50 values across samples stratified by high and low DVL1 expression. The data is divided into two groups based on the median expression of DVL1, and the corresponding BI.2536 sensitivity (IC50) is compared between these groups. Statistical significance is assessed using a t-test, with p-values indicated on the plot.


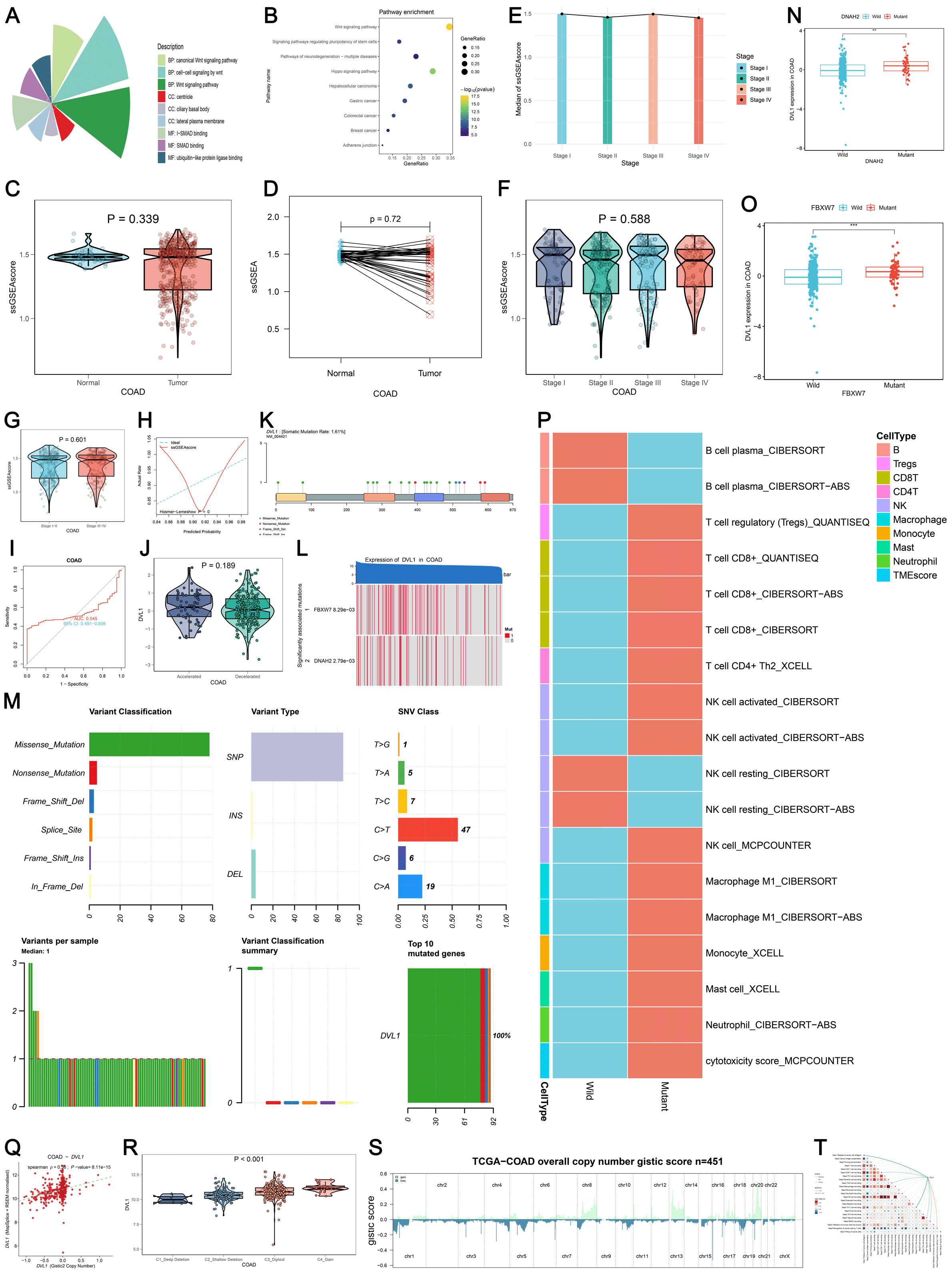


**Supplementary Figure 6. DVL1 Gene-Associated Differential Expression and Mutation Analysis.**

(A & B) Gene Ontology (GO) and Kyoto Encyclopedia of Genes and Genomes (KEGG) enrichment analysis of genes differentially expressed in association with DVL1. GO analysis highlights the biological processes, cellular components, and molecular functions enriched in these genes, while KEGG analysis indicates the associated signaling pathways.

(C & D) Differential expression of ssGSEA scores between tumor and normal tissues. Panel (C) shows non-paired sample analysis, while panel (D) presents paired sample analysis. The ssGSEA scores are used to quantify the expression levels of gene sets across different samples, demonstrating significant differences between tumor and normal tissues.

(E - G) ssGSEA score variation across different tumor stages. These panels illustrate how the ssGSEA score varies depending on the tumor stage, providing insights into the progression of tumor biology.

(H) Calibration curve and goodness-of-fit test for the predictive performance of the ssGSEA score in distinguishing between tumor and normal tissue samples. This panel shows the model’s calibration performance, indicating how well the predictions of the model agree with actual outcomes.

(I) Receiver Operating Characteristic (ROC) curve assessing the diagnostic efficacy of the ssGSEA score for differentiating between tumor and normal tissue samples. The ROC curve provides a measure of the sensitivity and specificity of the ssGSEA score as a diagnostic tool.

(J) Differential expression of DVL1 gene between biologically age-accelerated and decelerated groups. This panel compares the expression levels of DVL1 in individuals with different biological aging rates, highlighting its potential role in aging processes.

(K) Mutation map of the DVL1 gene. This panel displays the specific mutation sites identified within the DVL1 gene, providing a visual representation of the mutational landscape.

(L) Exploration of mutational events impacting gene expression. This analysis identifies specific mutations that may influence the expression levels of the DVL1 gene.

(M) Pan-cancer mutational landscape of the DVL1 gene. This panel showcases the mutation frequency and distribution of DVL1 across different cancer types, offering a comprehensive view of its mutation profile in various cancers.

(N & O) Analysis of DVL1 gene expression differences between DNAH2-mutant and wild-type samples. These panels compare the expression levels of DVL1 in the presence of DNAH2 mutations, highlighting potential interactions between these genes.

(P) Comparative analysis of tumor microenvironment components between mutant and wild-type genotypes using seven different algorithms. This heatmap shows how various microenvironmental components differ between the two genotypes, providing insights into the potential impact of mutations on the tumor microenvironment.

(Q) Spearman correlation between copy number variation (CNV) scores and gene expression levels. This scatter plot shows the relationship between CNVs and DVL1 expression, indicating how changes in gene copy number may affect expression levels.

(R) Differential expression of DVL1 across different CNV types. This panel illustrates how different types of CNVs (e.g., deletions and amplifications) influence the expression of the DVL1 gene.

(S) GISTIC2 score distribution for overall copy number changes in the TCGA-COAD dataset. This panel provides a genome-wide view of significant copy number alterations, as identified by GISTIC2 analysis, in colorectal cancer samples.

(T) Spearman correlation of CNV scores with gene expression levels, providing additional insights into the relationship between CNVs and gene expression in the dataset. This analysis helps to elucidate the genomic alterations that may drive changes in DVL1 expression.

**Supplementary Figure 7.**

**
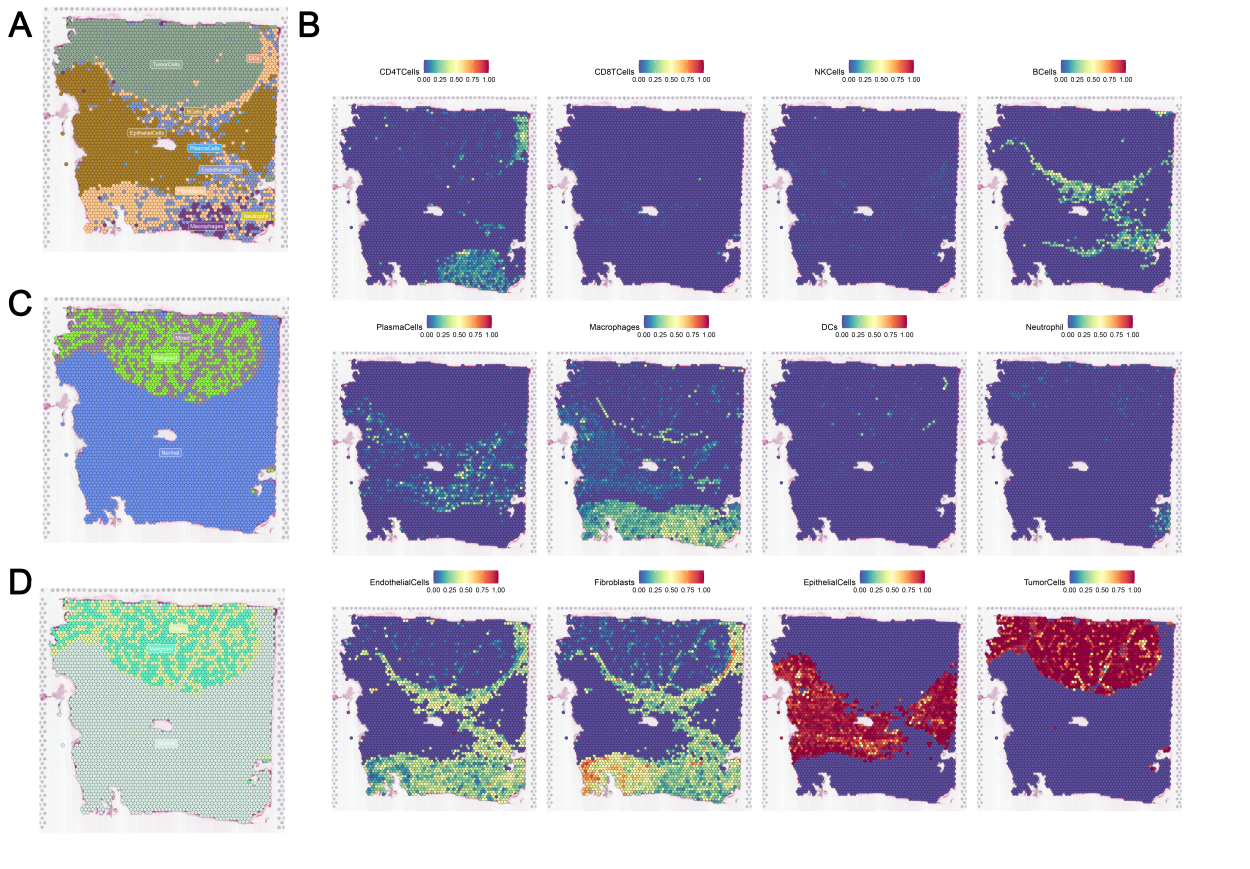
**

(A & D) Deconvolution and spatial localization of all cells post-transcriptomics analysis. The images display the spatial mapping of different cell types after deconvolution, highlighting distinct regions within the tissue, such as malignant, mixed malignant, and normal areas.

(E & F) Differential expression analysis of specific genes within malignant, mixed malignant, and normal regions. Heatmaps depict the relative expression levels of selected genes, showcasing the variation in expression across the different tissue regions, with a particular focus on malignant and mixed malignant areas.

(G-I) Spearman correlation between gene set AUC scores and microenvironment components at spatial resolution. Bar charts and correlation matrices present the relationship between the AUC scores for a specific gene set and the composition of the tissue microenvironment, demonstrating significant correlations that inform the understanding of tumor microenvironment interactions.
